# MiR-139-5p as a novel serum biomarker for recurrence and metastasis in colorectal cancer

**DOI:** 10.1038/srep43393

**Published:** 2017-03-06

**Authors:** Jinsei Miyoshi, Shusuke Toden, Kazuhiro Yoshida, Yuji Toiyama, Steven R. Alberts, Masato Kusunoki, Frank A. Sinicrope, Ajay Goel

**Affiliations:** 1 Center for Gastrointestinal Research, Center for Translational Genomics and Oncology, Baylor Scott & White Research Institute and Sammons Cancer Center, Baylor University Medical Center, Dallas, Texas, USA; 2 Department of Gastroenterology and Oncology, Institutes of Biomedical Sciences, Tokushima University Graduate School, Tokushima, Japan; 3 Department of Gastroenterological Surgery, Okayama University Graduate School of Medicine, Dentistry and Pharmaceutical Sciences, Okayama, Japan; 4 Department of Gastrointestinal and Pediatric Surgery, Division of Reparative Medicine, Institute of Life Sciences, Graduate School of Medicine, Mie University, Mie, Japan; 5 Division of Medical Oncology, Mayo Clinic and Mayo Cancer Center, Rochester, Minnesota, USA

## Abstract

Approximately 30–50% of colorectal cancer (CRC) patients who undergo curative resection subsequently experience tumor recurrence or metastasis. Although microRNAs (miRNAs) are a class of small noncoding RNAs frequently deregulated in various human malignancies, it remains unknown if these can help predict recurrence and metastasis in CRC patients. MiRNAs were initially screened using miRNA-microarray and miRNA-seq datasets with or without recurrence. Candidate miRNAs were then tested in two independent cohorts of 111 stage II/III and 139 stage I-III CRC patients, as well as serum samples and matched primary and metastatic liver tissues. An animal model of peritoneal dissemination was used to assess the oncogenic role of the target miRNA. Four candidate miRNAs were identified during the initial screening, and we subsequently validated upregulation of miR-139-5p in two independent clinical cohorts, wherein it associated with poor recurrence-free survival. Moreover, miR-139-5p were also upregulated in the serum of recurrence-positive CRC patients and yielded significantly shorter recurrence-free survival. Intriguingly, miR-139-5p was upregulated in metastatic liver tissues and negatively correlated with genes associated with epithelial-mesenchymal transition. Lastly, we showed that miR-139-5p overexpression enhanced peritoneal dissemination in a mouse model. In conclusion, we identified miR-139-5p as a novel biomarker for tumor recurrence and metastasis in CRC.

Colorectal cancer (CRC) is one of the most common malignancies worldwide, as well as a major cause of cancer-related deaths^1^,^2^,^3^. Approximately 60% of CRC patients have resectable disease when diagnosed and curative surgical resection followed by adjuvant chemotherapy is considered as the standard treatment strategy^4^. However, 30–50% of patients who undergo curative resection subsequently experience local and systemic recurrence^5^. Hence, it is not surprising that relapse and distant metastasis are the major causes of death in patients with CRC^6^,^7^. For patients with stage III CRC, several large-scale clinical trials have firmly established that the survival rate improves with post-operative adjuvant chemotherapy^6^,^8^. However, nearly 40% of the patients who were randomized to the placebo group did not develop recurrence even without chemotherapy, suggesting that a subgroup of stage III patients appear to have a low risk of relapse^7^,^9^. In contrast to stage III CRC patients, there is a well-recognized ongoing debate on whether adjuvant chemotherapy benefits patients with stage II CRC^10^. Without *a priori* of the stage II CRC patients that require adjuvant chemotherapy, this could lead to an overtreatment of patients with therapies that have severe adverse effects^11^. Therefore, there is a clear need for biomarkers which could identify patients with high risk of CRC relapse, so that these selective stage II/III CRC patients can undergo personalized treatments. Moreover, development of non-invasive blood-based markers for predicting cancer recurrence and metastasis can significantly improve the prognosis of high risk patients.

MicroRNAs (miRNAs) are a class of small non-coding RNA, approximately 21–23 nucleotides in length, that regulate target gene expression through transcriptional interference or translational inhibition^12^. MiRNAs play crucial roles in diverse cellular biological processes, including differentiation, proliferation, migration and survival of cells^13^,^14^. The accumulated data shows that up to 30% of genes (or mRNAs) are regulated by miRNAs and act as master regulators of gene expression for many critical biological pathways^15^. Interestingly, previous studies demonstrated that expression of miRNAs is often dysregulated in several cancers including CRC^16^. MiR-139-5p is located within the second intron of the phosphodiesterase 2 A (PDE2A) gene on chromosome 11q 13.4^17^. Several targets of miR-139-5p include Rho-Kinase 2 and c-Fos in hepatocellular carcinoma (HCC), type 1 insulin-like growth factor receptor (IGF-1R) in CRC^18^,^19^,^20^ and signaling pathways such as TGF-β, Wnt, Rho, and APK/PI3K in breast cancer^17^ have been known to be deregulated. However, the role of miR-139-5p in colorectal cancer pathogenesis and its clinical significance in this malignancy remains unclear.

In this study, using a comprehensive miRNA biomarker discovery process, followed by validation in two independent clinical cohorts, we for the first time have identified that miR-139-5p is a novel biomarker for tumor recurrence and metastasis in CRC. Furthermore, we have also discovered that serum miR-139-5p expression levels were significantly higher in patients with recurrence compared to recurrence-free patients. Finally, to evaluate its oncogenic role, in a mouse model of CRC peritoneal metastasis, we observed that miR-139-5p overexpression promoted metastasis.

## Results

### Identification of candidate CRC recurrence-associated miRNAs

**Figure 1:**
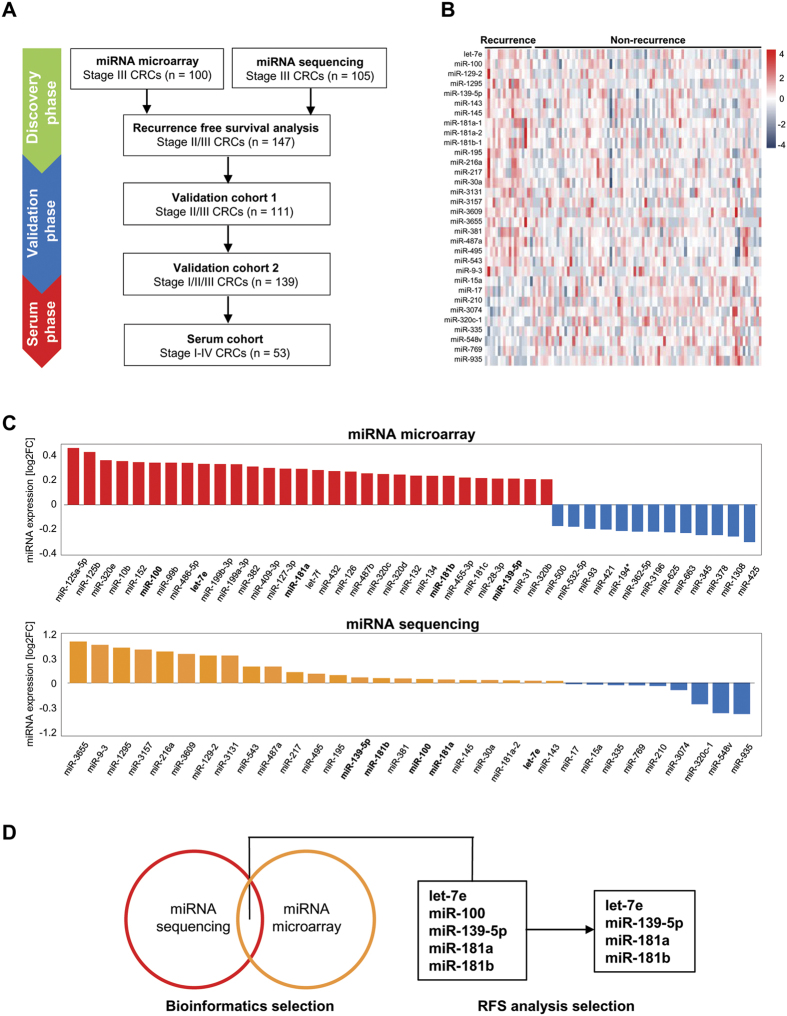
Selection of candidate miRNAs by miRNA sequencing and microarray analysis. (**A**) Strategy for the identification of CRC recurrence-specific miRNAs. (**B**) Heat map of miRNA sequencing expression by the TCGA data. (**C**) Ranking of miRNAs which were significantly differentially expressed between recurrence positive and negative stage III patients by miRNA microarray in Mayo Clinic cohort and by miRNA sequencing in TCGA dataset cohort. (**D**) Five candidate miRNAs were significantly up-regulated in recurrence-positive CRC patients in both datasets. And 4 out of 5 miRNAs could differentiate CRC patients who experienced recurrence versus those who did not, by measuring RFS using Kaplan-Meier analysis in stage II/III CRC patients from the TCGA cohort.

To identify miRNA-based CRC biomarkers for recurrence, we first interrogated two independent miRNA microarray and miRNA sequencing datasets. We initially performed Affymetrix microarray analysis on 100 stage III patients with adjuvant FOLFOX treatment, of which 50 patients had recurred, while 50 did not, and we were able to generate data from all samples except for three who did not recur. The median age range for these 97 patients was 59 (25–81) years, of which 51 (52.6%) were male. There were 48 (49.5%) primary tumors in the distal colon and 49 (50.5%) in the proximal colon (Supplemental Table 1). Of the 2,221 miRNA probes analyzed via microarray analysis (Supplemental Data 1), a total of 30 miRNAs were significantly upregulated (logFC > 0.2) in CRC with versus no recurrence (Fig. 1C).

Next, we conducted a similar analysis using RNA-seq data derived from The Cancer Genome Atlas (TCGA) dataset of 105 stage III CRC patients that included 18 patients who relapsed and 87 who did not. In this patient cohort, the median age (range) for these 105 stage III patients was 65.2 (37–90) years, of which 55 (47.4%) were male. There were 66 (63.4%) primary colorectal tumors and 39 (36.5%) rectal tumors. There were a total of 23 miRNAs that were significantly upregulated in CRC patients who recurred vs. those that did not (Fig. 1C).

**Figure 2:**
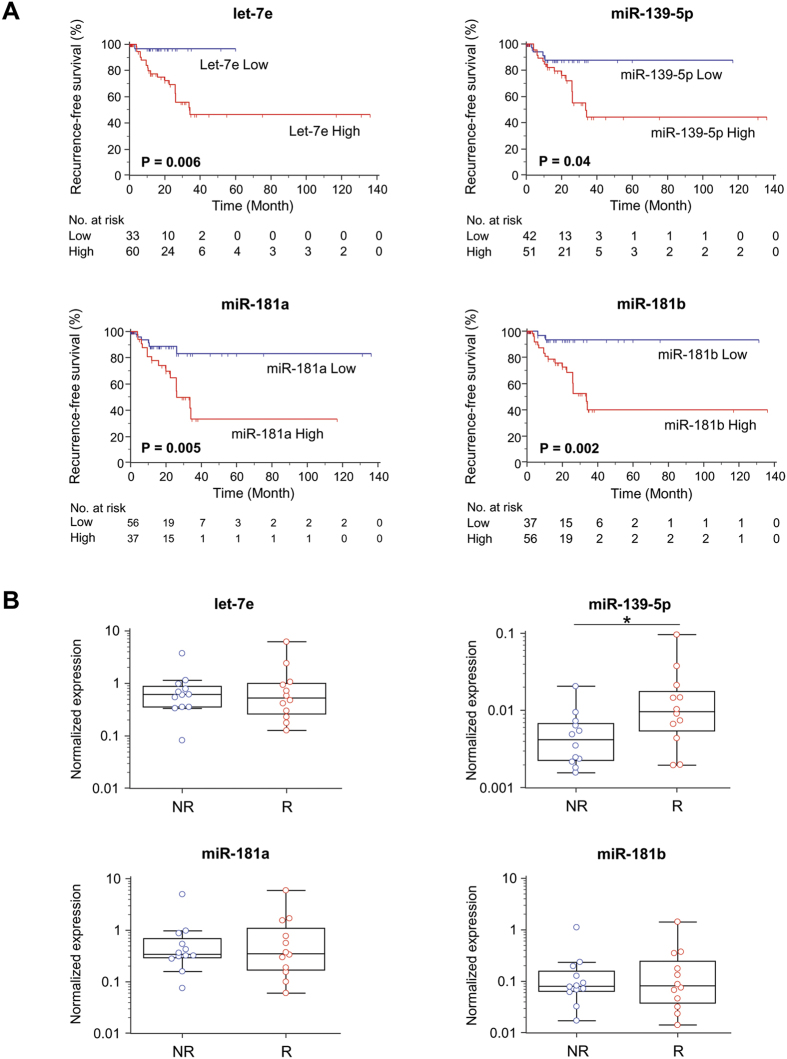
Selection of candidate miRNAs in the discovery phase. (**A**) Recurrence-free survival (RFS) analysis for 4 candidate miRNAs in the TCGA cohort of Stage II/III CRCs. (**B**) The expression levels of the candidate miRNAs measured by qRT-PCR in stage III CRC tissues which included 12 recurrence-positive and 12 non-negative CRC patients.

We assessed whether there was an overlap of upregulated miRNAs in the two datasets. We subsequently identified five miRNAs (let-7e, miR-100, -139-5p, -181a, -181b) that were significantly up-regulated in primary CRC tissues in relapse patients in both datasets. Next, using the TCGA stage II/III CRC dataset (Supplemental Table 2), we assessed whether the expression of these miRNAs could differentiate patients who recurred and those who did not using Kaplan-Meier analysis for recurrence-free survival (RFS). We defined RFS as the time from surgery until recurrence, which was considered as the clinical endpoint. Of the five candidate miRNAs analyzed, four miRNAs (let-7e, miR-139-5p, miR-181a, miR-181b) were able to differentiate those with vs. without CRC recurrence (Fig. 2A). The expression of miR-100 was unable to discern patients based on relapse (data not shown).

In order to test whether these four candidate miRNAs could be used as biomarkers for CRC recurrence, we measured the expression levels of these candidate miRNAs via qRT-PCR using RNA extracted from stage III CRCs; 12 patients recurred and 12 did not. We demonstrated that miR-139-5p was significantly higher in tumors from recurrent versus non-recurrent CRC patients (Fig. 2B), while no significant differences were observed in the levels of let-7e, miR-181a, or miR-181b in CRCs from patients who relapsed versus those who did not. Based upon these observations, we selected miR-139-5p for further testing as a candidate miRNA to serve as a biomarker for CRC recurrence.

### MiR-139-5p is upregulated in the primary tumors with recurrent CRC

To determine whether miR-139-5p is upregulated in the primary cancers of patients who had recurrence within three years of their cancer, we evaluated the expression of miR-139-5p in two independent clinical cohorts. The first patient cohort (Cohort 1) was comprised of 111 tissues from stage II/III CRC patients (31 CRC with and 80 without recurrence) and the second patient cohort (Cohort 2) consisted of 139 tissues from stage I-III CRC patients (38 CRC tissues and 101 without recurrence). For the Cohort 1, the median age of the patients was 67.5 years (range 28-92 years) and 65 of the tumors (58.5%) were located in the colon, while the remaining 46 (41.4%) were located in the rectum. In total, 60 cases were stage II (54.9%), while 50 cases were stage III (45.04%; Supplemental Table 3). For Cohort 2, the median age of the patients was 65.6 years of age (range 23–88 years) and 82 of the tumors (55.03%) were located in the colon, while the remaining 57 (44.9%) were located in the rectum. In total, 12 (8.63%) of the cases were stage I, 67 (48.2%) were stage II, and 60 (43.1%) were stage III (Supplemental Table 4).

**Figure 3:**
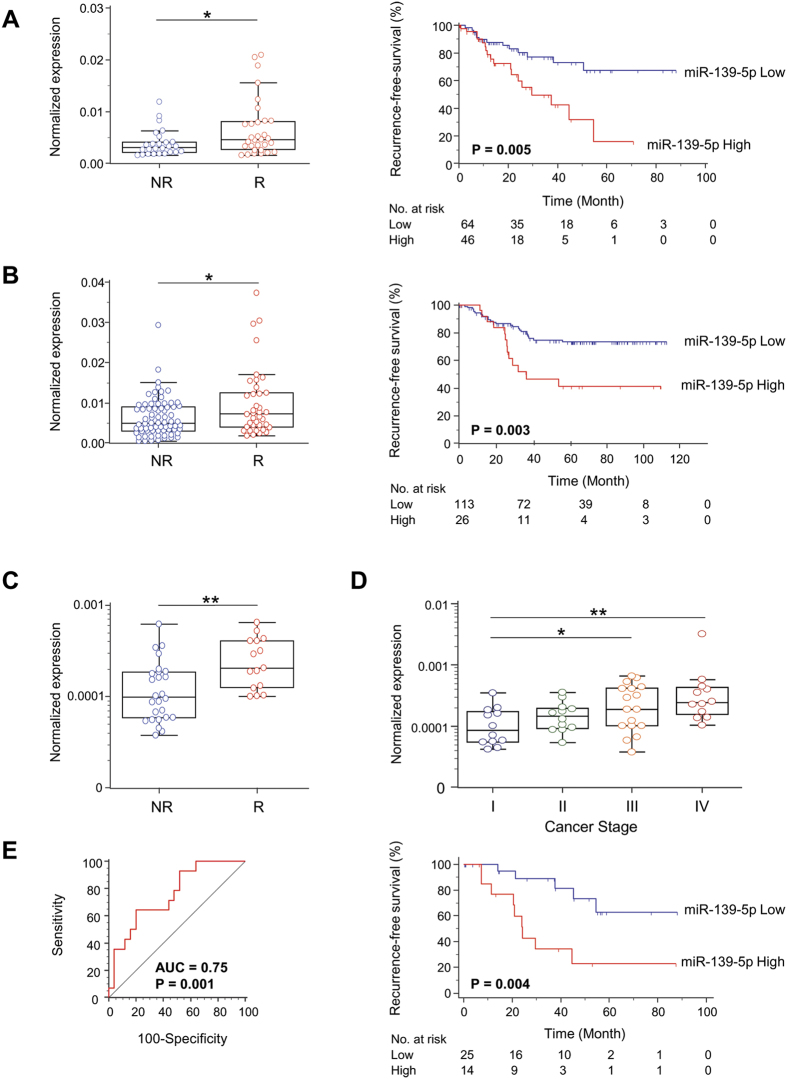
Mir-139-5p expression levels in two independent tissue validation cohorts and a serum validation cohort. (**A**) Validation cohort 1; 111 stage II/III CRC tissues with 31 recurrence-positive vs. 80 recurrence-negative cases. MiR-139-5p expression levels via qRT-PCR with recurrence vs. non-recurrence within 3 years after surgery, and RFS analysis of miR-139-5p. (**B**) Validation cohort 2; 139 stage I-III CRC tissues with 38 recurrence-positive vs. 101 recurrence-negative CRCs. MiR-139-5p expression levels via qRT-PCR with vs. without recurrence within 3 years after surgery, and RFS analysis of miR-139-5p. (**C**) Serum mir-139-5p expression levels in 41 stage I-III CRC patients with 14 recurrence-positive vs. 27 recurrence-negative. (**D**) Serum miR-139-5p expression levels of different stage in 53 stage I-IV CRC patients. (**E**) Area under the curve (AUC) value of distinguishing CRC recurrence from those without recurrence by serum miR-139-5p, and RFS analysis of serum miR-139-5p.

In Cohort 1, the expression of miR-139-5p in primary CRC tissues in patients who recurred within 3 years of surgery was significantly higher than in CRC tissues from patients who did not recur (P < 0.05; Fig. 3A). A similar finding was observed in Cohort 2; miR-139-5p expression was significantly higher in tumors from CRC patients who recurred within three years compared to those who did not experience tumor relapse (P < 0.05; Fig. 3B). Next, in order to evaluate whether miR-139-5p expression can predict CRC recurrence, we assessed the RFS using Kaplan-Meier analysis. Consistent with the results we obtained from the TCGA dataset used in our discovery phase, the patients with higher levels of miR-139-5p had significantly poorer RFS than those with lower expression of this miRNA in both cohorts (P = 0.005 and P = 0.003; log-rank test respectively; Fig. 3A,B). Moreover, we used the Cox proportional hazard regression model to determine whether miR-139-5p expression was an independent risk factor for RFS (Table 1). Univariate analysis of Cohort 1 revealed that high levels of miR-139-5p (P < 0.001), higher pathological T stage (T3/4; P = 0.01) and lymph node metastasis (P = 0.001) were significantly associated with poor RFS. The subsequent multivariate analysis confirmed these results and showed high miR-139-5p expression was one of independent markers for predicting early recurrence in CRC patients (HR = 2.63, 95% CI = 1.13–6.11 P = 0.02; Table 1). Likewise, in Cohort 2, the univariate analysis revealed high miR-139-5p (P = 0.002), high pathological T stage (T3/4; P = 0.01) and lymph node metastasis (P = 0.02) were significantly associated with poor RFS. The multivariate analysis adjusted for these parameters validated that high miR-139-5p expression was confirmed to be one of independent biomarkers for predicting early recurrence in CRC patients (HR = 2.30, 95% CI = 1.19–4.46 P = 0.01; Table 1). Collectively, the results from these experiments showed that miR-139-5p is a promising biomarker for CRC recurrence.

In addition, we assessed whether there were differences in miR-139-5p expression based on the metastasis sites in Cohort 1 patients. When compared between liver (n = 11), lung (n = 12) and other metastasis sites (n = 8), there were no statistical differences observed in miR-139-5p expression (Supplemental Fig. 1). Moreover, we assessed whether miR-139-5p expression was influenced by microsatellite instability (MSI) status in the TCGA dataset of 105 stage III CRC patients. Our analysis indicated that there was no difference between microsatellite stable (MSS; n = 84) and microsatellite unstable (MSI; n = 20) tumors (Supplemental Fig. 2).

### Serum miR139-5p is a tumor recurrence predictive biomarker in CRC

Next, we assessed whether the circulating levels of miR-139-5p levels in the serum of CRC patients are predictive of relapse in Cohort 3 (Supplemental Table 5). Serum miR-139-5p expression was significantly elevated in stage I-III CRC patients who recurred compared to those who did not recur (15 with recurrence versus 26 without recurrence; P = 0.002; Fig. 3C). Furthermore, serum miR-139-5p levels in stage III and stage IV CRC patients were higher than those in stage I CRC patients (P = 0.04 and P = 0.003 respectively; Fig. 3D). We then generated a ROC curve to evaluate the accuracy of miR-139-5p as a serum biomarker for the detection of CRC recurrence. An AUC value of 0.75 for serum miR-139-5p indicated that the expression of this miRNA could distinguish CRC patients with versus those without tumor recurrence (95% CI = 0.59–0.87 P = 0.001, sensitivity = 64% specificity = 80%; Fig. 3E). To further evaluate the predictive value of serum miR-139-5p levels for relapse in stage I-III CRC patients, we assessed RFS using Kaplan-Meier analysis. As expected, the patients with higher serum levels of miR-139-5p had a significantly shorter RFS than those with lower expression of this miRNA (P = 0.004; log-rank test; Fig. 3E). Collectively, our results showed that miR-139-5p expression in tissue, as well as in serum, is a potential of recurrence I CRC patients.

### MiR-139-5p is upregulated in liver metastatic CRC compared to primary CRCs

**Figure 4:**
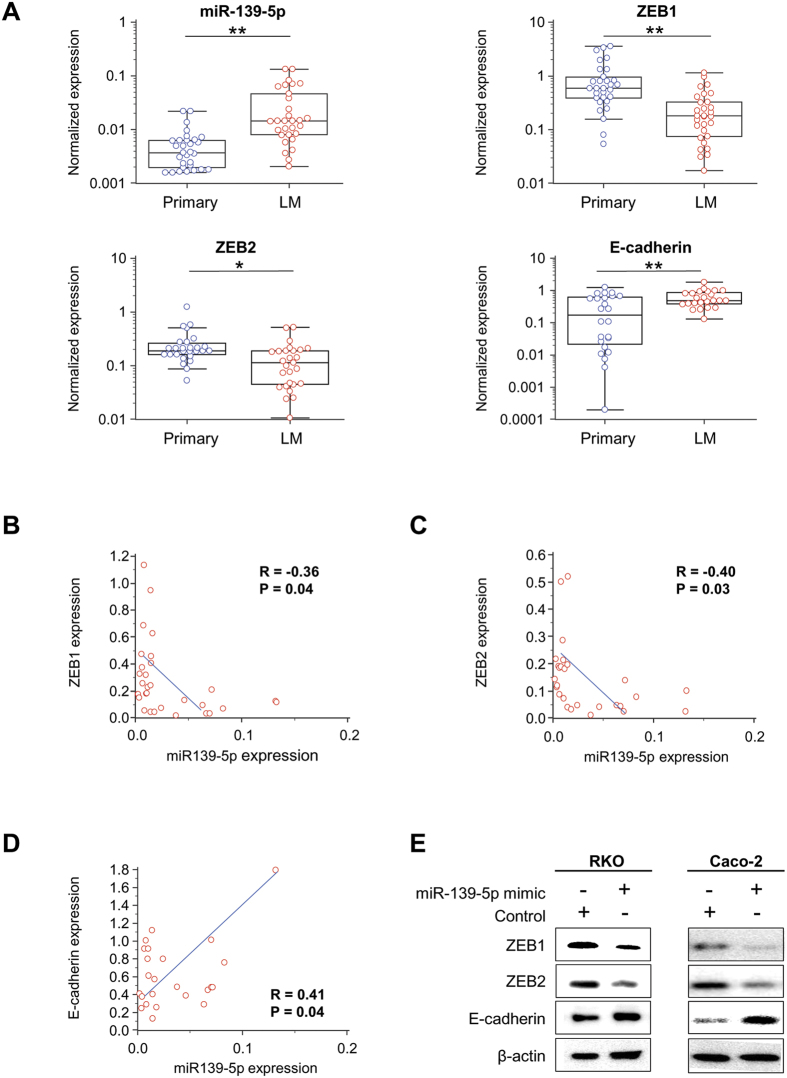
The expression levels of miR-139-5p and EMT-related genes (ZEB1, ZEB2, and E-cadherin) with primary CRC tissue vs. matched liver metastasis. (**A**) Expression levels of miR-139-5p and mRNAs associated with epithelial-to-mesenchymal transition (EMT)-related genes (ZEB1, ZEB2, and E-cadherin) in primary CRC tissue vs. matched liver metastasis tissues. (**B**–**D**) MiR-139-5p/target gene (ZEB1, ZEB2, E-cadherin) expression correlation in CRC liver metastasis tissues. (**E**) Protein expression (western blotting) of ZEB1, ZEB2, and E-cadherin with miR-139-5p overexpressing cells vs. controls.

Next, in order to evaluate our hypothesis that miR-139-5p is involved in the metastatic process in CRC, we analyzed the expression levels of miR-139-5p in 30 pairs of primary CRC and their matching metastatic liver tumors. Intriguingly, miR-139-5p was significantly upregulated in metastatic CRCs in the liver compared to primary CRCs (P = 0.0006; Fig. 4A). Due to these differences in levels of circulating miR-139-5p between the tumor and metastatic tissue, we decided to investigate the hypothetical role of miR-139-5p as an oncogene, with the ability to enhance epithelial-to-mesenchymal transition (EMT), a process whereby epithelial cells lose ability to migrate and gain adherence and polarization^21^. Moreover, interestingly a recent study showed that EMT is not a rate-limiting step for metastasis and can be disposable for metastatic processes^22^. Interestingly, ZEB1 and ZEB2, two transcription factors involved in this process, are downstream targets of miR-139-5p^23^. Therefore, we evaluated the association between miR-139-5p and these two EMT-associated genes. As expected, the expression of both ZEB1 and ZEB2 were significantly downregulated in metastatic liver tissues compared to primary CRC tissues (P = 0.001, P = 0.02 respectively; Fig. 4A), while E-cadherin was significantly upregulated in metastatic liver tissues versus primary CRC tissues (P = 0.002; Fig. 4A). Furthermore, the expression of ZEB1 and ZEB2 inversely correlated with miR-139-5p expression (r = −0.36, P < 0.05 and r = −0.401, P < 0.05 respectively; Fig. 4B,C), while E-cadherin showed a positive association with miR-139-5p (r = 0.41, P < 0.05; Fig. 4D). Collectively, these data indicate that miR-139-5p is involved in EMT and upregulation of circulating miR-139-5p may promote colonization of cancer cells and initiate metastasis.

### MiR139-5p regulates EMT-related genes *in vitro*

In order to confirm that miR-139-5p, a noncoding RNA, regulates EMT-related genes (ZEB1, ZEB2, E-cadherin), we overexpressed miR-139-5p by transiently transfecting CRC cell lines with miR-139-5p mimics or scrambled controls, and evaluated the protein expression of these downstream target genes. First, we assessed the expression of miR-139-5p in several CRC cell lines and selected two CRC cell lines with lowest miR-139-5p expression, RKO and Caco-2 (Supplemental Fig. 3). Western blot analysis showed that overexpression of miR-139-5p in CRC cell lines resulted in reduced ZEB1 and ZEB2 protein expression, while E-cadherin was upregulated compared to negative control transfected cells (Fig. 4E). These data are consistent with previous studies^23^,^24^ and confirm that ZEB1 and ZEB2 are regulated by miR-139-5p.

### Overexpression of miR-139-5p induces peritoneal metastasis

**Figure 5:**
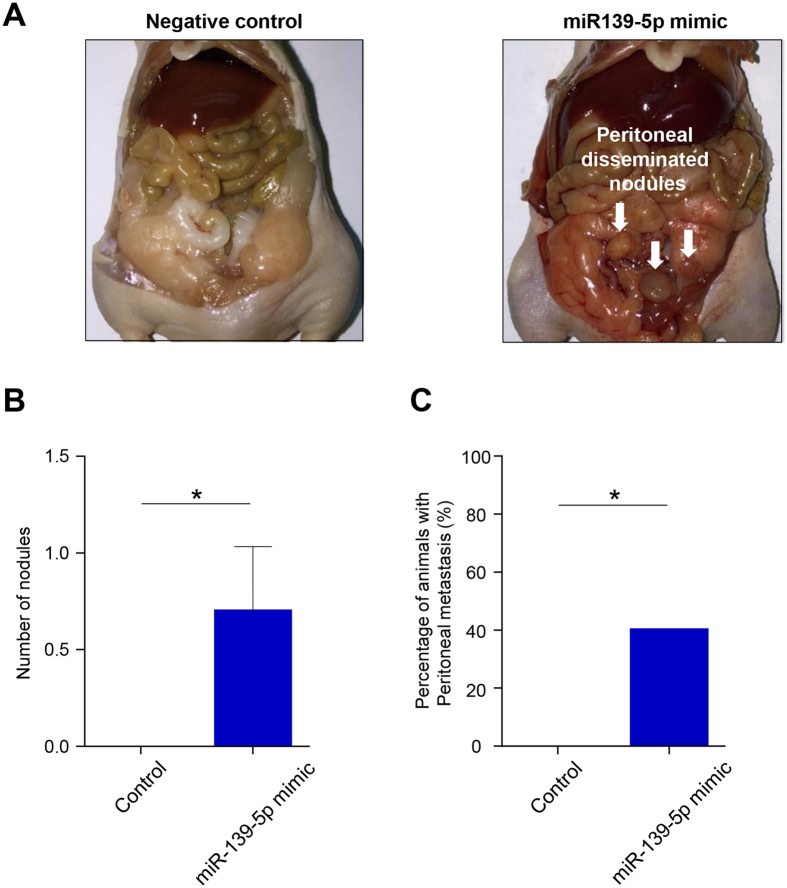
A mouse model of CRC peritoneal metastasis. (**A**) Representative images of nude mice injected with miR-139-5p overexpressing cells vs. negative control cells. **(B**) Number of disseminated nodules with miR-139-5p overexpressing cells vs. negative control cells. (**C**) Percentage of animals with peritoneal metastasis for those injected with miR-139-5p overexpressed cells vs. negative control cells analyzed by Chi-square test.

To confirm whether CRC cells with high levels of miR-139-5p are more likely to attach themselves to the metastasis sites compared to those cells with lower expression of this miRNA, we overexpressed miR-139-5p CRC cell lines and used these cells in a mouse model of peritoneal metastasis. This model was chosen for ease and the fact that the abdominal cavity is the second most common site of recurrence in patients with CRC and is a life-threatening event in CRC patients^25^. Immune deficient mice were injected intraperitoneally with 3 × 10^6^ cells transfected with either miR-139-5p mimic or scrambled control (n = 11 each group) and all mice were sacrificed 60 days post-injection. The assessment of the internal cavity of mice showed that miR-139-5p-overexpressing cells had a significantly greater number of disseminated nodules than cells transfected with the scrambled control (P < 0.05; Fig. 5B). Furthermore, no mice in the control group developed any tumors (Fig. 5C), indicating that miR-139-5p enhances metastatic potential of CRC cells.

## Discussion

Colorectal cancer accounts for over 9% of all cancers, and death from metastasis and recurrence accounts for approximately 90% of all human cancer mortalities^26^. In this study, using comprehensive discovery approach, we identified a miRNA in the tumor and serum which could potentially be used as a predictive biomarker for CRC relapse. We then validated the biomarker potential of miR-139-5p to serve as a recurrence prediction biomarker in two independent clinical cohorts. Moreover, we showed that the expression of miR-139-5p is upregulated in metastatic tissues compared to matching primary tissues and it is inversely correlated with EMT-associated genes. Lastly, in an *in vivo* animal model we demonstrated that overexpression of miR-139-5p resulted in enhanced peritoneal metastasis.

MiRNAs are known to regulate multiple key signaling pathways in the process of carcinogenesis, tumor progression, invasion, and metastasis^27^. Due to their high abundance and stability, miRNAs are becoming attractive candidates to serve as cancer biomarkers. In this study, we used comprehensive miRNA microarray and RNA-seq based analyses to help uncover miRNAs that can function as a biomarker for CRC recurrence. In addition to this discovery step, we analyzed two independent patient cohorts to validate that miR-139-5p is a potential biomarker for CRC recurrence. Supporting these results, several studies have reported that miRNAs such as miR-21, miR-29a, miR-34a, and miR-224 are deregulated during relapse and in metastatic CRC and were able to predict patients with high risk of recurrence^28^,^29^,^30^,^31^. However, these studies had several limitations, including inadequate sample size, lack of systematic and comprehensive discovery approach and lack of a validation patient cohort. The results of our study, together with the findings from these previous studies, collectively highlighted that the dysregulated miRNAs in primary CRCs may serve as potential candidates for predicting recurrence and metastasis.

Furthermore, we have also demonstrated that the expression of miR-139-5p in serum, not just tissue, appears to serve as a possible marker for CRC recurrence. Accumulating evidence from a number of studies suggest that miRNAs in blood are a desirable source for biomarkers, due to their stability in the blood despite the high activity of ribonuclease^32^,^33^. Generally, miRNAs are protected from the enzymatic degradation as they are packaged in extracellular vesicles such as exosomes, or bound to proteins and lipoproteins which significantly increase their stability in comparison to free circulating RNAs^34^,^35^,^36^. To date, several miRNAs including miR-21 and miR-200c have been reported to be deregulated in serum and could predict CRC patients with recurrence^37^,^38^. While in these previous studies the rationale for candidate miRNA selection was primarily based on their putative functions, in this study, we identified miR-139-5p as a CRC recurrence prediction biomarker using a systematic and comprehensive miRNA discovery strategy. In order to truly test the biomarker potential of circulating miR-139-5p, a prospectively collected longitudinal study is required. Therefore, we have begun a prospective collection of patient samples, both tissue and serum, and plan to assess the efficacy of miR-139-5p as a potential monitoring marker for CRC recurrence.

The dysregulation of miR-139-5p expression has been reported to occur in a number of cancers. In pancreatic cancer and ovarian granulosa cell tumor the expression of miR-139-5p was found to be upregulated, indicating its potential role as an oncogene^39^,^40^. In addition, serum levels of miR-139 were shown to be significantly increased in adrenocortical cancer^41^. Moreover, the expression of miR139-5p in peripheral blood was significantly higher in prostate cancer patients compared to patients with benign prostatic hyperplasia and healthy controls, and much higher expression of peripheral blood miR-139-5p was detected in prostate cancer patients with more advanced stage and more aggressive tumors^42^. The current study demonstrated that miR-139-5p was upregulated in primary cancer tissues of patients with recurrence compared to those who did not. Furthermore, we showed consistent upregulation of miR-139-5p in the serum samples of CRC patients with recurrence. We also revealed that the expression of miR-139-5p was upregulated in liver metastatic tissues compared to matching primary tissues, and illustrated in a mouse model that an overexpression of miR-139-5p in a CRC cell line resulted in higher peritoneal metastasis than the respective control cell line.

In contrast, there are few reports in various cancers that miR-139-5p may also act as a tumor suppressor. It was reported that miR-139-5p was downregulated in hepatocellular carcinoma^19^, parathyroid carcinoma^43^, and gastric cancer^44^ with *in vitro* data suggesting that the inhibition of miR-139-5p results in increased proliferation^45^. While these outcomes are in conflict with the results of the previous studies, there are several miRNAs that appear to behave similarly to miR-139-5p, acting as both oncogenes and tumor suppressors in different types of human cancers.

MiR-200c acts as a putative tumor-suppressor-miRNA, but it is well known that this miRNA is required for establishment of metastasis^46^. MiR-200c-ZEB1 axis is recognized as an important regulator of EMT^47^,^48^, but also has the capacity to colonize mesenchymal metastatic cells to the distant organs through stimulating these cells to become epithelial form. Intriguingly, circulating miR-200c levels in CRC patients were significantly higher than that of healthy controls. When CRC liver metastases were compared to matched primary CRC, miR-200c was overexpressed in liver metastases^38^. The present study showed that miR-139-5p functions similarly to miR-200c, i.e. both miRNAs are upregulated in primary tumors and in serum of patients who undergo tumor relapse. Mechanistically, miR-139-5p and miR-200c share the same EMT-associated downstream target genes such as ZEB1 and ZEB2, a possible explanation for their functional similarities. Therefore, we suspect that despite the fact that miR-139-5p may act as a tumor suppressor in early-stage cancers, it may play a significant role in initiation of metastasis in CRC.

In conclusion, for the first time, we have identified miR-139-5p as a potential novel biomarker for tumor recurrence in CRC patients. Furthermore, we showed that circulating miR-139-5p was able to discriminate patients who were going to recur within three years from those patients who did not undergo relapse during this time span. Our findings may have important implications for the development of personalized medicine, as this allows identification of those CRC patients who have a high risk of recurrence and require adjuvant chemotherapy, from those who may benefit from curative surgery alone.

## Materials and Methods

### Study design and clinical specimens

In order to identify novel miRNAs associated with cancer recurrence in CRC patients, we designed this study in two phases: a discovery phase for the selection of candidate miRNAs, and a validation phase with two independent CRC clinical patient cohorts to assess the efficacy of the candidate miRNA as a CRC relapse marker.

In the discovery phase, two patient cohorts were analyzed to identify potential miRNA candidates. The first cohort consisted of 100 stage III CRC patients which included 50 patients with and 50 patients without CRC recurrence within 3 years after treatment with FOLFOX. This patient cohort was enrolled at Mayo Clinic, MN, USA, as a part of a randomized phase III clinical trial (North Central Cancer Treatment Group [NCCTG] N0147). NCCTG is now part of the Alliance for Clinical Trials in Oncology. All patients enrolled in this study were treated with either FOLFOX alone or in combination with cetuximab as adjuvant treatment^49^. The clinical specimens used in this study were limited to the patients treated with FOLFOX alone. Each participant signed an IRB-approved, protocol-specific informed consent in accordance with federal and institutional guidelines.

Data derived from the TCGA were used as the second patient cohort. The miRNA-Seq expression data and the CRC patients’ corresponding clinical information were downloaded from the TCGA data portal on 16 August, 2015. The dataset included 603 level 3 colon adenocarcinoma miRNA data sets, of which we identified 147 stage II/III CRC patients with fully-annotated detailed clinical information and miRNA values were identified. Among these 147 patients, 26 patients had experienced tumor recurrence, while 121 did not. Data points were summarized either as a mean with 95% confidence intervals on a log scale.

Following the selection of candidate miRNAs from these two discovery patient cohorts, we evaluated the expression of these miRNAs in a small set of stage III formalin-fixed, paraffin-embedded (FFPE) tumors from stage III CRC patients (12 with and 12 without-recurrence) from Mie University Medical Hospital, Mie, Japan. In the validation phase, 111 stage II/III CRC FFPE tissues (31 with recurrence and 80 without relapse) from Mie University Medical Hospital and 139 stage I-III CRC FFPE tissues (38 with and 101 without recurrence) from Okayama University Medical Hospital, Okayama, Japan were analyzed. Furthermore, serum specimens from 53 stage I-IV CRC patients (stage I; 12, stage II; 12, stage III; 17, stage IV; 12) from Mie University Medical Hospital were also examined. In addition, we analyzed an independent set of 30 primary CRC tissues and matched liver metastasis tissues from the Toho University Medical Hospital, Japan.

All experimental methods were carried out in accordance with relevant guidelines and regulations. Written informed consent was obtained from all patients to use their tissue specimens for research purposes, and the study was approved by the institutional review boards of Baylor Research Institute, Dallas, USA; Mie University, Japan; and Mayo Clinic, Rochester, USA.

### MiRNA expression microarray

MiRNA microarray expression profiling analysis included interrogation of 2221 miRNAs, using the Affymetrix GeneChip miRNA 2.0 Arrays (Santa Clara, CA, USA). Each sample was labelled using the Genisphere FlashTag Biotin HSR kit (Hatfield, PA, USA). Briefly, 1 mg of total RNA was incubated with ATP and poly-A-polymerase to add a 3′-polyA tail. Thereafter, a ligation reaction was performed to covalently attach a multiple-biotin molecule containing a 3′-DNA dendrimer to the miRNA. Labeled samples were washed and stained in an Affymetrix Fluidics Station 450, scanned using an Affymetrix 3000 7 G scanner. The expression changes between CRC cases with and without recurrence were analyzed.

### Cell lines

Human colorectal cancer cell lines SW480, SW620, LoVo, RKO, Caco-2, HCT-116 and HT-29 were purchased from the American Type Culture Collection (ATCC, Rockville, Maryland). These cell lines were grown in Iscove’s modified Dulbecco’s medium (Invitrogen) with 10% fetal bovine serum and 1% penicillin and streptomycin and maintained at 37 °C in a humidified incubator (5% CO_2_). All cell lines were routinely tested and authenticated using a panel of genetic and epigenetic markers.

### RNA extraction

MiRNeasy FFPE Kit (Qiagen, Valencia, CA) was used to extract total RNA enriched for small RNAs according to manufacturer’s instructions. Tissue sections were carefully micro-dissected to enrich for neoplastic cells. RNA was thereafter extracted using automated QIAcube system (Qiagen) and RNA was eluted to a final volume of 30 μL in RNase-free water. Similarly, miRNeasy Serum/Plasma Kit was used to extract RNA from 200 μl serum using QIAcube system per manufacturer’s instructions. 25 fmol of synthetic *C. elegans* miRNA (cel-miR-39, Qiagen) was used as a control for the qRT-PCR analysis as a normalizer.

### qRT-PCR analysis

For miRNA-based qRT-PCR analysis, 2 μL of RNA was reverse-transcribed using the TaqMan MicroRNA Reverse Transcription Kit (Applied Biosystems, Foster City, CA) in a total reaction volume of 10 μL. qRT-PCR was performed with MicroRNA Assay Kits and TaqMan Universal Master Mix II, no UNG (Applied Biosystems) using QuantStudio 6 Flex Real-Time PCR System (Applied Biosystems). All results were expressed as 2^−ΔΔCt^. The expression of tissue derived miRNAs was normalized against U6 (Ambion, Austin, TX), while serum derived miRNAs were normalized to cel-miR-39 (Qiagen).

For mRNA qRT-PCR analysis, total RNAs were reverse transcribed to cDNA using the Advantage RT PCR Kit (Clontech Laboratories, Inc., Mountain View, CA, USA). qRT-PCR was performed with Power SYBR Green Master Mix (Life Technology, USA) using QuantStudio 6 Flex Real-Time PCR System (Applied Biosystems). All results were expressed as 2^−ΔΔCt^ and normalized to glyceraldehyde-3-phosphate dehydrogenase (GAPDH) expression. All primer sequences are described in the Supplemental Table 6.

### MiR-139-5p Overexpression

Based upon their low endogenous expression levels for miR-139-59, two CRC cell lines (RKO and Caco-2 cells) were selected for transfection experiments, and 5 × 10^5^ cells were transfected with mirVana miRNA Mimic (micro-RNA-139-5p) or Negative Control (Life Technologies Corporation) at a final concentration of 5 nM using Lipofectamine RNAiMAX (Life Technologies Corporation) following the manufacturer’s protocol.

### Analysis of protein expression

The total protein extracted from each cell line was collected using RIPA lysis buffer. A total of 30 μg protein was loaded per sample on 10% polyacrylamide-SDS gels, which subsequently was transferred onto nitrocellulose membranes. The membranes were then blocked in 5% fat-free milk and incubated with the following primary antibodies overnight at 4 °C : goat anti-ZEB1 (1:1000 dilution; Santa Cruz Biotechnology, Santa Cruz, CA, USA), mouse anti-ZEB2 (1:1000 dilution; Santa Cruz Biotechnology, Santa Cruz, CA, USA), mouse anti-E-cadherin (1:1000 dilution; BD Biosciences, Bedfold, Massachusetts, USA), monoclonal mouse anti-β-actin (1: 5000 dilution; Sigma-Aldrich, St. Louis, MO). Membranes were thereafter incubated with secondary horseradish peroxidase-conjugated (HRP) conjugated anti-mouse antibody (1:3000 dilution; Santa Cruz Biotechnology) or anti-goat antibody (1:3000 dilution; Santa Cruz Biotechnology) at room temperature for 1 hour. The proteins were detected using the Syngene G:Box (Syngene, Cambridge, UK), a chemiluminescence system, according to the manufacturer’s instructions.

### Mouse CRC peritoneal metastasis model

Twenty two male athymic nude mice were obtained from Harlan Laboratories (Houston, TX, USA) at 5 weeks of age and kept under controlled conditions (12-hour light and dark cycles). To establish a mouse peritoneal metastasis model, 3 × 10^6^ Caco-2 cells transfected with miR-139-5p mimic or the negative control were injected intraperitoneally into mice. Mice were sacrificed 60 days post-injection and the number of nodules in the mesentery and peritoneal walls were evaluated. The animal protocol was approved by the Institutional Animal Care and Use Committee of the Baylor Research Institute and all experiments were conducted strictly in accordance to the National Institute of Health Guide for the Care and Use of Laboratory Animals (8^th^ Edition Institute for Laboratory Animal Research).

### Statistical analysis

All statistical analyses were performed using Medcalc Statistical Software v.12.7.7. (Medcalc Software, Ostend, Belgium). Differences between groups were estimated by Wilcoxon’s signed rank test, the Chi square test, Mann-Whitney U test, and One-way ANOVA analysis, as appropriate. F-tests were used to assess the equality of variance for comparable groups. Pearson’s correlation coefficient (r) was used to evaluate the linear relationship between two variables. For time-to-event analysis, survival estimates were calculated using the Kaplan-Meier analysis, and groups were compared with the log-rank test. ROC curves were established to discriminate the patients with or without recurrence, and the Youden’s index was used to determine the optimal cutoff thresholds for miR-139-5p expression to predict the recurrence. Recurrence-free survival was measured from the date the patient underwent curative surgery to the date of disease relapse.

ROC curve analysis was also used to evaluate whether serum miR-139-5p expression levels could distinguish patients with or without CRC recurrence. The Cox’s proportional hazards models were used to estimated hazard ratios (HRs) for CRC relapse. Assumption of proportionality was confirmed for the Cox proportional hazards analysis by generating Kaplan-Meier survival curves (e.g. high vs. low expression groups) and by ensuring the two curves did not intersect each other. Multivariate logistic regression models were utilized to predict factors influencing recurrence in CRC patients. Forced-entry regression was employed to include these variables in all multivariable equations in order to analyze whether each of the predictors affected the outcome after adjusting for known confounders. All P values were 2-sided, and those less than 0.05 were considered statistically significant.

## Additional Information

**How to cite this article:** Miyoshi, J. *et al*. MiR-139-5p as a novel serum biomarker for recurrence and metastasis in colorectal cancer. *Sci. Rep.* **7**, 43393; doi: 10.1038/srep43393 (2017).

**Publisher's note:** Springer Nature remains neutral with regard to jurisdictional claims in published maps and institutional affiliations.

## Supplementary Material

Supplementary Data

## Figures and Tables

**Table 1:** Univariate and Multivariate analysis in validation cohorts.

Cohort	Variable	Univariate analysis			Multivariate analysis		
		HR	95% CI	*p* value	HR	95% CI	*p value*
Cohort 1	Age (>median vs. <median)	1.00	0.49–2.03	0.996			
	Gender (Female vs. Male)	1.15	0.56–2.36	0.696			
	T stage (3–4 vs. 1–2)	5.89	1.40–24.7	0.015	**4.65**	**1.04–20.6**	**0.043**
	Lymph node metastasis (presence vs. absence)	3.42	1.62–7.20	0.001	**3.25**	**1.50–7.04**	**0.002**
	CEA (>7.75 vs. <7.75)	1.22	0.59–2.50	0.585			
	Tumor Location (rectum vs. colon)	1.39	0.68–2.82	0.360			
	Histology (undiff vs. diff)	1.23	0.43–3.55	0.690			
	Venous invasion (presence vs. absence)	1.94	0.91–4.11	0.082			
	Lymphatic invasion (presence vs. absence)	5.71	0.77–42.0	0.086			
	miR-139-5p expression (high vs. low)	4.61	2.09–10.2	<0.001	**2.63**	**1.13–6.11**	**0.024**
Cohort 2	Age (>median vs. <median)	1.05	0.56–1.98	0.859			
	Gender (Male vs. Female)	1.21	0.63–2.33	0.563			
	T stage (3-4 vs. 1-2)	11.0	1.51–80.3	0.017	**7.49**	**1.00–55.7**	**0.049**
	Lymph node metastasis (presence vs. absence)	2.06	1.09–3.89	0.025	**2.03**	**1.06–3.87**	**0.030**
	Tumor Location (rectal vs. colon)	1.69	0.89–3.19	0.102			
	Histology (poor and mod vs. well)	1.31	0.62–2.77	0.471			
	Venous invasion (presence vs. absence)	1.38	0.60–3.16	0.439			
	Lymphatic invasion (presence vs. absence)	3.44	0.47–25.1	0.222			
	miR-139-5p expression (high vs. low)	2.78	1.45–5.32	0.002	**2.30**	**1.19–4.46**	**0.012**

Bold: difference significant (p < 0.05). Abbreviations: Well, well differentiated; mod, moderately differentiated; *P* value were calculated by Cox proportion hazard model. HR: hazard ratio.
