# Supplementary material for: MiR-139-5p as a novel serum biomarker for recurrence and metastasis in colorectal cancer
Source: Sci Rep. 2017 Mar 6;7:43393. doi: 10.1038/srep43393 (PMC5338356; doi:10.1038/srep43393)
Supplement: Supplementary Data [file srep43393-s1.pdf]

MiR-139-5p as a novel serum and tissue biomarker for recurrence and metastasis of colorectal cancer.

Jinsei Miyoshi[1,2], Shusuke Toden[1], Kazuhiro Yoshida[1,3], Yuji Toiyama[4], Steven R Alberts[5], , Masato Kusunoki[4], Frank A. Sinicrope[5] and Ajay Goel[1]

[1] Center for Gastrointestinal Research; Center for Translational Genomics and Oncology, Baylor Scott & White Research Institute and Sammons Cancer Center, Baylor University Medical Center, Dallas, Texas, USA.

[2] Department of Gastroenterology and Oncology, Institutes of Biomedical Sciences, Tokushima University Graduate School, Tokushima, Japan.

[3] Department of Gastroenterological Surgery, Okayama University Graduate School of Medicine, Dentistry and Pharmaceutical Sciences, Okayama, Japan.

[4] Department of Gastrointestinal and Pediatric Surgery, Division of Reparative Medicine, Institute of Life Sciences, Graduate School of Medicine, Mie University, Mie, Japan.

[5] Division of Medical Oncology, Mayo Clinic and Mayo Cancer Center, Rochester, Minnesota, USA.

SUPPLEMENTAL TABLES

Supplemental Table 1: Characteristics of Mayo Clinic cohort.

**Clinical characteristic of patients**		**N**	**(%)**
Age	Median age (range)	59	(25-81)
Gender, n (%)	Female	46	(47.4)
	Male	51	(52.6)
Location, n (%)	Right side	49	(50.5)
	Left side	48	(49.5)
TNM Stage, n (%)	I	0	(0)
	II	0	(0)
	III	97	(100)
	IV	0	(0)
Grade, n (%)	Low (grade 1-2)	68	(70.1)
	High (grade 3-4)	29	(29.9)
MMR status, n (%)	Deficient	10	(10.3)
	Proficient	87	(89.7)

Abbreviations: Right side; cecum, ascending and transvers colon, Left side; descending and sigmoid colon and rectum, TMN; tumor, node, metastasis, MMR: mismatch repair.

Supplemental Table 2: Characteristics of TCGA cohort.

Clinical characteristic of patients		N	(%)
Age	Median age (range)	65	(37-90)
Gender	Female	72	(48.9)
	Male	75	(51.0)
Location	Colon	100	(68.0)
	Rectum	46	(31.2)
TNM Stage	I	0	(0)
	II	42	(28.5)
	III	105	(71.4)
	IV	0	(0)
Tumor stage	T1 and T2	8	(7.61)
	T3 and T4	139	(92.3)
Mucinous	Yes	19	(12.9)
	No	128	(87.0)

Abbreviations: TMN: tumor, node, metastasis.

Supplemental Table 3: Clinicopathological variables and miR-139-5p expression in Cohort 1.

Variables		N	miR-139-5p expression		*p* value
			Hi (N=51)	Low (N=60)	
Age (y)	>67 (median)	54	25	29	0.942
	≤67	57	26	31	
Sex	Female	43	23	20	0.206
	Male	68	28	40	
Cancer type	Rectum	46	23	23	0.472
	Colon	65	28	37	
Histological type	Undifferentiated	10	5	5	0.788
	Differentiated	101	46	55	
Pathological T stage	T3/T4	81	47	34	<0.001*
	T1/T2	30	4	26	
Venous invasion	+	48	25	23	0.259
	-	63	26	37	
Lymphatic invasion	+	90	40	50	0.513
	-	21	11	10	
TNM Stage	III	51	27	24	0.174
	II	60	24	36	

* p <0.05

Supplemental Table 4: Clinicopathological variables and miR-139-5p expression in Cohort 2.

Variables		N	miR-139-5p expression		*p* value
			Hi (N=29)	Low (N=110)	
Age (y)	>66 (median)	65	12	53	0.515
	≤66	74	17	57	
Sex	Female	56	9	47	0.407
	Male	83	20	63	
Cancer type	Rectum	57	17	40	0.030*
	Colon	82	12	70	
Histological type	Undifferentiated	36	5	31	0.233
	Differentiated	103	24	79	
Pathological T stage	T3/T4	112	29	83	0.003*
	T1/T2	27	0	27	
Venous invasion	+	101	19	82	0.763
	-	33	7	26	
Lymphatic invasion	+	130	25	105	0.072
	-	9	4	5	
TNM Stage	III	60	13	47	0.800
	I/II	79	16	63	

* $p < 0.05$

Supplemental Table 5: Clinicopathological variables and miR-139-5p expression in Cohort 3.

**Variables**		**N**	**miR-139-5p expression** **Hi (N=15)**	**Low (N=26)**	***p* value**
Age (y)	>67 (median)	20	8	12	0.661
	≤67	21	7	14	
Sex	Female	17	7	10	0.611
	Male	24	8	16	
Cancer type	Rectum	15	6	9	0.733
	Colon	26	9	17	
Histological type	Undifferentiated	5	1	4	0.417
	Differentiated	36	14	22	
Pathological T stage	T3/T4	27	12	15	0.151
	T1/T2	14	3	11	
Venous invasion	+	16	9	7	0.038*
	-	25	6	19	
Lymphatic invasion	+	31	13	18	0.216
	-	10	2	8	
TNM Stage	III	18	10	8	0.027*
	I/II	23	5	18	

* $p < 0.05$

Supplemental Table 6: Primer for RT-PCR amplification.

ZEB1-RT-F	5’-GATGATGAATGCGAGTCAGATGC-3’
ZEB1-RT-R	5’-ACAGCAGTGTCTTGTTGTTGT-3’
ZEB2-RT-F	5’-GGAGACGAGTCCAGCTAGTGT-3’
ZEB2-RT-R	5’-CCACTCCACCCTCCCTTATTTC-3’
E-Cadherin-RT-F	5’-ATTTTTCCCTCGACACCCGAT-3’
E-Cadherin-RT-R	5’-TCCCAGGCGTAGACCAAGA-3’
GAPDH-RT-F	5’-CTGCACCACCAACTGCTTAG-3’
GAPDH-RT-R	5’-GTCTTCTGGGTGGCAGTGAT-3’

SUPPLEMENTAL DATA

Supplemental Data 1: Summary statistics from analyses comparing recurrence CRC patients (n=50) to non-recurrence CRC patients (n=47) in the Mayo clinic cohort.

SUPPLEMENTAL FIGURE LEGENDS

Supplemental Figure 1: MiR-139-5p expression in primary CRC with subsequent liver metastasis, lung metastasis and other metastasis. Recurrence sites in cohort 1: liver metastasis n=11, lung metastasis n=12, other metastasis n=8.

Supplemental Figure 2: MiR-139-5p expression levels between microsatellite stable (MSS) and microsatellite instable (MSI) tumors in stage III CRC. Microsatellite instability (MSI) status: MSS n=84, MSI n=20.

Supplemental Figure 3: *The expression levels of miR-139-5p in CRC cell lines.* 7 CRC cell lines: SW480, SW620, Caco-2, LoVo, RKO, HCT-116, HT-29.

Supplemental Figure 1

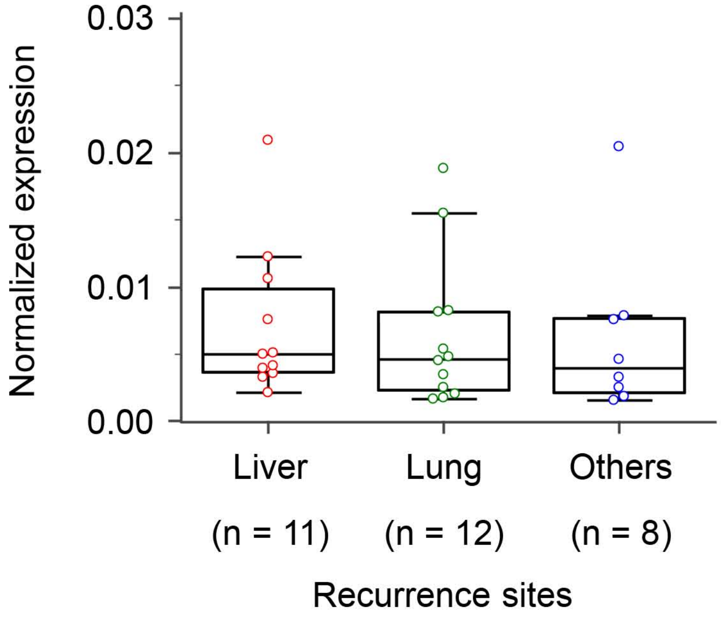

Supplemental Figure 2

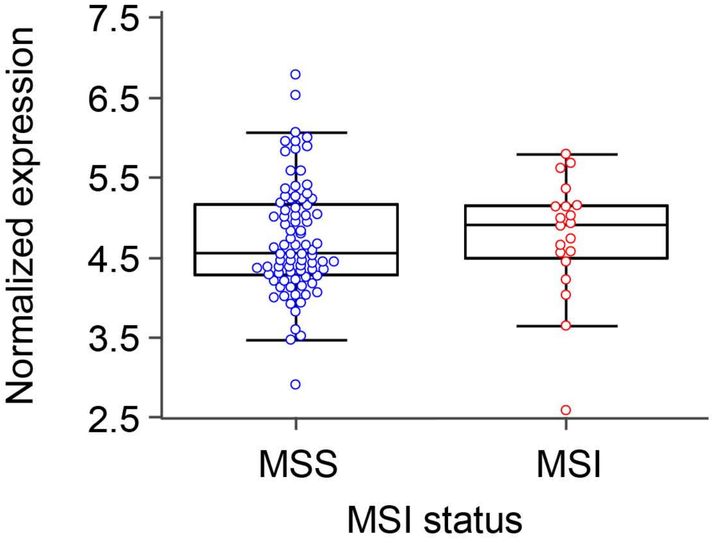

Supplemental Figure 3

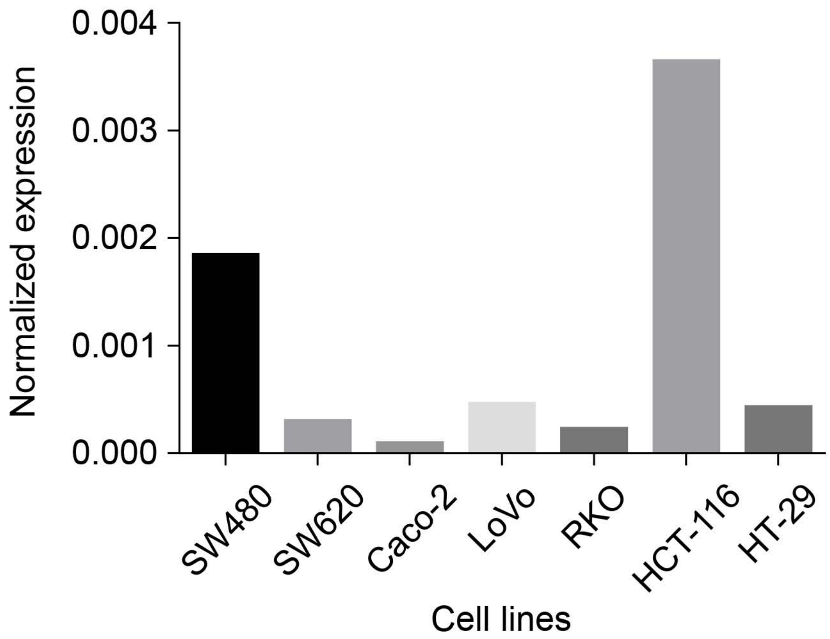

Supplemental Data 1. Summary statistics from analyses comparing recurrence CRC patients (n=50) to non-recurrence CRC patients (n=47) in the Mayo clinic cohort

miRNA ID	log2FC	p-value	q-value	Mean in recurrences	Mean in non-recurrences	Overall Mean	Overall SD
hp_hsa-mir-518e_x_st	-0.090	3.22E-05	0.050	5.57	5.66	5.61	0.112
hsa-miR-320e_st	0.350	4.48E-05	0.050	8.07	7.72	7.90	0.440
hp_hsa-mir-125a_st	0.081	4.25E-04	0.315	5.88	5.80	5.84	0.116
hsa-miR-433_st	0.148	7.22E-04	0.380	5.70	5.56	5.63	0.221
hp_hsa-mir-487b_x_st	0.072	9.46E-04	0.380	5.66	5.58	5.62	0.110
hp_hsa-mir-497_st	0.083	1.11E-03	0.380	5.88	5.80	5.84	0.129
hsa-miR-382_st	0.301	1.32E-03	0.380	7.01	6.71	6.87	0.473
hsa-miR-1306_st	0.046	1.65E-03	0.380	5.45	5.40	5.43	0.073
hp_hsa-mir-30a_st	0.035	2.08E-03	0.380	5.41	5.37	5.39	0.057
hsa-miR-487b_st	0.247	2.29E-03	0.380	6.60	6.36	6.48	0.408
hsa-miR-181c_st	0.210	2.38E-03	0.380	6.19	5.98	6.08	0.348
hsa-miR-152_st	0.335	2.48E-03	0.380	8.14	7.81	7.98	0.558
hp_hsa-mir-432_st	0.039	2.66E-03	0.380	5.52	5.48	5.50	0.065
hp_hsa-mir-376c_x_st	0.040	2.86E-03	0.380	5.41	5.37	5.39	0.067
hp_hsa-mir-4273_st	0.031	2.90E-03	0.380	5.35	5.32	5.33	0.052
hsa-miR-4283_st	0.041	2.93E-03	0.380	5.46	5.42	5.44	0.069
hsa-miR-345_st	-0.284	3.02E-03	0.380	7.51	7.79	7.64	0.483
hsa-miR-425_st	-0.348	3.08E-03	0.380	9.56	9.91	9.73	0.593
hsa-miR-125a-5p_st	0.446	3.41E-03	0.399	8.71	8.27	8.50	0.767
hsa-miR-493_st	0.100	3.83E-03	0.408	5.77	5.67	5.72	0.174
hsa-miR-134_st	0.228	3.89E-03	0.408	6.87	6.64	6.75	0.398
hsa-miR-409-3p_st	0.289	4.04E-03	0.408	7.10	6.81	6.96	0.506
hsa-miR-10b_st	0.343	4.61E-03	0.445	7.43	7.09	7.26	0.609
hp_hsa-mir-197_st	0.064	4.82E-03	0.446	5.99	5.93	5.96	0.114
hp_hsa-mir-3160-1_s_st	-0.050	5.03E-03	0.447	5.53	5.58	5.55	0.089
hsa-miR-320c_st	0.241	5.61E-03	0.468	11.96	11.72	11.85	0.437
hsa-miR-432_st	0.265	5.69E-03	0.468	6.78	6.52	6.65	0.481
hsa-miR-154_st	0.100	6.60E-03	0.518	5.68	5.58	5.63	0.185
hsa-miR-1197_st	-0.032	6.76E-03	0.518	5.37	5.40	5.38	0.058
hp_hsa-mir-487b_st	0.075	8.31E-03	0.574	5.83	5.75	5.79	0.143
hsa-miR-181a_st	0.283	8.71E-03	0.574	10.11	9.83	9.98	0.540
hp_hsa-mir-23a_x_st	0.088	8.86E-03	0.574	6.92	6.83	6.88	0.168
hsa-miR-320d_st	0.237	8.94E-03	0.574	10.29	10.05	10.18	0.454
hp_hsa-mir-622_st	-0.099	9.09E-03	0.574	5.64	5.74	5.69	0.190
hp_hsa-mir-4269_st	0.043	9.71E-03	0.574	5.49	5.45	5.47	0.083
hp_hsa-mir-421_st	-0.062	1.04E-02	0.574	5.59	5.65	5.62	0.121
hsa-miR-29c-star_st	0.090	1.15E-02	0.574	5.62	5.53	5.58	0.178
hsa-miR-191_st	-0.200	1.18E-02	0.574	12.15	12.35	12.24	0.397
hsa-miR-485-5p_st	0.090	1.20E-02	0.574	5.63	5.54	5.59	0.180
hp_hsa-mir-1184-1_s_st	0.037	1.20E-02	0.574	5.56	5.53	5.54	0.073
hsa-miR-744_st	-0.196	1.22E-02	0.574	8.09	8.28	8.18	0.391
hsa-miR-4328_st	0.027	1.23E-02	0.574	5.34	5.32	5.33	0.053

hsa-miR-502-5p_st	-0.045	1.23E-02	0.574	5.47	5.51	5.49	0.090
hsa-miR-3146_st	-0.032	1.33E-02	0.574	5.33	5.36	5.35	0.064
hsa-miR-125b_st	0.415	1.33E-02	0.574	11.03	10.62	10.83	0.838
hsa-miR-362-5p_st	-0.254	1.34E-02	0.574	7.74	7.99	7.86	0.515
hp_hsa-mir-3118-3_x_st	-0.022	1.42E-02	0.574	5.34	5.36	5.35	0.045
hsa-miR-127-3p_st	0.284	1.45E-02	0.574	8.45	8.17	8.31	0.580
hp_hsa-mir-26a-2_x_st	0.042	1.48E-02	0.574	5.87	5.83	5.85	0.085
hp_hsa-mir-1276_st	0.033	1.49E-02	0.574	5.45	5.42	5.43	0.067
hp_hsa-mir-656_st	0.035	1.50E-02	0.574	5.53	5.50	5.51	0.071
hsa-miR-29c_st	0.098	1.52E-02	0.574	5.71	5.61	5.67	0.202
hsa-miR-99b_st	0.331	1.53E-02	0.574	10.19	9.86	10.03	0.681
hp_hsa-mir-454_st	0.030	1.54E-02	0.574	5.45	5.42	5.44	0.062
hp_hsa-mir-648_st	0.031	1.63E-02	0.574	5.44	5.41	5.43	0.064
hp_hsa-mir-99b_st	0.099	1.68E-02	0.574	6.23	6.13	6.18	0.207
hsa-let-7e_st	0.320	1.76E-02	0.574	11.33	11.01	11.17	0.675
hp_hsa-mir-190b_st	-0.033	1.77E-02	0.574	5.49	5.53	5.51	0.070
hsa-miR-99b-star_st	0.195	1.77E-02	0.574	6.62	6.43	6.53	0.410
hsa-miR-626_st	0.027	1.78E-02	0.574	5.35	5.32	5.34	0.057
hsa-let-7i-star_st	0.044	1.84E-02	0.574	5.57	5.53	5.55	0.093
hsa-miR-18b-star_st	-0.029	1.84E-02	0.574	5.35	5.38	5.37	0.061
hp_hsa-mir-425_st	-0.082	1.85E-02	0.574	6.42	6.50	6.46	0.174
hsa-miR-875-3p_st	0.036	1.90E-02	0.574	5.41	5.38	5.40	0.076
hsa-miR-609_st	-0.026	1.93E-02	0.574	5.27	5.30	5.28	0.055
hp_hsa-let-7c_x_st	0.036	1.93E-02	0.574	5.45	5.41	5.43	0.077
hsa-miR-500_st	-0.202	2.02E-02	0.574	7.33	7.53	7.43	0.433
hp_hsa-mir-663_x_st	-0.264	2.07E-02	0.574	7.73	7.99	7.86	0.570
hp_hsa-mir-382_st	0.032	2.11E-02	0.574	5.49	5.46	5.47	0.069
hp_hsa-mir-194-2_x_st	-0.109	2.13E-02	0.574	7.48	7.59	7.54	0.235
hsa-let-7f_st	0.273	2.13E-02	0.574	8.85	8.58	8.72	0.592
hsa-miR-769-5p_st	-0.123	2.13E-02	0.574	6.10	6.23	6.16	0.267
hp_hsa-mir-4258_st	-0.030	2.14E-02	0.574	5.33	5.36	5.35	0.064
hp_hsa-mir-194-2_st	-0.074	2.15E-02	0.574	6.54	6.62	6.58	0.161
hsa-miR-139-3p_st	0.069	2.16E-02	0.574	5.66	5.59	5.63	0.149
hsa-miR-758_st	0.035	2.18E-02	0.574	5.37	5.33	5.35	0.077
hp_hsa-mir-1302-7_x_st	-0.042	2.22E-02	0.574	5.47	5.51	5.49	0.090
hsa-miR-378_st	-0.287	2.23E-02	0.574	9.55	9.84	9.69	0.626
hsa-miR-10b-star_st	0.077	2.24E-02	0.574	5.62	5.55	5.59	0.167
hsa-miR-139-5p_st	0.206	2.26E-02	0.574	6.02	5.81	5.92	0.451
hp_hsa-mir-4293_st	-0.024	2.27E-02	0.574	5.35	5.37	5.36	0.052
hsa-miR-194-star_st	-0.245	2.28E-02	0.574	7.12	7.36	7.24	0.538
hp_hsa-mir-28_st	0.052	2.28E-02	0.574	5.99	5.94	5.97	0.113
hsa-miR-28-3p_st	0.206	2.28E-02	0.574	8.50	8.30	8.40	0.452
hp_hsa-mir-568_st	0.026	2.32E-02	0.574	5.37	5.34	5.35	0.056
hsa-miR-193b-star_st	0.155	2.34E-02	0.574	6.43	6.27	6.35	0.342
hp_hsa-mir-625_x_st	-0.061	2.39E-02	0.574	5.77	5.83	5.80	0.135
hp_hsa-mir-214_s_st	0.130	2.49E-02	0.574	6.98	6.85	6.92	0.290
hp_hsa-mir-1285-1_x_st	-0.031	2.50E-02	0.574	5.47	5.50	5.48	0.069

hp_hsa-mir-124-3_s_st	0.058	2.52E-02	0.574	5.52	5.46	5.49	0.130
hsa-miR-421_st	-0.232	2.53E-02	0.574	6.67	6.91	6.79	0.517
hp_hsa-mir-1308_st	-0.132	2.54E-02	0.574	7.85	7.98	7.91	0.295
hp_hsa-mir-4256_st	-0.041	2.54E-02	0.574	5.56	5.60	5.58	0.090
hsa-miR-2116_st	0.039	2.55E-02	0.574	5.41	5.37	5.39	0.087
hsa-miR-1308_st	-0.300	2.59E-02	0.574	11.76	12.05	11.90	0.672
hp_hsa-mir-3148_st	-0.029	2.61E-02	0.574	5.35	5.38	5.37	0.065
hsa-miR-338-5p_st	0.076	2.62E-02	0.574	5.67	5.60	5.64	0.169
hsa-miR-944_st	-0.021	2.62E-02	0.574	5.32	5.34	5.33	0.047
hp_hsa-mir-624_st	-0.023	2.65E-02	0.574	5.31	5.34	5.33	0.052
hsa-miR-504_st	0.037	2.65E-02	0.574	5.43	5.40	5.42	0.082
hsa-miR-299-5p_st	0.041	2.67E-02	0.574	5.52	5.48	5.50	0.091
hsa-miR-486-5p_st	0.330	2.69E-02	0.574	6.65	6.32	6.49	0.743
hsa-miR-802_st	-0.025	2.71E-02	0.574	5.27	5.29	5.28	0.055
hsa-miR-1288_st	0.022	2.71E-02	0.574	5.32	5.30	5.31	0.049
hsa-miR-1299_st	0.117	2.72E-02	0.574	5.54	5.42	5.48	0.264
hsa-miR-411_st	0.056	2.87E-02	0.592	5.62	5.56	5.59	0.127
hsa-miR-3166_st	-0.030	2.88E-02	0.592	5.40	5.43	5.41	0.068
hp_hsa-mir-199a-1_x_st	0.104	2.90E-02	0.592	6.84	6.73	6.79	0.238
hsa-miR-320b_st	0.201	2.96E-02	0.592	12.23	12.03	12.13	0.460
hp_hsa-mir-409_st	0.053	2.96E-02	0.592	5.69	5.64	5.66	0.122
hsa-miR-448_st	-0.026	2.96E-02	0.592	5.29	5.32	5.31	0.058
hp_hsa-mir-125b-2_x_st	0.061	2.98E-02	0.592	5.83	5.77	5.80	0.140
hsa-miR-100_st	0.331	3.07E-02	0.604	9.56	9.23	9.40	0.763
hsa-miR-409-5p_st	0.073	3.11E-02	0.606	5.68	5.60	5.64	0.169
hsa-miR-31-star_st	0.135	3.15E-02	0.606	5.51	5.37	5.44	0.313
hp_hsa-mir-3146_st	-0.021	3.18E-02	0.606	5.33	5.35	5.34	0.049
hsa-miR-25-star_st	-0.119	3.19E-02	0.606	6.34	6.46	6.39	0.277
hsa-miR-126_st	0.261	3.27E-02	0.613	9.81	9.55	9.68	0.608
hsa-miR-93_st	-0.229	3.28E-02	0.613	11.24	11.47	11.35	0.535
hp_hsa-mir-2278_st	-0.032	3.38E-02	0.625	5.54	5.57	5.55	0.074
hp_hsa-mir-26a-1_x_st	0.051	3.46E-02	0.632	6.56	6.51	6.54	0.120
hp_hsa-mir-106b_st	-0.070	3.57E-02	0.632	6.28	6.35	6.32	0.165
hp_hsa-mir-652_st	-0.055	3.64E-02	0.632	6.17	6.23	6.20	0.130
hsa-miR-378-star_st	-0.164	3.66E-02	0.632	6.40	6.57	6.48	0.390
hp_hsa-mir-2052_st	0.019	3.67E-02	0.632	5.33	5.31	5.32	0.044
hp_hsa-mir-520a_x_st	0.021	3.68E-02	0.632	5.33	5.31	5.32	0.049
hp_hsa-mir-302a_st	-0.029	3.68E-02	0.632	5.33	5.36	5.35	0.067
hp_hsa-mir-4324_st	0.030	3.69E-02	0.632	5.48	5.45	5.46	0.072
hsa-miR-3192_st	0.036	3.73E-02	0.632	5.44	5.40	5.42	0.086
hp_hsa-mir-517a_x_st	-0.041	3.74E-02	0.632	5.48	5.52	5.50	0.099
hsa-miR-320a_st	0.191	3.80E-02	0.632	12.46	12.26	12.36	0.458
hp_hsa-mir-381_st	0.026	3.81E-02	0.632	5.42	5.39	5.40	0.061
hp_hsa-mir-31_st	0.202	3.82E-02	0.632	6.43	6.23	6.33	0.484
hp_hsa-mir-433_st	0.025	3.84E-02	0.632	5.40	5.38	5.39	0.060
hsa-miR-3196_st	-0.255	3.86E-02	0.632	12.97	13.23	13.10	0.615
hp_hsa-mir-802_st	0.021	3.87E-02	0.632	5.34	5.32	5.33	0.050

hp_hsa-mir-567_st	-0.026	4.01E-02	0.635	5.47	5.49	5.48	0.063
hsa-miR-132_st	0.229	4.02E-02	0.635	7.80	7.57	7.69	0.557
hsa-miR-551a_st	-0.030	4.04E-02	0.635	5.42	5.45	5.43	0.071
hsa-miR-329_st	0.031	4.05E-02	0.635	5.39	5.36	5.38	0.074
hsa-miR-370_st	0.082	4.07E-02	0.635	6.35	6.26	6.31	0.199
hsa-miR-625_st	-0.261	4.08E-02	0.635	7.20	7.46	7.33	0.636
hsa-miR-199a-3p_st	0.319	4.09E-02	0.635	9.70	9.39	9.55	0.776
hp_hsa-mir-500_x_st	-0.076	4.13E-02	0.636	6.38	6.45	6.41	0.184
hsa-miR-195-star_st	0.042	4.18E-02	0.638	5.58	5.54	5.56	0.103
hp_hsa-mir-522_x_st	-0.062	4.20E-02	0.638	5.55	5.61	5.58	0.151
hp_hsa-mir-3201_x_st	-0.040	4.31E-02	0.644	5.45	5.49	5.47	0.098
hsa-miR-376c_st	0.067	4.34E-02	0.644	5.56	5.50	5.53	0.166
hsa-miR-455-3p_st	0.214	4.39E-02	0.644	8.63	8.42	8.53	0.529
hsa-miR-199b-3p_st	0.320	4.41E-02	0.644	9.71	9.39	9.55	0.791
hsa-miR-26b_st	0.097	4.51E-02	0.644	6.01	5.91	5.96	0.241
hp_hsa-mir-3133_st	-0.022	4.53E-02	0.644	5.35	5.37	5.36	0.054
hsa-miR-181b_st	0.227	4.54E-02	0.644	9.07	8.84	8.96	0.565
hsa-miR-1278_st	-0.041	4.59E-02	0.644	5.47	5.52	5.49	0.103
hp_hsa-mir-1826_st	-0.106	4.66E-02	0.644	7.49	7.60	7.54	0.266
hsa-miR-532-5p_st	-0.208	4.70E-02	0.644	8.69	8.90	8.79	0.520
hp_hsa-mir-758_x_st	0.025	4.72E-02	0.644	5.44	5.42	5.43	0.063
hsa-miR-516a-3p_st	0.024	4.72E-02	0.644	5.30	5.28	5.29	0.059
hsa-miR-148b-star_st	-0.023	4.76E-02	0.644	5.32	5.34	5.33	0.056
hp_hsa-mir-152_st	0.034	4.80E-02	0.644	5.76	5.73	5.74	0.084
hsa-miR-30a-star_st	0.126	4.89E-02	0.644	5.86	5.74	5.80	0.319
hsa-miR-1283_st	0.019	4.89E-02	0.644	5.29	5.27	5.28	0.048
hp_hsa-mir-203_st	-0.047	4.96E-02	0.644	5.86	5.91	5.89	0.119
hsa-miR-184_st	0.125	4.98E-02	0.644	5.60	5.48	5.54	0.316
hsa-miR-3143_st	0.026	5.00E-02	0.644	5.38	5.35	5.37	0.066
hp_hsa-mir-378c_st	0.032	5.11E-02	0.644	5.64	5.61	5.62	0.082
hsa-miR-199a-5p_st	0.255	5.12E-02	0.644	9.84	9.58	9.71	0.650
hsa-miR-29a-star_st	-0.018	5.20E-02	0.644	5.28	5.30	5.29	0.045
hsa-miR-487a_st	0.051	5.21E-02	0.644	5.66	5.61	5.64	0.130
hsa-miR-144-star_st	-0.038	5.26E-02	0.644	5.48	5.51	5.50	0.097
hsa-miR-337-5p_st	0.083	5.27E-02	0.644	5.82	5.74	5.78	0.213
hp_hsa-mir-23a_st	0.047	5.27E-02	0.644	5.78	5.73	5.76	0.121
hp_hsa-mir-526a-1_x_st	-0.020	5.32E-02	0.644	5.33	5.34	5.33	0.050
hp_hsa-let-7d_x_st	0.037	5.32E-02	0.644	6.55	6.52	6.54	0.094
hsa-miR-106b_st	-0.276	5.33E-02	0.644	9.37	9.65	9.50	0.710
hp_hsa-mir-944_st	-0.022	5.34E-02	0.644	5.31	5.34	5.32	0.057
hsa-miR-21_st	0.234	5.37E-02	0.644	8.07	7.84	7.96	0.603
hsa-miR-4285_st	-0.045	5.38E-02	0.644	5.60	5.64	5.62	0.117
hp_hsa-mir-125b-1_x_st	0.044	5.41E-02	0.644	6.14	6.09	6.12	0.113
hsa-miR-200a-star_st	-0.203	5.45E-02	0.644	7.08	7.28	7.18	0.526
hp_hsa-mir-302a_x_st	-0.021	5.45E-02	0.644	5.30	5.32	5.31	0.054
hsa-miR-651_st	0.019	5.46E-02	0.644	5.31	5.29	5.30	0.049
hsa-miR-101_st	-0.034	5.47E-02	0.644	5.43	5.46	5.44	0.087

hp_hsa-mir-3152_x_st	-0.037	5.48E-02	0.644	5.44	5.48	5.46	0.094
hp_hsa-mir-501_x_st	-0.050	5.50E-02	0.644	5.66	5.71	5.69	0.129
hsa-let-7i_st	0.223	5.51E-02	0.644	11.12	10.89	11.01	0.579
hsa-miR-3119_st	0.023	5.53E-02	0.644	5.29	5.26	5.27	0.058
hsa-miR-193b_st	0.239	5.54E-02	0.644	8.61	8.37	8.50	0.619
hsa-let-7d_st	0.146	5.56E-02	0.644	12.10	11.95	12.03	0.378
hp_hsa-mir-3129_st	-0.021	5.62E-02	0.644	5.38	5.40	5.39	0.054
hp_hsa-mir-4298_st	0.027	5.62E-02	0.644	5.58	5.55	5.57	0.070
hsa-miR-425-star_st	-0.113	5.67E-02	0.644	6.94	7.05	7.00	0.294
hp_hsa-mir-551a_st	-0.024	5.67E-02	0.644	5.45	5.47	5.46	0.061
hsa-miR-886-3p_st	-0.336	5.68E-02	0.644	8.75	9.09	8.91	0.876
hp_hsa-mir-148a_st	0.039	5.68E-02	0.644	5.76	5.72	5.74	0.100
hsa-miR-135a_st	0.023	5.73E-02	0.644	5.30	5.27	5.29	0.059
hp_hsa-mir-1203_st	-0.025	5.74E-02	0.644	5.50	5.52	5.51	0.065
hsa-miR-192-star_st	-0.235	5.76E-02	0.644	6.91	7.15	7.02	0.615
hp_hsa-mir-28_x_st	0.054	5.79E-02	0.644	6.19	6.14	6.16	0.141
hp_hsa-mir-1248_s_st	0.093	5.80E-02	0.644	6.61	6.51	6.56	0.245
hp_hsa-mir-220a_st	-0.025	5.85E-02	0.647	5.38	5.41	5.40	0.066
hsa-miR-1307_st	-0.186	5.88E-02	0.647	8.14	8.33	8.23	0.490
hp_hsa-mir-1302-10_s_st	-0.021	5.93E-02	0.647	5.30	5.32	5.31	0.055
hp_hsa-mir-3156-1_x_st	-0.033	5.95E-02	0.647	5.57	5.60	5.58	0.086
hsa-miR-876-5p_st	0.017	6.01E-02	0.647	5.26	5.24	5.25	0.045
hp_hsa-mir-181b-2_st	-0.035	6.05E-02	0.647	5.34	5.37	5.35	0.092
hsa-miR-4301_st	0.023	6.07E-02	0.647	5.34	5.32	5.33	0.061
hp_hsa-mir-548g_x_st	-0.033	6.11E-02	0.647	5.45	5.49	5.47	0.088
hsa-miR-1291_st	-0.061	6.15E-02	0.647	5.70	5.76	5.73	0.163
hp_hsa-mir-19b-2_x_st	0.025	6.20E-02	0.647	5.42	5.40	5.41	0.065
hsa-miR-574-3p_st	0.245	6.27E-02	0.647	8.47	8.22	8.35	0.653
hsa-miR-379_st	0.156	6.32E-02	0.647	6.49	6.34	6.42	0.418
hsa-miR-3117_st	-0.019	6.35E-02	0.647	5.31	5.33	5.32	0.050
hp_hsa-mir-124-2_s_st	0.049	6.35E-02	0.647	5.57	5.52	5.55	0.132
hp_hsa-mir-22_st	0.038	6.37E-02	0.647	5.83	5.79	5.81	0.102
hsa-miR-922_st	-0.023	6.39E-02	0.647	5.35	5.37	5.36	0.060
hsa-miR-3201_st	-0.192	6.43E-02	0.647	6.26	6.45	6.35	0.515
hp_hsa-mir-1323_st	-0.033	6.45E-02	0.647	5.50	5.53	5.52	0.089
hsa-miR-3173_st	-0.032	6.49E-02	0.647	5.55	5.59	5.57	0.086
hp_hsa-mir-187_st	0.033	6.50E-02	0.647	5.71	5.68	5.69	0.089
hsa-miR-874_st	0.131	6.52E-02	0.647	7.04	6.91	6.98	0.353
hp_hsa-mir-20b_x_st	0.051	6.54E-02	0.647	5.93	5.88	5.90	0.138
hsa-miR-194_st	-0.271	6.59E-02	0.647	12.11	12.38	12.24	0.733
hp_hsa-mir-378_x_st	-0.055	6.61E-02	0.647	5.93	5.99	5.96	0.148
hsa-miR-4279_st	-0.029	6.61E-02	0.647	5.41	5.44	5.43	0.077
hp_hsa-mir-132_st	0.043	6.62E-02	0.647	5.81	5.76	5.79	0.116
hp_hsa-mir-652_x_st	-0.039	6.64E-02	0.647	5.72	5.76	5.74	0.105
hp_hsa-mir-548i-4_x_st	-0.031	6.69E-02	0.647	5.42	5.45	5.44	0.084
hsa-miR-663_st	-0.248	6.70E-02	0.647	10.33	10.58	10.45	0.672
hp_hsa-mir-4325_st	-0.024	6.70E-02	0.647	5.47	5.49	5.48	0.065

hsa-miR-4269_st	0.075	6.78E-02	0.652	5.85	5.77	5.81	0.205
hsa-miR-31_st	0.638	6.84E-02	0.655	9.51	8.87	9.20	1.740
hp_hsa-mir-936_st	-0.022	6.89E-02	0.657	5.41	5.43	5.42	0.059
hp_hsa-mir-100_x_st	0.032	6.93E-02	0.658	5.48	5.45	5.46	0.087
hsa-miR-627_st	0.021	7.08E-02	0.666	5.30	5.28	5.29	0.056
hsa-let-7c_st	0.135	7.19E-02	0.666	13.02	12.89	12.96	0.371
hsa-miR-622_st	-0.156	7.19E-02	0.666	5.97	6.13	6.05	0.432
hsa-miR-99a_st	0.327	7.20E-02	0.666	8.98	8.65	8.82	0.902
hp_hsa-mir-507_st	-0.017	7.21E-02	0.666	5.29	5.31	5.30	0.047
hp_hsa-mir-1249_st	0.030	7.23E-02	0.666	5.55	5.52	5.53	0.081
hsa-miR-182_st	-0.267	7.25E-02	0.666	8.99	9.25	9.12	0.739
hp_hsa-mir-574_st	0.058	7.25E-02	0.666	6.58	6.52	6.55	0.161
hp_hsa-mir-3171_st	-0.016	7.29E-02	0.666	5.30	5.31	5.30	0.044
hp_hsa-mir-548i-4_st	-0.030	7.35E-02	0.666	5.44	5.48	5.46	0.084
hsa-miR-544_st	-0.021	7.36E-02	0.666	5.29	5.31	5.30	0.057
hsa-miR-411-star_st	0.024	7.37E-02	0.666	5.45	5.43	5.44	0.067
hsa-miR-513a-3p_st	0.022	7.42E-02	0.666	5.38	5.36	5.37	0.060
hp_hsa-let-7a-3_st	0.020	7.44E-02	0.666	5.40	5.38	5.39	0.054
hp_hsa-mir-758_st	0.017	7.48E-02	0.667	5.38	5.36	5.37	0.046
hsa-miR-576-5p_st	0.020	7.60E-02	0.675	5.36	5.34	5.35	0.055
hsa-let-7f-2-star_st	0.018	7.65E-02	0.675	5.32	5.31	5.31	0.049
hsa-miR-138-1-star_st	0.067	7.66E-02	0.675	5.65	5.58	5.62	0.187
hsa-miR-515-3p_st	0.020	7.70E-02	0.675	5.31	5.29	5.30	0.055
hp_hsa-mir-518c_x_st	-0.020	7.73E-02	0.675	5.32	5.34	5.33	0.055
hsa-miR-124_st	0.071	7.75E-02	0.675	5.82	5.75	5.79	0.201
hsa-miR-195_st	0.288	7.80E-02	0.676	9.08	8.79	8.94	0.812
hsa-miR-330-5p_st	0.029	7.88E-02	0.676	5.47	5.44	5.46	0.081
hp_hsa-mir-1178_st	-0.025	7.91E-02	0.676	5.45	5.47	5.46	0.071
hsa-miR-30a_st	0.183	7.92E-02	0.676	7.31	7.13	7.22	0.518
hsa-miR-3175_st	-0.159	7.93E-02	0.676	7.90	8.06	7.98	0.451
hp_hsa-mir-2116_st	0.023	7.96E-02	0.676	5.45	5.43	5.44	0.066
hsa-miR-92b_st	0.123	7.97E-02	0.676	6.90	6.78	6.84	0.349
hsa-miR-217_st	0.036	8.00E-02	0.676	5.41	5.37	5.39	0.102
hp_hsa-mir-1-2_x_st	-0.018	8.08E-02	0.677	5.35	5.37	5.36	0.051
hp_hsa-mir-617_st	-0.017	8.08E-02	0.677	5.36	5.38	5.37	0.047
hsa-miR-15a_st	0.187	8.18E-02	0.683	7.06	6.87	6.97	0.532
hp_hsa-mir-514-1_s_st	0.017	8.29E-02	0.684	5.33	5.31	5.32	0.049
hsa-miR-155_st	-0.260	8.30E-02	0.684	9.61	9.87	9.74	0.744
hsa-miR-125a-3p_st	0.088	8.31E-02	0.684	6.60	6.51	6.56	0.251
hsa-miR-125b-1-star_st	0.074	8.39E-02	0.684	5.98	5.91	5.95	0.214
hsa-miR-96_st	-0.028	8.42E-02	0.684	5.39	5.42	5.41	0.079
hp_hsa-mir-764_st	0.026	8.53E-02	0.684	5.55	5.53	5.54	0.076
hp_hsa-mir-377_st	0.020	8.56E-02	0.684	5.44	5.42	5.43	0.057
hp_hsa-mir-4280_st	0.017	8.57E-02	0.684	5.37	5.36	5.36	0.050
hsa-miR-127-5p_st	0.031	8.58E-02	0.684	5.46	5.43	5.45	0.089
hp_hsa-mir-124-1_s_st	0.046	8.60E-02	0.684	5.57	5.53	5.55	0.132
hsa-miR-1292_st	-0.068	8.62E-02	0.684	5.89	5.95	5.92	0.197

hsa-miR-1265_st	0.025	8.67E-02	0.684	5.39	5.37	5.38	0.073
hp_hsa-mir-129-1_st	-0.018	8.67E-02	0.684	5.35	5.37	5.36	0.052
hp_hsa-mir-1301_st	-0.040	8.70E-02	0.684	5.65	5.69	5.67	0.116
hsa-miR-1470_st	-0.026	8.72E-02	0.684	5.50	5.53	5.52	0.076
hsa-miR-3138_st	0.086	8.74E-02	0.684	5.95	5.87	5.91	0.249
hp_hsa-mir-4296_st	0.031	8.76E-02	0.684	5.78	5.75	5.77	0.090
hp_hsa-mir-3168_st	-0.021	8.91E-02	0.684	5.37	5.39	5.38	0.061
hp_hsa-mir-106a_x_st	-0.028	8.91E-02	0.684	5.79	5.82	5.80	0.080
hp_hsa-mir-1234_st	-0.049	8.94E-02	0.684	5.79	5.84	5.82	0.142
hp_hsa-mir-1285-2_st	-0.022	8.95E-02	0.684	5.43	5.45	5.44	0.062
hsa-miR-638_st	-0.154	8.95E-02	0.684	13.21	13.36	13.28	0.450
hp_hsa-mir-4275_st	-0.067	9.01E-02	0.684	5.77	5.84	5.80	0.197
hp_hsa-mir-92b_st	0.048	9.04E-02	0.684	5.97	5.93	5.95	0.139
hsa-miR-671-3p_st	0.040	9.08E-02	0.684	5.64	5.60	5.62	0.117
hp_hsa-mir-212_st	0.026	9.14E-02	0.684	5.61	5.58	5.60	0.077
hsa-miR-363_st	0.115	9.14E-02	0.684	5.71	5.59	5.65	0.338
hsa-miR-1301_st	-0.132	9.24E-02	0.684	6.47	6.60	6.54	0.390
hsa-miR-654-3p_st	0.056	9.25E-02	0.684	5.57	5.52	5.55	0.165
hp_hsa-mir-4294_st	-0.026	9.30E-02	0.684	5.55	5.58	5.57	0.075
hp_hsa-mir-548t_x_st	-0.021	9.30E-02	0.684	5.33	5.35	5.34	0.060
hp_hsa-mir-3166_st	0.016	9.33E-02	0.684	5.35	5.34	5.34	0.046
hsa-miR-500b_st	0.018	9.33E-02	0.684	5.34	5.33	5.33	0.052
hsa-miR-369-3p_st	-0.017	9.36E-02	0.684	5.29	5.30	5.30	0.051
hp_hsa-mir-548l_x_st	-0.026	9.37E-02	0.684	5.52	5.55	5.54	0.077
hp_hsa-mir-548f-3_st	-0.015	9.43E-02	0.684	5.35	5.37	5.36	0.044
hp_hsa-mir-1260_st	-0.054	9.44E-02	0.684	6.08	6.13	6.11	0.159
hsa-miR-3130-5p_st	-0.031	9.47E-02	0.684	5.47	5.50	5.49	0.092
hp_hsa-mir-127_st	0.026	9.48E-02	0.684	5.73	5.71	5.72	0.075
hsa-miR-23a_st	0.146	9.57E-02	0.684	12.94	12.79	12.87	0.435
hp_hsa-mir-583_x_st	-0.023	9.58E-02	0.684	5.40	5.42	5.41	0.068
hp_hsa-mir-29a_x_st	0.041	9.60E-02	0.684	5.95	5.91	5.93	0.121
hsa-miR-29b-2-star_st	0.127	9.60E-02	0.684	6.36	6.23	6.30	0.380
hsa-miR-199b-5p_st	0.068	9.62E-02	0.684	5.60	5.53	5.57	0.202
hsa-miR-378c_st	-0.194	9.72E-02	0.684	8.35	8.54	8.44	0.579
hp_hsa-mir-302f_st	-0.023	9.73E-02	0.684	5.36	5.39	5.38	0.069
hp_hsa-mir-886_st	-0.220	9.74E-02	0.684	7.83	8.05	7.93	0.658
hsa-miR-146a_st	-0.292	9.74E-02	0.684	8.65	8.94	8.79	0.874
hsa-miR-599_st	0.017	9.75E-02	0.684	5.31	5.30	5.31	0.049
hp_hsa-mir-3164_st	-0.021	9.77E-02	0.684	5.36	5.38	5.37	0.061
hp_hsa-mir-548h-1_x_st	-0.021	9.79E-02	0.684	5.33	5.35	5.34	0.062
hsa-miR-181a-2-star_st	0.127	9.81E-02	0.684	6.43	6.31	6.37	0.381
hsa-miR-3065-5p_st	0.027	9.83E-02	0.684	5.42	5.40	5.41	0.081
hsa-miR-142-5p_st	0.019	9.93E-02	0.686	5.35	5.33	5.34	0.056
hp_hsa-mir-193b_st	0.040	9.98E-02	0.686	5.81	5.77	5.79	0.119
hsa-miR-3194_st	-0.042	1.00E-01	0.686	5.73	5.77	5.75	0.127
hp_hsa-mir-148a_x_st	0.035	1.00E-01	0.686	5.60	5.56	5.58	0.106
hsa-miR-664-star_st	0.122	1.00E-01	0.686	6.58	6.45	6.52	0.369

hp_hsa-mir-301a_st	-0.016	1.01E-01	0.686	5.31	5.32	5.31	0.046
hsa-miR-514b-3p_st	0.017	1.02E-01	0.686	5.29	5.28	5.29	0.050
hsa-miR-887_st	0.044	1.02E-01	0.686	5.72	5.67	5.69	0.132
hsa-miR-376a-star_st	-0.020	1.02E-01	0.686	5.34	5.36	5.35	0.058
hsa-miR-663b_st	-0.206	1.02E-01	0.686	7.88	8.08	7.98	0.625
hsa-miR-196b-star_st	-0.183	1.02E-01	0.686	6.24	6.42	6.32	0.555
hp_hsa-let-7d_st	0.040	1.04E-01	0.691	6.39	6.35	6.37	0.120
hsa-miR-675_st	-0.193	1.04E-01	0.691	6.53	6.72	6.62	0.589
hp_hsa-mir-631_st	-0.033	1.04E-01	0.691	5.56	5.59	5.58	0.100
hp_hsa-mir-1538_st	-0.033	1.04E-01	0.691	5.65	5.68	5.66	0.102
hsa-miR-377-star_st	0.039	1.04E-01	0.692	5.47	5.43	5.45	0.118
hsa-miR-4255_st	0.021	1.05E-01	0.695	5.47	5.45	5.46	0.062
hsa-miR-219-5p_st	-0.019	1.06E-01	0.696	5.28	5.30	5.29	0.058
hsa-miR-3178_st	-0.210	1.07E-01	0.700	10.05	10.26	10.15	0.645
hsa-miR-1979_st	0.128	1.07E-01	0.700	9.28	9.15	9.22	0.393
hsa-miR-376a_st	0.020	1.08E-01	0.704	5.40	5.38	5.39	0.061
hsa-miR-200c_st	-0.144	1.09E-01	0.705	13.16	13.30	13.23	0.446
hsa-miR-4295_st	0.018	1.09E-01	0.705	5.31	5.29	5.30	0.055
hp_hsa-mir-3192_st	-0.020	1.09E-01	0.705	5.39	5.41	5.40	0.062
hsa-miR-124-star_st	0.021	1.09E-01	0.705	5.39	5.37	5.38	0.064
hp_hsa-mir-124-3_x_st	0.016	1.09E-01	0.705	5.38	5.36	5.37	0.050
hp_hsa-mir-3145_x_st	-0.015	1.11E-01	0.707	5.36	5.37	5.37	0.045
hsa-miR-30c_st	0.169	1.11E-01	0.707	8.74	8.58	8.66	0.524
hp_hsa-mir-1291_s_st	-0.048	1.11E-01	0.707	5.76	5.80	5.78	0.148
hsa-miR-95_st	-0.034	1.11E-01	0.707	5.35	5.39	5.37	0.106
hp_hsa-mir-1247_st	-0.029	1.11E-01	0.707	5.52	5.55	5.54	0.091
hsa-miR-203_st	-0.311	1.12E-01	0.708	7.98	8.30	8.14	0.970
hp_hsa-mir-548e_st	-0.037	1.12E-01	0.708	5.61	5.64	5.63	0.114
hp_hsa-mir-623_st	-0.018	1.13E-01	0.711	5.36	5.37	5.37	0.054
hsa-miR-22-star_st	0.053	1.14E-01	0.711	5.60	5.54	5.57	0.167
hsa-miR-193a-5p_st	0.145	1.14E-01	0.711	7.84	7.70	7.77	0.454
hsa-miR-1908_st	-0.227	1.14E-01	0.711	11.50	11.73	11.61	0.712
hsa-miR-1_st	0.029	1.14E-01	0.711	5.41	5.38	5.40	0.089
hsa-miR-886-5p_st	-0.266	1.15E-01	0.711	7.99	8.25	8.11	0.834
hsa-miR-491-5p_st	-0.104	1.15E-01	0.712	6.32	6.42	6.37	0.326
hp_hsa-mir-339_st	-0.043	1.16E-01	0.712	6.14	6.18	6.16	0.136
hp_hsa-mir-765_st	-0.016	1.16E-01	0.712	5.41	5.43	5.42	0.051
hsa-miR-497_st	0.202	1.18E-01	0.715	7.47	7.27	7.37	0.638
hsa-let-7a_st	0.137	1.18E-01	0.715	13.09	12.95	13.02	0.434
hp_hsa-mir-379_st	0.035	1.18E-01	0.715	5.53	5.49	5.51	0.110
hsa-miR-607_st	-0.018	1.18E-01	0.715	5.39	5.41	5.40	0.057
hp_hsa-mir-182_st	-0.059	1.18E-01	0.715	6.30	6.36	6.33	0.186
hp_hsa-mir-3161_st	0.017	1.18E-01	0.715	5.37	5.35	5.36	0.053
hsa-let-7c-star_st	-0.019	1.19E-01	0.719	5.33	5.35	5.34	0.059
hsa-miR-302e_st	0.020	1.19E-01	0.719	5.40	5.38	5.39	0.063
hsa-miR-23a-star_st	0.094	1.21E-01	0.725	6.40	6.30	6.35	0.300
hp_hsa-mir-548p_x_st	-0.015	1.22E-01	0.726	5.33	5.35	5.34	0.049

hsa-miR-3191_st	-0.031	1.23E-01	0.726	5.58	5.61	5.60	0.099
hp_hsa-mir-614_st	-0.023	1.23E-01	0.726	5.51	5.53	5.52	0.075
hsa-miR-302d-star_st	0.023	1.23E-01	0.726	5.54	5.51	5.52	0.073
hsa-miR-2054_st	0.015	1.23E-01	0.726	5.28	5.27	5.27	0.047
hsa-miR-4253_st	0.097	1.23E-01	0.726	6.23	6.13	6.18	0.311
hp_hsa-mir-548f-4_st	-0.017	1.23E-01	0.726	5.40	5.42	5.41	0.055
hp_hsa-mir-500b_x_st	-0.036	1.24E-01	0.726	5.87	5.90	5.88	0.115
hp_hsa-mir-770_st	-0.037	1.24E-01	0.727	5.82	5.85	5.83	0.121
hsa-miR-372_st	-0.020	1.25E-01	0.727	5.33	5.35	5.34	0.063
hsa-miR-888_st	-0.015	1.25E-01	0.727	5.28	5.30	5.29	0.049
hp_hsa-mir-125b-2_st	0.027	1.25E-01	0.727	5.43	5.40	5.42	0.088
hsa-miR-3181_st	-0.055	1.26E-01	0.727	5.65	5.71	5.68	0.177
hsa-miR-924_st	0.018	1.26E-01	0.727	5.36	5.34	5.35	0.058
hp_hsa-let-7e_st	0.037	1.27E-01	0.727	5.88	5.84	5.86	0.121
hsa-miR-1204_st	-0.020	1.27E-01	0.727	5.33	5.35	5.34	0.065
hp_hsa-mir-1275_st	-0.035	1.28E-01	0.727	5.94	5.98	5.96	0.115
hp_hsa-mir-3125_st	-0.019	1.28E-01	0.727	5.43	5.45	5.44	0.063
hsa-miR-371-3p_st	-0.018	1.28E-01	0.727	5.36	5.38	5.37	0.056
hp_hsa-let-7g_x_st	0.031	1.29E-01	0.727	5.79	5.76	5.78	0.100
hsa-miR-635_st	-0.030	1.29E-01	0.727	5.45	5.48	5.46	0.099
hsa-miR-3177_st	-0.044	1.30E-01	0.727	5.88	5.93	5.91	0.144
hp_hsa-mir-105-1_s_st	-0.020	1.30E-01	0.727	5.37	5.39	5.38	0.066
hsa-miR-210_st	-0.267	1.30E-01	0.727	9.48	9.75	9.61	0.875
hsa-miR-1909_st	-0.221	1.30E-01	0.727	8.14	8.36	8.25	0.725
hp_hsa-mir-195_st	0.029	1.31E-01	0.727	5.82	5.79	5.80	0.096
hp_hsa-mir-524_x_st	0.022	1.31E-01	0.727	5.45	5.42	5.43	0.073
hsa-miR-192_st	-0.240	1.31E-01	0.727	11.09	11.33	11.21	0.788
hsa-miR-146b-5p_st	0.208	1.31E-01	0.727	7.59	7.38	7.49	0.682
hp_hsa-mir-485_st	0.020	1.31E-01	0.727	5.45	5.43	5.44	0.066
hp_hsa-mir-933_st	-0.052	1.31E-01	0.727	6.32	6.37	6.35	0.170
hsa-miR-302b_st	-0.017	1.32E-01	0.727	5.27	5.28	5.27	0.056
hp_hsa-mir-98_st	0.027	1.32E-01	0.728	5.71	5.68	5.70	0.088
hsa-miR-562_st	-0.017	1.32E-01	0.728	5.27	5.28	5.28	0.054
hsa-miR-570_st	-0.041	1.34E-01	0.735	5.53	5.57	5.55	0.136
hp_hsa-mir-200b_x_st	-0.052	1.34E-01	0.735	6.65	6.71	6.68	0.172
hp_hsa-mir-190_st	-0.023	1.35E-01	0.735	5.42	5.44	5.43	0.077
hsa-miR-1228-star_st	-0.212	1.36E-01	0.738	11.33	11.55	11.44	0.703
hp_hsa-mir-632_st	-0.034	1.36E-01	0.739	5.84	5.87	5.85	0.111
hsa-miR-365_st	0.019	1.36E-01	0.739	5.35	5.33	5.34	0.062
hsa-miR-3160_st	0.019	1.37E-01	0.741	5.48	5.46	5.47	0.064
hp_hsa-mir-651_st	-0.022	1.38E-01	0.741	5.40	5.42	5.41	0.072
hp_hsa-mir-675_st	-0.063	1.38E-01	0.741	5.95	6.01	5.98	0.210
hp_hsa-let-7a-2_st	0.017	1.38E-01	0.741	5.39	5.37	5.38	0.057
hsa-miR-4284_st	-0.203	1.38E-01	0.741	10.05	10.25	10.15	0.679
hp_hsa-mir-1274a_st	-0.044	1.39E-01	0.741	5.70	5.74	5.72	0.147
hp_hsa-mir-4318_st	-0.014	1.40E-01	0.743	5.38	5.39	5.38	0.047
hp_hsa-mir-506_st	0.014	1.40E-01	0.743	5.35	5.34	5.34	0.047

hsa-miR-520a-3p_st	0.017	1.40E-01	0.744	5.31	5.30	5.30	0.057
hsa-miR-1976_st	-0.025	1.41E-01	0.746	5.42	5.45	5.43	0.083
hsa-miR-548k_st	0.018	1.42E-01	0.746	5.37	5.36	5.37	0.062
hp_hsa-mir-133a-2_x_st	0.018	1.42E-01	0.746	5.39	5.37	5.38	0.061
hp_hsa-mir-3120_s_st	-0.022	1.42E-01	0.746	5.60	5.62	5.61	0.072
hp_hsa-mir-196b_st	-0.045	1.43E-01	0.746	5.52	5.57	5.54	0.151
hsa-miR-769-3p_st	-0.055	1.43E-01	0.746	5.71	5.76	5.73	0.185
hp_hsa-mir-362_st	-0.045	1.43E-01	0.746	5.88	5.93	5.91	0.154
hsa-miR-33a-star_st	-0.014	1.43E-01	0.746	5.30	5.31	5.30	0.048
hsa-miR-652_st	-0.152	1.44E-01	0.748	8.34	8.49	8.41	0.516
hsa-miR-133a_st	0.164	1.45E-01	0.751	6.08	5.92	6.00	0.558
hp_hsa-mir-320d-2_x_st	0.023	1.45E-01	0.751	5.55	5.53	5.54	0.078
hp_hsa-mir-502_st	-0.022	1.46E-01	0.753	5.60	5.62	5.61	0.073
hp_hsa-mir-2355_st	0.019	1.47E-01	0.755	5.40	5.38	5.39	0.064
hsa-miR-3202_st	0.024	1.48E-01	0.755	5.50	5.48	5.49	0.081
hsa-miR-431_st	0.040	1.48E-01	0.755	5.61	5.57	5.59	0.136
hp_hsa-mir-520c_x_st	0.018	1.48E-01	0.755	5.37	5.35	5.36	0.061
hsa-miR-568_st	0.013	1.48E-01	0.755	5.29	5.27	5.28	0.044
hsa-miR-3128_st	-0.095	1.50E-01	0.762	5.81	5.91	5.86	0.327
hsa-miR-548p_st	-0.018	1.50E-01	0.762	5.32	5.34	5.33	0.063
hsa-miR-212_st	0.074	1.51E-01	0.762	6.03	5.96	6.00	0.255
hp_hsa-mir-1202_st	-0.029	1.51E-01	0.762	5.80	5.83	5.81	0.101
hp_hsa-mir-491_st	-0.032	1.52E-01	0.762	5.84	5.87	5.85	0.109
hp_hsa-mir-191_st	-0.028	1.52E-01	0.762	5.82	5.85	5.84	0.097
hsa-miR-520a-5p_st	-0.016	1.53E-01	0.762	5.29	5.30	5.30	0.054
hp_hsa-mir-299_st	0.019	1.53E-01	0.762	5.45	5.43	5.44	0.064
hp_hsa-mir-1254_st	-0.018	1.53E-01	0.762	5.49	5.50	5.50	0.061
hsa-miR-558_st	0.016	1.53E-01	0.762	5.31	5.29	5.30	0.055
hp_hsa-mir-520h_s_st	-0.071	1.54E-01	0.762	5.98	6.05	6.01	0.245
hsa-miR-138-2-star_st	0.021	1.54E-01	0.762	5.54	5.52	5.53	0.072
hp_hsa-mir-627_x_st	-0.015	1.54E-01	0.762	5.42	5.44	5.43	0.051
hp_hsa-mir-1289-1_x_st	-0.038	1.54E-01	0.762	5.79	5.83	5.81	0.132
hsa-miR-1271_st	0.097	1.55E-01	0.762	6.18	6.09	6.14	0.338
hsa-miR-2117_st	-0.015	1.55E-01	0.764	5.27	5.29	5.28	0.050
hp_hsa-let-7i_st	0.029	1.56E-01	0.765	5.76	5.73	5.74	0.101
hp_hsa-mir-192_st	-0.030	1.57E-01	0.768	5.58	5.61	5.60	0.105
hsa-let-7d-star_st	-0.054	1.57E-01	0.768	5.65	5.70	5.67	0.187
hp_hsa-mir-455_st	0.033	1.58E-01	0.769	5.75	5.72	5.73	0.116
hsa-miR-493-star_st	0.022	1.59E-01	0.774	5.36	5.34	5.35	0.075
hp_hsa-mir-580_st	-0.012	1.60E-01	0.776	5.34	5.36	5.35	0.043
hsa-miR-10a_st	0.216	1.61E-01	0.776	8.77	8.55	8.67	0.763
hp_hsa-mir-451_st	0.033	1.61E-01	0.776	5.60	5.57	5.59	0.117
hsa-miR-148a_st	0.149	1.62E-01	0.778	6.61	6.46	6.54	0.527
hsa-miR-489_st	0.077	1.62E-01	0.778	5.82	5.74	5.78	0.272
hp_hsa-mir-412_st	-0.020	1.62E-01	0.779	5.42	5.44	5.43	0.070
hsa-miR-455-5p_st	0.038	1.65E-01	0.782	5.54	5.51	5.53	0.136
hp_hsa-mir-1979_st	-0.060	1.65E-01	0.782	5.80	5.86	5.83	0.214

hp_hsa-mir-223_st	0.015	1.66E-01	0.782	5.45	5.44	5.44	0.054
hp_hsa-mir-516a-1_s_st	0.018	1.67E-01	0.782	5.33	5.31	5.32	0.063
hsa-miR-509-3p_st	0.130	1.67E-01	0.782	5.74	5.61	5.68	0.467
hsa-miR-518e-star_st	-0.018	1.67E-01	0.782	5.39	5.41	5.40	0.065
hp_hsa-mir-1302-9_s_st	-0.014	1.67E-01	0.782	5.31	5.33	5.32	0.049
hp_hsa-mir-520g_s_st	-0.066	1.68E-01	0.782	5.96	6.03	5.99	0.236
hsa-miR-1205_st	0.018	1.68E-01	0.782	5.36	5.34	5.35	0.065
hp_hsa-mir-320c-2_x_st	0.031	1.68E-01	0.782	6.00	5.97	5.99	0.111
hsa-miR-4326_st	0.019	1.69E-01	0.782	5.35	5.33	5.34	0.066
hp_hsa-mir-1909_st	-0.075	1.70E-01	0.782	6.33	6.40	6.36	0.272
hsa-let-7g_st	0.161	1.71E-01	0.782	8.92	8.76	8.85	0.580
hp_hsa-mir-93_st	-0.033	1.71E-01	0.782	6.03	6.06	6.05	0.119
hp_hsa-mir-3144_st	0.015	1.72E-01	0.782	5.43	5.42	5.43	0.055
hsa-miR-4294_st	0.028	1.72E-01	0.782	5.57	5.54	5.55	0.101
hsa-miR-380-star_st	0.014	1.72E-01	0.782	5.35	5.34	5.34	0.050
hp_hsa-mir-519c_x_st	-0.014	1.72E-01	0.782	5.37	5.38	5.38	0.051
hp_hsa-mir-520f_st	-0.018	1.72E-01	0.782	5.40	5.41	5.40	0.063
hsa-miR-22_st	0.137	1.72E-01	0.782	10.32	10.18	10.25	0.498
hsa-miR-548d-3p_st	0.016	1.73E-01	0.782	5.39	5.38	5.38	0.056
hsa-miR-29a_st	0.177	1.73E-01	0.782	9.82	9.64	9.73	0.643
hsa-miR-410_st	0.018	1.73E-01	0.782	5.37	5.35	5.36	0.066
hsa-miR-518a-3p_st	-0.021	1.74E-01	0.782	5.48	5.50	5.49	0.075
hp_hsa-mir-633_st	0.016	1.74E-01	0.782	5.40	5.39	5.40	0.058
hp_hsa-mir-663b_x_st	-0.084	1.74E-01	0.782	6.41	6.50	6.45	0.306
hsa-miR-1975_st	0.087	1.74E-01	0.782	13.57	13.48	13.52	0.317
hp_hsa-mir-630_st	0.015	1.75E-01	0.782	5.35	5.34	5.34	0.054
hsa-miR-513b_st	-0.032	1.75E-01	0.782	5.53	5.56	5.55	0.117
hp_hsa-mir-3188_st	-0.048	1.75E-01	0.782	6.46	6.51	6.48	0.174
hp_hsa-mir-107_x_st	-0.015	1.75E-01	0.782	5.95	5.96	5.95	0.053
hp_hsa-mir-200c_st	-0.041	1.75E-01	0.782	7.02	7.06	7.04	0.150
hsa-miR-1274b_st	-0.216	1.75E-01	0.782	8.96	9.18	9.07	0.789
hsa-miR-1276_st	-0.019	1.75E-01	0.782	5.38	5.40	5.39	0.068
hp_hsa-mir-4297_st	-0.047	1.76E-01	0.782	5.82	5.87	5.84	0.171
hp_hsa-mir-618_st	-0.017	1.76E-01	0.782	5.39	5.40	5.39	0.062
hp_hsa-mir-124-1_x_st	0.014	1.76E-01	0.782	5.38	5.37	5.38	0.049
hsa-miR-2115-star_st	0.028	1.77E-01	0.782	5.62	5.59	5.60	0.102
hsa-miR-92a-2-star_st	-0.021	1.77E-01	0.782	5.35	5.37	5.36	0.078
hsa-miR-1264_st	-0.014	1.78E-01	0.783	5.28	5.30	5.29	0.050
hp_hsa-mir-218-1_st	0.014	1.78E-01	0.783	5.40	5.39	5.40	0.052
hp_hsa-mir-524_st	0.039	1.78E-01	0.783	5.62	5.58	5.60	0.144
hsa-miR-1243_st	-0.013	1.78E-01	0.783	5.34	5.35	5.35	0.047
hp_hsa-mir-486_x_st	0.054	1.79E-01	0.784	5.77	5.72	5.75	0.198
hsa-miR-1469_st	-0.167	1.80E-01	0.784	11.76	11.93	11.84	0.617
hsa-miR-3199_st	-0.016	1.81E-01	0.784	5.33	5.35	5.34	0.058
hp_hsa-mir-1304_st	0.017	1.81E-01	0.784	5.43	5.42	5.42	0.063
hp_hsa-mir-4306_st	0.017	1.81E-01	0.784	5.42	5.40	5.41	0.062
hsa-miR-105_st	-0.099	1.81E-01	0.784	5.41	5.51	5.46	0.367

hsa-miR-369-5p_st	0.015	1.81E-01	0.784	5.39	5.38	5.38	0.056
hsa-let-7f-1-star_st	0.032	1.82E-01	0.784	5.69	5.66	5.67	0.119
hp_hsa-mir-1-1_x_st	-0.013	1.82E-01	0.784	5.33	5.34	5.34	0.049
hsa-miR-520c-3p_st	0.016	1.82E-01	0.784	5.41	5.39	5.40	0.059
hp_hsa-mir-222_st	-0.039	1.83E-01	0.784	6.50	6.54	6.52	0.144
hp_hsa-mir-591_st	0.018	1.83E-01	0.784	5.55	5.53	5.54	0.066
hsa-miR-190b_st	0.015	1.83E-01	0.784	5.41	5.40	5.40	0.054
hsa-miR-98_st	0.051	1.84E-01	0.785	6.02	5.96	5.99	0.191
hsa-miR-451_st	0.127	1.84E-01	0.785	6.29	6.17	6.23	0.475
hsa-miR-548q_st	0.030	1.85E-01	0.789	5.54	5.51	5.53	0.112
hsa-miR-361-3p_st	0.029	1.87E-01	0.793	5.50	5.47	5.49	0.107
hp_hsa-mir-7-2_st	-0.031	1.89E-01	0.796	5.73	5.76	5.75	0.116
hsa-miR-208a_st	-0.017	1.90E-01	0.796	5.38	5.39	5.38	0.062
hsa-miR-133b_st	0.087	1.90E-01	0.796	5.73	5.64	5.69	0.330
hp_hsa-mir-3197_st	-0.043	1.90E-01	0.796	6.03	6.07	6.05	0.160
hsa-miR-379-star_st	0.015	1.90E-01	0.796	5.32	5.31	5.31	0.057
hsa-miR-183-star_st	-0.054	1.91E-01	0.796	5.94	5.99	5.96	0.206
hp_hsa-mir-4285_st	-0.052	1.91E-01	0.796	6.02	6.07	6.05	0.195
hsa-miR-490-5p_st	0.085	1.91E-01	0.796	5.82	5.74	5.78	0.323
hp_hsa-mir-3174_st	0.013	1.91E-01	0.796	5.37	5.36	5.36	0.048
hsa-miR-662_st	-0.024	1.91E-01	0.796	5.57	5.59	5.58	0.090
hsa-miR-1244_st	-0.104	1.92E-01	0.796	6.06	6.16	6.11	0.395
hp_hsa-mir-548e_x_st	-0.027	1.93E-01	0.796	5.52	5.55	5.53	0.104
hp_hsa-mir-378c_x_st	-0.021	1.93E-01	0.796	5.89	5.91	5.90	0.079
hsa-miR-130a_st	0.135	1.93E-01	0.796	7.71	7.57	7.64	0.513
hsa-miR-603_st	-0.043	1.93E-01	0.796	5.56	5.60	5.58	0.164
hp_hsa-mir-101-2_st	0.012	1.93E-01	0.796	5.35	5.34	5.34	0.045
hsa-miR-4254_st	0.018	1.94E-01	0.797	5.44	5.42	5.43	0.068
hp_hsa-mir-563_st	0.012	1.95E-01	0.797	5.33	5.32	5.32	0.044
hp_hsa-mir-620_x_st	-0.021	1.95E-01	0.797	5.39	5.41	5.40	0.079
hp_hsa-mir-1269_st	0.021	1.95E-01	0.797	5.56	5.54	5.55	0.081
hp_hsa-mir-144_st	-0.018	1.95E-01	0.797	5.44	5.46	5.45	0.066
hp_hsa-mir-378_st	-0.031	1.96E-01	0.800	5.66	5.69	5.68	0.117
hsa-miR-1273d_st	0.057	1.97E-01	0.800	6.13	6.07	6.10	0.217
hp_hsa-mir-146b_st	0.028	1.98E-01	0.803	5.63	5.60	5.61	0.108
hsa-miR-3148_st	0.021	1.99E-01	0.803	5.46	5.44	5.45	0.079
hp_hsa-mir-376b_x_st	-0.018	1.99E-01	0.803	5.38	5.40	5.39	0.069
hp_hsa-mir-890_st	-0.017	2.00E-01	0.804	5.40	5.42	5.41	0.064
hsa-miR-3149_st	0.027	2.00E-01	0.804	5.52	5.50	5.51	0.104
hsa-miR-935_st	-0.039	2.01E-01	0.804	5.55	5.59	5.57	0.149
hsa-miR-129-3p_st	-0.019	2.01E-01	0.804	5.47	5.49	5.48	0.071
hp_hsa-mir-449b_x_st	0.018	2.01E-01	0.804	5.47	5.45	5.46	0.068
hp_hsa-mir-584_st	0.021	2.02E-01	0.804	5.56	5.54	5.55	0.080
hsa-miR-100-star_st	-0.017	2.02E-01	0.804	5.39	5.40	5.39	0.064
hp_hsa-mir-486_st	-0.019	2.02E-01	0.804	5.48	5.49	5.49	0.072
hsa-miR-567_st	0.015	2.02E-01	0.804	5.33	5.32	5.32	0.056
hsa-miR-4307_st	0.014	2.02E-01	0.804	5.31	5.30	5.31	0.054

hp_hsa-mir-4320_st	-0.034	2.04E-01	0.807	5.71	5.74	5.72	0.133
hp_hsa-mir-33a_st	-0.012	2.04E-01	0.807	5.30	5.31	5.31	0.047
hsa-miR-3180-3p_st	-0.157	2.05E-01	0.808	7.39	7.54	7.46	0.614
hp_hsa-mir-891a_x_st	-0.014	2.05E-01	0.808	5.37	5.38	5.37	0.052
hsa-miR-302a_st	-0.014	2.06E-01	0.809	5.30	5.31	5.31	0.054
hp_hsa-mir-512-1_s_st	-0.016	2.07E-01	0.812	5.51	5.52	5.51	0.064
hp_hsa-mir-302b_st	0.013	2.08E-01	0.815	5.34	5.33	5.33	0.052
hsa-miR-592_st	-0.050	2.08E-01	0.815	5.40	5.45	5.43	0.197
hp_hsa-mir-489_st	-0.017	2.09E-01	0.818	5.53	5.55	5.54	0.067
hp_hsa-mir-424_st	0.025	2.10E-01	0.818	5.61	5.58	5.60	0.096
hp_hsa-mir-3118-4_x_st	-0.014	2.10E-01	0.819	5.30	5.32	5.31	0.053
hsa-miR-583_st	-0.018	2.13E-01	0.825	5.37	5.39	5.38	0.069
hsa-miR-106a_st	-0.201	2.14E-01	0.825	11.42	11.62	11.52	0.800
hp_hsa-mir-583_st	-0.015	2.15E-01	0.825	5.49	5.51	5.50	0.059
hp_hsa-mir-548f-1_x_st	-0.025	2.15E-01	0.825	5.47	5.49	5.48	0.100
hp_hsa-mir-220a_s_st	-0.024	2.15E-01	0.825	5.62	5.64	5.63	0.094
hp_hsa-mir-1915_st	-0.037	2.15E-01	0.825	6.05	6.08	6.06	0.148
hsa-miR-3074_st	0.017	2.15E-01	0.825	5.42	5.41	5.42	0.069
hp_hsa-mir-3162_st	0.035	2.16E-01	0.825	5.83	5.80	5.81	0.138
hp_hsa-mir-188_st	-0.026	2.16E-01	0.825	5.64	5.67	5.65	0.102
hp_hsa-mir-29b-2_st	0.018	2.16E-01	0.825	5.41	5.39	5.40	0.071
hsa-miR-1251_st	0.015	2.16E-01	0.825	5.43	5.42	5.42	0.058
hsa-miR-4259_st	-0.050	2.17E-01	0.825	5.72	5.77	5.75	0.201
hp_hsa-mir-762_st	-0.034	2.17E-01	0.825	6.65	6.68	6.66	0.136
hsa-miR-3176_st	-0.102	2.17E-01	0.825	6.63	6.73	6.68	0.410
hp_hsa-mir-3136_st	-0.014	2.17E-01	0.826	5.39	5.40	5.39	0.054
hsa-miR-3171_st	0.012	2.18E-01	0.828	5.31	5.30	5.30	0.049
hsa-miR-522-star_st	-0.013	2.19E-01	0.828	5.31	5.33	5.32	0.053
hp_hsa-mir-518a-2_x_st	-0.020	2.19E-01	0.828	5.60	5.62	5.60	0.079
hsa-miR-145-star_st	0.035	2.21E-01	0.830	5.52	5.49	5.51	0.140
hsa-miR-324-5p_st	-0.098	2.21E-01	0.830	8.01	8.10	8.05	0.394
hsa-miR-130b-star_st	-0.014	2.21E-01	0.830	5.31	5.32	5.31	0.055
hsa-miR-1274a_st	-0.184	2.22E-01	0.830	6.88	7.07	6.97	0.742
hp_hsa-mir-330_st	-0.022	2.22E-01	0.830	5.54	5.56	5.55	0.090
hp_hsa-mir-487a_st	0.017	2.22E-01	0.830	5.49	5.47	5.48	0.067
hsa-miR-2116-star_st	0.020	2.23E-01	0.830	5.48	5.46	5.47	0.081
hsa-miR-24-2-star_st	0.081	2.23E-01	0.830	6.29	6.21	6.25	0.330
hp_hsa-mir-1243_st	0.016	2.23E-01	0.830	5.42	5.41	5.42	0.064
hp_hsa-mir-337_x_st	0.015	2.24E-01	0.832	5.52	5.51	5.52	0.062
hp_hsa-mir-1204_st	0.023	2.25E-01	0.832	5.45	5.43	5.44	0.093
hp_hsa-mir-138-1_st	-0.021	2.26E-01	0.832	5.63	5.65	5.64	0.087
hp_hsa-mir-598_st	0.017	2.26E-01	0.832	5.60	5.58	5.59	0.069
hsa-miR-449c_st	-0.016	2.26E-01	0.832	5.33	5.35	5.34	0.064
hp_hsa-mir-1200_st	-0.017	2.27E-01	0.832	5.42	5.43	5.42	0.067
hsa-miR-767-5p_st	-0.089	2.27E-01	0.832	5.36	5.45	5.40	0.365
hp_hsa-mir-363_st	-0.017	2.28E-01	0.832	5.40	5.41	5.40	0.068
hp_hsa-mir-1280_st	0.024	2.28E-01	0.832	5.98	5.96	5.97	0.099

hsa-miR-518c-star_st	0.024	2.29E-01	0.832	5.53	5.50	5.52	0.099
hsa-miR-198_st	0.043	2.29E-01	0.832	6.01	5.97	5.99	0.177
hsa-miR-612_st	-0.033	2.31E-01	0.832	5.55	5.59	5.57	0.136
hp_hsa-mir-345_st	0.019	2.31E-01	0.832	5.62	5.60	5.61	0.079
hp_hsa-mir-1252_x_st	-0.012	2.32E-01	0.832	5.38	5.39	5.38	0.051
hp_hsa-mir-548h-3_x_st	-0.012	2.32E-01	0.832	5.34	5.35	5.34	0.050
hsa-miR-3126-3p_st	-0.016	2.32E-01	0.832	5.40	5.42	5.41	0.066
hp_hsa-mir-3148_x_st	-0.017	2.33E-01	0.832	5.42	5.44	5.43	0.068
hsa-miR-519b-5p_st	0.015	2.33E-01	0.832	5.41	5.39	5.40	0.063
hp_hsa-mir-1228_st	-0.035	2.33E-01	0.832	5.99	6.02	6.00	0.145
hp_hsa-mir-4284_st	-0.046	2.33E-01	0.832	7.16	7.21	7.18	0.191
hsa-miR-335_st	-0.048	2.34E-01	0.832	5.67	5.72	5.69	0.200
hsa-miR-92a-1-star_st	0.056	2.34E-01	0.832	5.72	5.67	5.69	0.232
hp_hsa-mir-3177_st	-0.015	2.34E-01	0.832	5.61	5.62	5.61	0.064
hp_hsa-mir-3065_s_st	0.014	2.34E-01	0.832	5.35	5.33	5.34	0.056
hsa-miR-3127_st	0.034	2.34E-01	0.832	5.73	5.69	5.71	0.140
hp_hsa-mir-196a-1_x_st	-0.018	2.35E-01	0.832	5.44	5.46	5.45	0.074
hp_hsa-mir-138-2_x_st	0.018	2.35E-01	0.832	5.54	5.52	5.53	0.074
hp_hsa-mir-139_st	0.031	2.35E-01	0.832	5.77	5.74	5.76	0.130
hsa-miR-1273_st	0.021	2.35E-01	0.832	5.47	5.45	5.46	0.088
hp_hsa-mir-558_x_st	0.012	2.35E-01	0.832	5.33	5.32	5.33	0.051
hp_hsa-mir-877_st	-0.023	2.35E-01	0.832	5.70	5.73	5.71	0.097
hsa-miR-4261_st	0.054	2.36E-01	0.832	5.91	5.86	5.89	0.226
hp_hsa-mir-3202-2_s_st	-0.012	2.37E-01	0.832	5.32	5.34	5.33	0.050
hp_hsa-mir-155_st	-0.063	2.37E-01	0.832	6.66	6.72	6.69	0.261
hsa-miR-214_st	0.177	2.37E-01	0.832	11.24	11.06	11.15	0.741
hp_hsa-mir-3130-2_s_st	0.015	2.37E-01	0.832	5.41	5.40	5.40	0.064
hsa-miR-185_st	-0.093	2.38E-01	0.832	9.36	9.45	9.41	0.388
hsa-miR-2114_st	0.015	2.39E-01	0.832	5.34	5.32	5.33	0.064
hsa-miR-16_st	0.109	2.39E-01	0.832	11.84	11.73	11.78	0.459
hp_hsa-mir-320b-1_x_st	-0.018	2.39E-01	0.832	5.53	5.55	5.54	0.077
hsa-miR-365-star_st	-0.037	2.40E-01	0.832	5.77	5.81	5.79	0.157
hsa-miR-29b_st	0.061	2.40E-01	0.832	5.68	5.61	5.65	0.256
hsa-miR-296-3p_st	-0.057	2.41E-01	0.832	6.11	6.17	6.14	0.242
hsa-miR-101-star_st	0.016	2.41E-01	0.832	5.31	5.29	5.30	0.068
hsa-miR-720_st	-0.155	2.41E-01	0.832	12.31	12.46	12.38	0.654
hp_hsa-mir-2117_st	-0.018	2.41E-01	0.832	5.44	5.46	5.45	0.074
hsa-miR-507_st	0.012	2.42E-01	0.832	5.28	5.27	5.28	0.049
hp_hsa-mir-1226_st	-0.033	2.42E-01	0.832	5.76	5.79	5.77	0.140
hsa-miR-516b_st	-0.015	2.42E-01	0.834	5.41	5.43	5.42	0.061
hp_hsa-mir-548v_x_st	0.015	2.43E-01	0.834	5.41	5.39	5.40	0.064
hp_hsa-mir-3182_st	-0.019	2.44E-01	0.834	5.45	5.47	5.46	0.082
hsa-miR-18a_st	-0.222	2.45E-01	0.834	7.78	8.01	7.89	0.944
hsa-miR-668_st	-0.032	2.45E-01	0.834	5.64	5.67	5.65	0.135
hp_hsa-mir-548n_st	-0.018	2.45E-01	0.834	5.57	5.58	5.58	0.075
hp_hsa-mir-3179-3_s_st	0.014	2.45E-01	0.834	5.47	5.46	5.47	0.060
hp_hsa-mir-577_st	0.013	2.46E-01	0.834	5.35	5.33	5.34	0.053

hsa-miR-9_st	-0.010	2.47E-01	0.834	5.27	5.28	5.27	0.043
hp_hsa-mir-550-2_s_st	0.025	2.47E-01	0.834	5.84	5.81	5.82	0.106
hsa-miR-604_st	0.023	2.47E-01	0.834	5.59	5.57	5.58	0.099
hp_hsa-mir-605_st	-0.013	2.48E-01	0.834	5.46	5.48	5.47	0.055
hsa-miR-424-star_st	0.103	2.48E-01	0.834	6.36	6.26	6.31	0.440
hsa-miR-130b_st	-0.117	2.48E-01	0.834	8.40	8.52	8.46	0.501
hp_hsa-mir-509-3_s_st	0.028	2.48E-01	0.834	5.40	5.38	5.39	0.121
hsa-miR-564_st	-0.037	2.48E-01	0.834	5.90	5.93	5.91	0.158
hp_hsa-mir-380_st	0.012	2.49E-01	0.834	5.33	5.32	5.33	0.049
hp_hsa-mir-320d-1_st	0.013	2.49E-01	0.834	5.31	5.29	5.30	0.053
hsa-miR-3185_st	-0.207	2.50E-01	0.834	9.83	10.04	9.93	0.889
hsa-miR-518a-5p_st	0.015	2.50E-01	0.834	5.36	5.35	5.35	0.062
hsa-miR-521_st	-0.012	2.50E-01	0.834	5.35	5.36	5.36	0.052
hsa-miR-1206_st	-0.011	2.51E-01	0.834	5.28	5.29	5.29	0.047
hp_hsa-mir-1302-4_st	0.014	2.52E-01	0.834	5.39	5.37	5.38	0.060
hp_hsa-mir-1270-2_s_st	0.011	2.52E-01	0.834	5.38	5.37	5.37	0.047
hp_hsa-mir-1182_st	-0.017	2.53E-01	0.834	5.54	5.56	5.55	0.072
hsa-miR-23b-star_st	0.060	2.53E-01	0.834	5.90	5.84	5.87	0.261
hsa-miR-412_st	0.015	2.54E-01	0.834	5.42	5.41	5.42	0.066
hp_hsa-mir-335_st	-0.021	2.54E-01	0.834	5.62	5.64	5.63	0.091
hp_hsa-mir-3154_st	0.052	2.54E-01	0.834	6.45	6.40	6.43	0.225
hsa-miR-1972_st	-0.056	2.54E-01	0.834	6.78	6.83	6.81	0.242
hp_hsa-mir-761_st	-0.012	2.54E-01	0.834	5.41	5.42	5.41	0.050
hsa-miR-1282_st	0.012	2.55E-01	0.834	5.26	5.25	5.25	0.050
hp_hsa-mir-1976_st	-0.047	2.55E-01	0.834	6.13	6.17	6.15	0.204
hsa-miR-3118_st	0.026	2.55E-01	0.834	5.37	5.34	5.36	0.113
hp_hsa-mir-3150_st	-0.024	2.55E-01	0.834	5.86	5.88	5.87	0.105
hsa-miR-188-5p_st	-0.058	2.56E-01	0.834	6.08	6.14	6.11	0.254
hp_hsa-mir-1262_x_st	-0.016	2.56E-01	0.834	5.36	5.37	5.37	0.067
hsa-miR-186-star_st	0.015	2.57E-01	0.834	5.31	5.29	5.30	0.064
hp_hsa-mir-1307_st	-0.014	2.57E-01	0.834	5.42	5.44	5.43	0.060
hsa-miR-508-5p_st	-0.018	2.58E-01	0.834	5.49	5.51	5.50	0.078
hsa-miR-1913_st	-0.054	2.58E-01	0.834	5.86	5.91	5.88	0.235
hsa-miR-637_st	-0.063	2.58E-01	0.834	5.87	5.93	5.90	0.274
hsa-miR-1910_st	-0.132	2.59E-01	0.834	7.13	7.27	7.20	0.577
hsa-miR-138_st	-0.102	2.59E-01	0.834	5.97	6.08	6.02	0.445
hsa-miR-7-2-star_st	0.018	2.59E-01	0.834	5.59	5.57	5.58	0.079
hsa-miR-514_st	0.013	2.59E-01	0.834	5.31	5.30	5.30	0.054
hsa-miR-762_st	-0.130	2.60E-01	0.835	12.38	12.51	12.44	0.571
hsa-miR-1180_st	-0.076	2.62E-01	0.837	6.36	6.44	6.40	0.335
hp_hsa-mir-374a_st	-0.012	2.62E-01	0.837	5.29	5.30	5.29	0.050
hsa-miR-9-star_st	-0.019	2.62E-01	0.837	5.45	5.47	5.46	0.083
hsa-miR-17_st	-0.180	2.63E-01	0.837	11.65	11.83	11.74	0.795
hp_hsa-mir-548f-5_st	-0.012	2.63E-01	0.837	5.35	5.36	5.35	0.050
hp_hsa-mir-153-1_st	0.012	2.63E-01	0.837	5.33	5.32	5.33	0.052
hp_hsa-mir-9-2_st	-0.012	2.63E-01	0.837	5.26	5.27	5.26	0.054
hp_hsa-mir-92a-1_st	0.018	2.64E-01	0.838	5.42	5.40	5.41	0.081

hsa-miR-718_st	-0.025	2.65E-01	0.838	5.59	5.61	5.60	0.113
hsa-miR-920_st	-0.028	2.65E-01	0.838	5.76	5.78	5.77	0.123
hsa-miR-543_st	0.025	2.65E-01	0.838	5.48	5.45	5.47	0.111
hsa-miR-744-star_st	-0.015	2.66E-01	0.838	5.39	5.40	5.39	0.066
hp_hsa-let-7b_x_st	0.016	2.66E-01	0.838	6.30	6.29	6.30	0.069
hsa-miR-500-star_st	-0.081	2.66E-01	0.838	7.30	7.38	7.34	0.358
hsa-miR-1293_st	0.015	2.67E-01	0.838	5.50	5.49	5.50	0.065
hsa-miR-26a_st	0.092	2.67E-01	0.838	12.97	12.87	12.92	0.412
hsa-miR-214-star_st	0.094	2.68E-01	0.838	6.39	6.29	6.34	0.420
hsa-miR-1297_st	0.014	2.68E-01	0.838	5.32	5.31	5.32	0.062
hp_hsa-mir-548f-2_st	0.012	2.68E-01	0.838	5.41	5.40	5.41	0.053
hsa-miR-15b_st	0.132	2.69E-01	0.838	8.36	8.23	8.30	0.589
hp_hsa-mir-3194_st	-0.035	2.69E-01	0.839	6.15	6.18	6.16	0.157
hp_hsa-mir-147b_x_st	-0.011	2.70E-01	0.841	5.35	5.37	5.36	0.049
hsa-miR-519e-star_st	-0.017	2.71E-01	0.842	5.41	5.43	5.42	0.074
hp_hsa-mir-1302-8_st	-0.012	2.72E-01	0.844	5.34	5.35	5.34	0.053
hp_hsa-mir-558_st	0.010	2.72E-01	0.844	5.33	5.32	5.33	0.045
hp_hsa-mir-4291_st	0.026	2.75E-01	0.848	5.86	5.83	5.85	0.119
hsa-miR-1257_st	-0.016	2.75E-01	0.848	5.39	5.40	5.40	0.070
hsa-miR-1226_st	-0.017	2.75E-01	0.848	5.45	5.46	5.45	0.074
hsa-miR-1322_st	0.012	2.76E-01	0.848	5.33	5.32	5.32	0.055
hp_hsa-mir-3180-1_s_st	-0.067	2.76E-01	0.848	6.89	6.96	6.92	0.301
hp_hsa-mir-215_st	-0.012	2.76E-01	0.849	5.35	5.37	5.36	0.052
hp_hsa-mir-548q_x_st	-0.031	2.78E-01	0.850	5.71	5.74	5.72	0.140
hp_hsa-mir-302e_st	-0.011	2.78E-01	0.850	5.31	5.32	5.32	0.051
hp_hsa-mir-1253_st	0.012	2.79E-01	0.850	5.41	5.40	5.41	0.052
hp_hsa-mir-431_st	-0.013	2.79E-01	0.850	5.34	5.35	5.35	0.059
hp_hsa-mir-3074_s_st	-0.008	2.79E-01	0.850	5.33	5.34	5.34	0.037
hp_hsa-mir-514-2_s_st	0.010	2.80E-01	0.852	5.34	5.33	5.34	0.045
hp_hsa-mir-1914_st	-0.037	2.81E-01	0.854	6.03	6.07	6.05	0.171
hp_hsa-mir-23b_st	0.023	2.81E-01	0.854	5.85	5.82	5.83	0.105
hp_hsa-mir-18a_x_st	-0.047	2.83E-01	0.858	5.86	5.91	5.88	0.218
hp_hsa-mir-636_st	-0.026	2.83E-01	0.858	5.67	5.70	5.68	0.119
hsa-miR-181a-star_st	0.037	2.84E-01	0.858	5.68	5.64	5.66	0.170
hsa-miR-526a_st	-0.015	2.85E-01	0.862	5.36	5.37	5.36	0.069
hp_hsa-mir-4282_st	0.012	2.86E-01	0.862	5.44	5.42	5.43	0.056
hp_hsa-mir-136_st	-0.009	2.87E-01	0.863	5.30	5.31	5.30	0.043
hsa-miR-1193_st	-0.035	2.87E-01	0.863	5.84	5.88	5.86	0.164
hp_hsa-mir-1908_st	-0.038	2.88E-01	0.863	6.77	6.81	6.79	0.175
hp_hsa-mir-760_st	-0.023	2.88E-01	0.863	5.76	5.79	5.77	0.109
hp_hsa-mir-2276_st	-0.014	2.89E-01	0.863	5.60	5.61	5.61	0.062
hp_hsa-mir-3159_st	-0.054	2.91E-01	0.863	6.60	6.65	6.63	0.251
hsa-miR-220b_st	-0.013	2.91E-01	0.863	5.39	5.40	5.40	0.062
hsa-miR-96-star_st	0.011	2.91E-01	0.863	5.36	5.35	5.35	0.051
hp_hsa-mir-105-2_s_st	-0.017	2.92E-01	0.863	5.36	5.38	5.37	0.078
hp_hsa-mir-888_st	-0.012	2.92E-01	0.863	5.29	5.30	5.30	0.055
hsa-miR-28-5p_st	0.101	2.93E-01	0.863	8.71	8.61	8.66	0.473

hsa-miR-202-star_st	-0.026	2.94E-01	0.863	5.52	5.54	5.53	0.121
hsa-miR-544b_st	0.011	2.94E-01	0.863	5.30	5.29	5.30	0.051
hsa-miR-520g_st	0.012	2.94E-01	0.863	5.32	5.31	5.32	0.056
hp_hsa-mir-181b-1_st	0.012	2.95E-01	0.863	5.43	5.42	5.42	0.057
hp_hsa-mir-323_st	-0.018	2.95E-01	0.863	5.52	5.54	5.53	0.082
hsa-miR-3184_st	-0.022	2.95E-01	0.863	5.46	5.48	5.47	0.105
hsa-miR-708_st	0.120	2.95E-01	0.863	7.73	7.61	7.67	0.565
hsa-miR-517b_st	0.011	2.96E-01	0.863	5.31	5.29	5.30	0.050
hp_hsa-mir-372_st	-0.014	2.96E-01	0.863	5.38	5.39	5.38	0.068
hsa-miR-190_st	-0.012	2.96E-01	0.863	5.30	5.31	5.31	0.057
hsa-miR-891a_st	-0.013	2.96E-01	0.863	5.40	5.41	5.41	0.062
hp_hsa-mir-4316_st	0.018	2.97E-01	0.863	5.56	5.54	5.55	0.084
hsa-miR-301b_st	0.011	2.97E-01	0.863	5.29	5.28	5.29	0.053
hsa-miR-4310_st	0.037	2.97E-01	0.863	5.82	5.78	5.80	0.175
hp_hsa-mir-1292_st	0.017	2.97E-01	0.863	5.55	5.54	5.54	0.078
hp_hsa-let-7f-2_st	-0.011	2.97E-01	0.863	5.34	5.35	5.34	0.050
hsa-miR-30d_st	0.102	2.97E-01	0.863	8.11	8.00	8.06	0.485
hp_hsa-mir-564_st	-0.037	2.98E-01	0.863	6.18	6.22	6.20	0.176
hsa-miR-601_st	0.021	2.98E-01	0.863	5.36	5.34	5.35	0.099
hsa-miR-23b_st	0.118	2.98E-01	0.863	12.66	12.54	12.60	0.560
hsa-miR-4318_st	0.013	2.99E-01	0.863	5.38	5.37	5.37	0.059
hsa-miR-103_st	-0.078	3.00E-01	0.865	12.35	12.43	12.39	0.371
hp_hsa-mir-147_x_st	0.013	3.00E-01	0.865	5.43	5.41	5.42	0.062
hsa-miR-1184_st	0.061	3.01E-01	0.865	6.31	6.24	6.28	0.293
hp_hsa-mir-1271_st	0.015	3.01E-01	0.865	5.53	5.51	5.52	0.072
hp_hsa-mir-548s_st	0.011	3.01E-01	0.865	5.41	5.40	5.41	0.053
hsa-miR-520e_st	0.011	3.02E-01	0.865	5.32	5.30	5.31	0.052
hp_hsa-mir-1288_st	0.012	3.02E-01	0.866	5.37	5.36	5.37	0.055
hp_hsa-mir-1289-1_st	-0.030	3.04E-01	0.866	5.80	5.83	5.81	0.142
hsa-miR-4251_st	0.012	3.04E-01	0.866	5.35	5.34	5.35	0.058
hsa-miR-148b_st	0.047	3.04E-01	0.866	5.68	5.63	5.65	0.226
hp_hsa-mir-4255_st	-0.012	3.04E-01	0.866	5.37	5.38	5.37	0.055
hp_hsa-mir-4288_st	-0.010	3.05E-01	0.866	5.37	5.38	5.37	0.049
hsa-miR-512-5p_st	-0.012	3.05E-01	0.866	5.33	5.34	5.34	0.059
hsa-miR-186_st	-0.014	3.05E-01	0.866	5.32	5.34	5.33	0.065
hp_hsa-mir-3180-3_s_st	-0.062	3.05E-01	0.866	6.76	6.82	6.79	0.300
hp_hsa-mir-16-1_st	-0.014	3.07E-01	0.869	5.33	5.34	5.33	0.065
hsa-miR-525-5p_st	-0.012	3.07E-01	0.869	5.35	5.36	5.36	0.059
hsa-miR-576-3p_st	0.011	3.08E-01	0.870	5.36	5.35	5.35	0.052
hsa-miR-561_st	0.016	3.09E-01	0.870	5.44	5.43	5.44	0.078
hsa-miR-548c-5p_st	0.013	3.09E-01	0.870	5.44	5.43	5.43	0.064
hsa-miR-4329_st	0.022	3.09E-01	0.870	5.52	5.49	5.50	0.106
hp_hsa-mir-516b-1_x_st	0.011	3.10E-01	0.872	5.38	5.36	5.37	0.052
hsa-miR-125b-2-star_st	0.063	3.11E-01	0.873	5.85	5.79	5.82	0.306
hsa-miR-3115_st	-0.013	3.11E-01	0.873	5.35	5.37	5.36	0.061
hsa-miR-488-star_st	-0.017	3.12E-01	0.873	5.50	5.51	5.50	0.081
hp_hsa-mir-1281_st	-0.033	3.13E-01	0.873	5.85	5.88	5.86	0.163

hp_hsa-mir-296_st	0.018	3.13E-01	0.873	5.61	5.60	5.61	0.086
hsa-miR-596_st	-0.031	3.13E-01	0.873	5.56	5.60	5.58	0.150
hp_hsa-mir-519b_x_st	0.010	3.13E-01	0.873	5.39	5.38	5.38	0.050
hp_hsa-mir-600_st	-0.013	3.14E-01	0.873	5.40	5.41	5.40	0.065
hsa-miR-224-star_st	0.099	3.14E-01	0.873	6.71	6.62	6.67	0.486
hp_hsa-mir-34a_st	-0.021	3.14E-01	0.873	5.79	5.81	5.80	0.102
hp_hsa-mir-1244-1_s_st	0.009	3.15E-01	0.873	5.37	5.36	5.36	0.045
hsa-miR-422a_st	-0.118	3.15E-01	0.873	7.64	7.76	7.70	0.578
hsa-miR-449b-star_st	-0.031	3.16E-01	0.873	5.74	5.77	5.76	0.154
hp_hsa-mir-662_st	0.018	3.16E-01	0.873	5.65	5.63	5.64	0.087
hp_hsa-mir-3119-2_s_st	-0.013	3.18E-01	0.873	5.35	5.36	5.36	0.066
hsa-miR-608_st	0.018	3.18E-01	0.873	5.48	5.46	5.47	0.089
hp_hsa-mir-4300_st	0.012	3.19E-01	0.873	5.54	5.53	5.54	0.059
hp_hsa-mir-423_s_st	-0.046	3.19E-01	0.873	7.47	7.51	7.49	0.228
hp_hsa-mir-937_st	-0.032	3.19E-01	0.873	6.07	6.10	6.08	0.159
hsa-miR-4276_st	0.010	3.19E-01	0.873	5.34	5.33	5.34	0.050
hp_hsa-mir-3200_st	-0.016	3.20E-01	0.873	5.46	5.48	5.47	0.078
hsa-miR-628-3p_st	0.029	3.20E-01	0.873	5.76	5.73	5.75	0.144
hsa-let-7a-star_st	0.011	3.20E-01	0.873	5.37	5.36	5.37	0.055
hp_hsa-mir-96_st	-0.011	3.20E-01	0.873	5.34	5.35	5.35	0.053
hp_hsa-mir-587_x_st	0.010	3.20E-01	0.873	5.45	5.44	5.44	0.048
hp_hsa-mir-520d_x_st	0.012	3.21E-01	0.873	5.37	5.35	5.36	0.058
hp_hsa-mir-518a-1_x_st	-0.012	3.21E-01	0.873	5.38	5.39	5.38	0.060
hp_hsa-mir-146a_st	0.015	3.22E-01	0.874	5.48	5.46	5.47	0.075
hp_hsa-mir-181a-1_st	-0.010	3.22E-01	0.874	5.32	5.33	5.32	0.048
hsa-miR-4320_st	0.010	3.23E-01	0.874	5.34	5.33	5.34	0.050
hsa-miR-590-3p_st	-0.011	3.24E-01	0.876	5.28	5.29	5.29	0.056
hp_hsa-mir-556_st	-0.015	3.25E-01	0.876	5.43	5.45	5.44	0.073
hp_hsa-mir-3118-2_x_st	0.022	3.25E-01	0.876	5.32	5.30	5.31	0.110
hp_hsa-mir-1295_st	-0.014	3.26E-01	0.876	5.38	5.39	5.38	0.071
hp_hsa-mir-3151_st	-0.013	3.27E-01	0.876	5.47	5.48	5.47	0.066
hp_hsa-mir-892a_x_st	-0.010	3.27E-01	0.876	5.44	5.45	5.44	0.050
hp_hsa-mir-1827_x_st	-0.013	3.27E-01	0.876	5.42	5.43	5.42	0.067
hp_hsa-mir-650_st	-0.033	3.27E-01	0.876	5.68	5.71	5.69	0.165
hsa-miR-585_st	0.010	3.28E-01	0.876	5.32	5.31	5.31	0.052
hp_hsa-mir-124-1_st	-0.012	3.28E-01	0.876	5.37	5.38	5.37	0.059
hsa-miR-1912_st	-0.010	3.29E-01	0.876	5.32	5.33	5.32	0.052
hsa-miR-339-3p_st	-0.082	3.29E-01	0.876	7.32	7.40	7.36	0.415
hsa-miR-1202_st	-0.178	3.29E-01	0.876	7.79	7.97	7.88	0.902
hsa-miR-539_st	0.011	3.29E-01	0.876	5.37	5.36	5.37	0.054
hp_hsa-let-7c_st	0.012	3.29E-01	0.876	5.37	5.35	5.36	0.059
hp_hsa-mir-3142_x_st	-0.010	3.30E-01	0.876	5.42	5.43	5.42	0.050
hp_hsa-mir-3139_st	-0.012	3.30E-01	0.876	5.42	5.43	5.42	0.059
hsa-miR-598_st	0.011	3.31E-01	0.876	5.32	5.31	5.31	0.056
hsa-miR-519e_st	0.012	3.32E-01	0.878	5.35	5.34	5.35	0.059
hp_hsa-mir-509-1_s_st	0.022	3.33E-01	0.878	5.38	5.36	5.37	0.112
hsa-miR-527_st	-0.011	3.33E-01	0.878	5.35	5.36	5.36	0.055

hsa-miR-4312_st	-0.020	3.34E-01	0.878	5.60	5.62	5.61	0.100
hp_hsa-mir-3193_st	-0.015	3.34E-01	0.878	5.49	5.50	5.50	0.075
hsa-miR-323-3p_st	0.011	3.34E-01	0.878	5.43	5.42	5.43	0.056
hp_hsa-mir-1283-2_x_st	-0.010	3.35E-01	0.878	5.35	5.36	5.36	0.052
hp_hsa-mir-509-2_s_st	0.022	3.35E-01	0.878	5.43	5.41	5.42	0.114
hp_hsa-mir-548a-3_st	-0.014	3.35E-01	0.878	5.42	5.44	5.43	0.069
hp_hsa-mir-371_st	-0.020	3.35E-01	0.878	5.53	5.55	5.54	0.104
hp_hsa-mir-514b_st	0.013	3.36E-01	0.878	5.48	5.47	5.47	0.065
hp_hsa-mir-935_st	-0.049	3.37E-01	0.880	5.99	6.04	6.01	0.252
hsa-miR-615-3p_st	0.040	3.37E-01	0.880	5.79	5.75	5.77	0.205
hp_hsa-mir-589_st	-0.013	3.38E-01	0.880	5.46	5.47	5.46	0.067
hp_hsa-mir-588_st	0.010	3.38E-01	0.880	5.35	5.34	5.35	0.051
hsa-miR-129-star_st	-0.016	3.38E-01	0.880	5.47	5.48	5.48	0.083
hp_hsa-mir-579_x_st	-0.010	3.39E-01	0.881	5.34	5.35	5.34	0.051
hsa-miR-525-3p_st	0.011	3.40E-01	0.882	5.31	5.30	5.31	0.054
hsa-miR-149-star_st	-0.108	3.40E-01	0.882	12.19	12.30	12.25	0.559
hsa-miR-423-5p_st	-0.091	3.41E-01	0.882	9.06	9.15	9.10	0.471
hsa-miR-548g_st	-0.011	3.41E-01	0.882	5.29	5.30	5.29	0.057
hsa-miR-181c-star_st	-0.035	3.42E-01	0.882	5.65	5.69	5.67	0.182
hp_hsa-mir-126_st	0.022	3.42E-01	0.882	6.17	6.15	6.16	0.116
hsa-miR-1909-star_st	-0.073	3.43E-01	0.883	6.33	6.41	6.37	0.381
hsa-miR-146a-star_st	0.010	3.45E-01	0.887	5.31	5.30	5.31	0.052
hsa-miR-646_st	0.013	3.46E-01	0.888	5.36	5.35	5.36	0.067
hsa-let-7e-star_st	0.015	3.46E-01	0.888	5.40	5.38	5.39	0.079
hp_hsa-mir-3117_st	-0.025	3.46E-01	0.888	5.58	5.61	5.60	0.130
hp_hsa-mir-523_x_st	0.009	3.47E-01	0.888	5.30	5.29	5.30	0.049
hp_hsa-mir-495_st	0.010	3.48E-01	0.888	5.32	5.31	5.32	0.052
hp_hsa-mir-1302-6_st	-0.011	3.49E-01	0.888	5.31	5.32	5.32	0.058
hp_hsa-mir-3134_st	-0.010	3.49E-01	0.888	5.32	5.33	5.32	0.051
hp_hsa-mir-186_st	0.008	3.51E-01	0.888	5.32	5.31	5.32	0.042
hsa-miR-32_st	-0.025	3.51E-01	0.888	5.52	5.55	5.53	0.129
hp_hsa-mir-1225_st	-0.018	3.52E-01	0.888	5.61	5.63	5.62	0.095
hp_hsa-mir-320d-1_x_st	0.018	3.52E-01	0.888	5.52	5.50	5.51	0.095
hp_hsa-mir-1324_st	-0.014	3.52E-01	0.888	5.46	5.47	5.46	0.072
hp_hsa-mir-627_st	-0.011	3.52E-01	0.888	5.38	5.40	5.39	0.059
hp_hsa-mir-526b_x_st	-0.014	3.53E-01	0.888	5.46	5.47	5.46	0.073
hp_hsa-mir-19b-2_st	0.013	3.53E-01	0.888	5.42	5.41	5.41	0.070
hsa-miR-619_st	-0.012	3.53E-01	0.888	5.36	5.37	5.37	0.064
hp_hsa-mir-608_st	-0.012	3.53E-01	0.888	5.48	5.49	5.48	0.063
hsa-miR-597_st	0.014	3.53E-01	0.888	5.44	5.43	5.44	0.074
hp_hsa-mir-4251_st	-0.023	3.54E-01	0.888	5.69	5.71	5.70	0.123
hsa-miR-1261_st	-0.013	3.54E-01	0.888	5.37	5.38	5.38	0.067
hp_hsa-mir-1305_st	-0.016	3.55E-01	0.888	5.50	5.52	5.51	0.085
hp_hsa-mir-744_st	-0.017	3.55E-01	0.888	5.61	5.63	5.62	0.089
hp_hsa-mir-1207_st	-0.016	3.55E-01	0.888	5.57	5.58	5.58	0.087
hp_hsa-mir-599_st	0.012	3.56E-01	0.888	5.43	5.42	5.42	0.066
hp_hsa-mir-3187_st	0.017	3.56E-01	0.888	5.59	5.57	5.58	0.090

hp_hsa-mir-4315-1_s_st	-0.022	3.56E-01	0.888	5.75	5.77	5.76	0.118
hp_hsa-mir-3191_s_st	-0.022	3.56E-01	0.888	5.78	5.81	5.80	0.120
hsa-miR-548h_st	-0.011	3.57E-01	0.888	5.38	5.39	5.38	0.057
hp_hsa-mir-4266_st	-0.014	3.57E-01	0.888	5.46	5.47	5.46	0.073
hsa-miR-4264_st	-0.011	3.58E-01	0.888	5.31	5.32	5.32	0.059
hsa-miR-122-star_st	-0.013	3.58E-01	0.888	5.36	5.37	5.36	0.070
hp_hsa-mir-607_st	0.021	3.58E-01	0.888	5.88	5.86	5.87	0.111
hp_hsa-mir-3130-3_s_st	0.011	3.59E-01	0.888	5.42	5.41	5.42	0.057
hsa-miR-30d-star_st	-0.012	3.59E-01	0.888	5.31	5.32	5.31	0.063
hsa-miR-1266_st	-0.017	3.59E-01	0.888	5.62	5.64	5.63	0.090
hp_hsa-mir-3142_st	-0.011	3.60E-01	0.888	5.34	5.35	5.35	0.059
hp_hsa-mir-653_st	-0.010	3.60E-01	0.888	5.33	5.34	5.33	0.054
hp_hsa-mir-2053_st	-0.009	3.60E-01	0.888	5.30	5.31	5.31	0.045
hp_hsa-mir-3118-5_x_st	-0.012	3.61E-01	0.888	5.30	5.31	5.31	0.065
hsa-miR-574-5p_st	0.066	3.63E-01	0.888	7.12	7.05	7.09	0.357
hp_hsa-mir-1267_st	-0.011	3.63E-01	0.888	5.43	5.44	5.43	0.057
hsa-miR-503_st	0.094	3.63E-01	0.888	6.59	6.50	6.55	0.510
hsa-miR-1249_st	-0.018	3.64E-01	0.888	5.57	5.59	5.58	0.095
hsa-miR-1286_st	-0.012	3.65E-01	0.888	5.39	5.40	5.39	0.063
hp_hsa-mir-874_st	0.023	3.65E-01	0.888	5.99	5.97	5.98	0.124
hsa-miR-4311_st	-0.021	3.66E-01	0.888	5.46	5.48	5.47	0.115
hsa-miR-196a_st	-0.146	3.66E-01	0.888	7.08	7.22	7.15	0.799
hp_hsa-mir-570_st	0.014	3.66E-01	0.888	5.46	5.44	5.45	0.076
hp_hsa-mir-571_st	0.010	3.67E-01	0.888	5.43	5.42	5.43	0.056
hp_hsa-mir-518f_x_st	-0.012	3.68E-01	0.888	5.35	5.36	5.35	0.067
hsa-miR-3165_st	-0.012	3.68E-01	0.888	5.33	5.34	5.33	0.064
hsa-miR-16-1-star_st	-0.010	3.69E-01	0.888	5.34	5.35	5.35	0.057
hsa-miR-328_st	0.029	3.69E-01	0.888	5.74	5.71	5.73	0.158
hp_hsa-mir-4278_st	-0.010	3.69E-01	0.888	5.40	5.41	5.40	0.055
hsa-miR-3130-3p_st	-0.015	3.69E-01	0.888	5.51	5.52	5.51	0.080
hp_hsa-mir-194-1_x_st	-0.016	3.69E-01	0.888	5.91	5.93	5.92	0.089
hp_hsa-mir-29b-1_x_st	0.011	3.70E-01	0.888	5.36	5.35	5.35	0.060
hp_hsa-mir-2277_st	-0.018	3.71E-01	0.888	5.70	5.71	5.71	0.100
hp_hsa-mir-129-2_st	0.011	3.71E-01	0.888	5.50	5.49	5.49	0.062
hp_hsa-mir-16-2_st	-0.020	3.71E-01	0.888	5.56	5.58	5.57	0.110
hp_hsa-mir-210_st	-0.037	3.71E-01	0.888	6.40	6.43	6.41	0.201
hp_hsa-mir-542_st	0.013	3.72E-01	0.888	5.49	5.47	5.48	0.070
hp_hsa-mir-1231_st	-0.016	3.72E-01	0.888	5.75	5.77	5.76	0.088
hsa-miR-424_st	-0.010	3.73E-01	0.888	5.27	5.28	5.28	0.055
hp_hsa-mir-130a_st	0.013	3.73E-01	0.888	5.53	5.52	5.52	0.072
hp_hsa-mir-3118-6_x_st	-0.011	3.73E-01	0.888	5.41	5.42	5.42	0.060
hsa-miR-346_st	-0.067	3.74E-01	0.888	6.45	6.51	6.48	0.369
hsa-miR-3200_st	-0.038	3.74E-01	0.888	5.54	5.58	5.56	0.208
hsa-miR-2909_st	-0.010	3.74E-01	0.888	5.39	5.41	5.40	0.057
hp_hsa-mir-579_st	0.010	3.74E-01	0.888	5.41	5.40	5.41	0.055
hsa-miR-2114-star_st	-0.011	3.75E-01	0.888	5.33	5.34	5.34	0.063
hp_hsa-mir-16-1_x_st	0.012	3.75E-01	0.888	5.81	5.80	5.80	0.065

hp_hsa-mir-501_st	-0.021	3.76E-01	0.888	5.61	5.64	5.62	0.116
hsa-miR-27b_st	0.127	3.77E-01	0.888	9.59	9.46	9.53	0.707
hsa-miR-654-5p_st	0.022	3.77E-01	0.888	5.62	5.60	5.61	0.120
hsa-miR-4274_st	0.024	3.77E-01	0.888	5.77	5.74	5.76	0.133
hp_hsa-mir-559_st	-0.013	3.77E-01	0.888	5.57	5.58	5.58	0.071
hsa-miR-34b-star_st	-0.014	3.77E-01	0.888	5.45	5.46	5.45	0.077
hsa-miR-634_st	0.012	3.77E-01	0.888	5.41	5.40	5.41	0.065
hp_hsa-mir-30e_x_st	-0.045	3.78E-01	0.888	5.98	6.02	6.00	0.251
hsa-miR-520h_st	0.010	3.78E-01	0.888	5.32	5.31	5.32	0.057
hsa-miR-519a_st	-0.011	3.78E-01	0.888	5.27	5.28	5.28	0.059
hp_hsa-mir-661_st	-0.020	3.79E-01	0.888	5.81	5.83	5.82	0.114
hp_hsa-mir-143_st	0.053	3.79E-01	0.888	6.58	6.52	6.55	0.295
hsa-miR-593-star_st	0.012	3.79E-01	0.888	5.39	5.38	5.39	0.064
hsa-miR-143-star_st	0.122	3.80E-01	0.888	7.44	7.31	7.38	0.684
hsa-miR-518b_st	0.010	3.80E-01	0.888	5.36	5.35	5.36	0.054
hp_hsa-mir-373_st	0.011	3.80E-01	0.888	5.38	5.37	5.37	0.064
hp_hsa-mir-513b_x_st	-0.011	3.81E-01	0.890	5.41	5.42	5.41	0.063
hsa-miR-3182_st	-0.011	3.82E-01	0.890	5.25	5.26	5.26	0.061
hp_hsa-mir-887_st	-0.015	3.83E-01	0.890	5.59	5.60	5.59	0.083
hp_hsa-mir-518e_st	0.010	3.83E-01	0.890	5.32	5.31	5.31	0.054
hp_hsa-mir-4304_st	-0.021	3.83E-01	0.890	5.57	5.59	5.58	0.118
hp_hsa-mir-3126_st	-0.010	3.84E-01	0.890	5.40	5.41	5.40	0.057
hp_hsa-mir-548i-1_x_st	0.009	3.84E-01	0.890	5.39	5.38	5.39	0.049
hp_hsa-mir-106a_st	-0.009	3.84E-01	0.890	5.29	5.30	5.29	0.049
hsa-miR-34c-3p_st	0.022	3.86E-01	0.893	5.62	5.60	5.61	0.124
hp_hsa-mir-141_st	-0.019	3.86E-01	0.893	5.60	5.62	5.61	0.111
hsa-miR-423-3p_st	-0.075	3.87E-01	0.893	8.52	8.59	8.55	0.428
hp_hsa-mir-892b_st	-0.010	3.88E-01	0.893	5.36	5.37	5.37	0.059
hp_hsa-mir-539_st	0.012	3.88E-01	0.893	5.38	5.37	5.38	0.068
hsa-miR-218-1-star_st	0.011	3.89E-01	0.893	5.28	5.27	5.28	0.060
hp_hsa-mir-214_st	0.016	3.89E-01	0.893	5.68	5.67	5.68	0.090
hsa-miR-1183_st	0.040	3.90E-01	0.893	5.99	5.95	5.97	0.228
hp_hsa-mir-29c_st	0.012	3.90E-01	0.893	5.34	5.33	5.33	0.068
hsa-miR-891b_st	-0.013	3.90E-01	0.893	5.49	5.50	5.50	0.075
hsa-let-7b_st	0.040	3.90E-01	0.893	14.12	14.08	14.10	0.232
hsa-miR-4323_st	-0.011	3.90E-01	0.893	5.41	5.42	5.41	0.062
hp_hsa-mir-4276_st	0.009	3.92E-01	0.895	5.48	5.47	5.48	0.054
hp_hsa-mir-1294_st	-0.011	3.92E-01	0.895	5.39	5.41	5.40	0.064
hp_hsa-mir-3153_st	0.020	3.93E-01	0.895	5.69	5.67	5.68	0.116
hsa-miR-221-star_st	0.025	3.94E-01	0.897	5.62	5.59	5.61	0.147
hp_hsa-mir-548m_x_st	0.010	3.96E-01	0.897	5.41	5.40	5.40	0.058
hsa-miR-1915_st	-0.084	3.96E-01	0.897	12.95	13.03	12.99	0.491
hp_hsa-mir-548x_st	0.014	3.97E-01	0.897	5.49	5.47	5.48	0.081
hp_hsa-mir-3116-2_s_st	0.010	3.97E-01	0.897	5.36	5.35	5.35	0.057
hsa-miR-3188_st	0.095	3.97E-01	0.897	7.45	7.35	7.40	0.554
hsa-miR-498_st	-0.046	3.97E-01	0.897	5.95	6.00	5.98	0.267
hp_hsa-mir-24-2_x_st	0.023	3.97E-01	0.897	6.93	6.91	6.92	0.135

hsa-miR-759_st	0.011	3.97E-01	0.897	5.32	5.31	5.31	0.061
hsa-miR-4263_st	0.011	3.97E-01	0.897	5.33	5.32	5.33	0.062
hp_hsa-mir-514-3_s_st	-0.010	3.98E-01	0.898	5.45	5.46	5.46	0.055
hp_hsa-mir-30b_st	0.009	3.99E-01	0.898	5.46	5.45	5.46	0.053
hp_hsa-mir-450b_st	-0.010	3.99E-01	0.898	5.41	5.42	5.42	0.059
hsa-miR-132-star_st	0.014	3.99E-01	0.898	5.32	5.31	5.32	0.083
hp_hsa-mir-585_st	0.011	4.00E-01	0.898	5.40	5.39	5.40	0.064
hp_hsa-mir-3198_st	0.013	4.01E-01	0.898	5.51	5.50	5.50	0.075
hp_hsa-mir-601_st	0.011	4.01E-01	0.898	5.46	5.45	5.45	0.063
hsa-miR-3132_st	0.016	4.01E-01	0.898	5.50	5.49	5.50	0.091
hsa-miR-1203_st	0.011	4.02E-01	0.900	5.47	5.46	5.46	0.061
hp_hsa-mir-572_st	-0.070	4.03E-01	0.900	6.91	6.98	6.94	0.411
hsa-miR-1324_st	0.009	4.04E-01	0.900	5.31	5.30	5.31	0.053
hsa-miR-582-3p_st	-0.010	4.04E-01	0.900	5.33	5.34	5.33	0.057
hp_hsa-mir-1321_st	0.010	4.04E-01	0.900	5.56	5.55	5.55	0.060
hsa-miR-1179_st	0.010	4.05E-01	0.900	5.35	5.34	5.35	0.060
hp_hsa-mir-1278_st	-0.011	4.05E-01	0.901	5.46	5.47	5.46	0.064
hsa-miR-1272_st	-0.033	4.05E-01	0.901	5.67	5.70	5.69	0.195
hsa-miR-4292_st	-0.027	4.06E-01	0.901	5.77	5.80	5.78	0.158
hp_hsa-mir-4265_st	-0.008	4.07E-01	0.902	5.39	5.40	5.39	0.048
hp_hsa-mir-526a-2_x_st	0.009	4.07E-01	0.902	5.39	5.38	5.39	0.052
hp_hsa-mir-124-2_st	-0.015	4.08E-01	0.903	5.50	5.52	5.51	0.088
hp_hsa-mir-548w_st	0.011	4.09E-01	0.903	5.43	5.42	5.43	0.065
hp_hsa-mir-27a_x_st	0.039	4.09E-01	0.903	6.14	6.10	6.12	0.230
hsa-miR-30e_st	0.049	4.10E-01	0.903	6.10	6.05	6.08	0.294
hp_hsa-mir-549_x_st	0.009	4.11E-01	0.904	5.34	5.33	5.34	0.055
hsa-miR-943_st	-0.046	4.11E-01	0.904	6.01	6.05	6.03	0.275
hp_hsa-mir-1237_st	-0.051	4.12E-01	0.904	6.71	6.76	6.73	0.306
hp_hsa-mir-3129_x_st	0.009	4.12E-01	0.904	5.41	5.40	5.41	0.054
hsa-miR-220c_st	-0.009	4.12E-01	0.904	5.32	5.33	5.32	0.056
hsa-miR-376b_st	0.009	4.12E-01	0.904	5.30	5.29	5.30	0.056
hsa-miR-643_st	0.009	4.13E-01	0.905	5.29	5.28	5.28	0.053
hsa-miR-502-3p_st	-0.060	4.14E-01	0.905	7.47	7.53	7.50	0.365
hsa-miR-628-5p_st	-0.028	4.15E-01	0.905	5.74	5.77	5.76	0.172
hsa-miR-1539_st	-0.012	4.15E-01	0.905	5.50	5.51	5.51	0.073
hsa-miR-323-5p_st	-0.041	4.17E-01	0.905	5.61	5.65	5.63	0.250
hp_hsa-mir-3155_x_st	-0.015	4.17E-01	0.905	5.93	5.95	5.94	0.091
hp_hsa-mir-641_x_st	-0.010	4.17E-01	0.905	5.44	5.45	5.44	0.062
hsa-miR-1298_st	-0.010	4.17E-01	0.905	5.31	5.32	5.32	0.061
hp_hsa-mir-320c-2_st	0.011	4.18E-01	0.905	5.40	5.38	5.39	0.067
hsa-miR-3147_st	0.045	4.18E-01	0.905	6.35	6.31	6.33	0.274
hsa-miR-1256_st	-0.013	4.18E-01	0.905	5.37	5.38	5.37	0.076
hp_hsa-mir-147_st	-0.012	4.18E-01	0.905	5.42	5.43	5.43	0.073
hp_hsa-mir-19b-1_st	-0.009	4.18E-01	0.905	5.29	5.30	5.29	0.054
hsa-miR-542-5p_st	0.031	4.19E-01	0.905	5.77	5.74	5.76	0.192
hsa-miR-1290_st	-0.127	4.19E-01	0.905	6.27	6.39	6.33	0.777
hp_hsa-mir-376a-1_st	0.008	4.19E-01	0.905	5.30	5.30	5.30	0.047

hp_hsa-mir-130b_st	-0.014	4.21E-01	0.907	5.84	5.85	5.85	0.084
hsa-miR-4267_st	0.010	4.21E-01	0.907	5.46	5.45	5.45	0.061
hsa-miR-942_st	-0.009	4.23E-01	0.908	5.26	5.27	5.26	0.052
hp_hsa-let-7a-2_x_st	0.010	4.23E-01	0.908	5.42	5.41	5.41	0.061
hsa-miR-588_st	0.008	4.24E-01	0.908	5.35	5.34	5.35	0.048
hsa-miR-330-3p_st	-0.052	4.24E-01	0.908	6.45	6.50	6.48	0.321
hp_hsa-mir-4283-1_s_st	-0.010	4.24E-01	0.908	5.50	5.51	5.50	0.061
hp_hsa-mir-711_st	-0.034	4.25E-01	0.908	6.83	6.86	6.84	0.207
hp_hsa-mir-938_st	0.008	4.25E-01	0.908	5.35	5.34	5.35	0.050
hsa-miR-1321_st	0.013	4.26E-01	0.908	5.37	5.36	5.36	0.078
hp_hsa-mir-561_st	0.011	4.27E-01	0.908	5.48	5.47	5.47	0.069
hp_hsa-mir-767_st	-0.010	4.27E-01	0.908	5.45	5.46	5.45	0.059
hp_hsa-mir-548a-1_x_st	0.012	4.27E-01	0.908	5.48	5.47	5.47	0.073
hp_hsa-mir-513c_x_st	0.010	4.27E-01	0.908	5.39	5.38	5.38	0.064
hp_hsa-mir-4292_st	0.024	4.27E-01	0.908	5.88	5.86	5.87	0.147
hsa-miR-485-3p_st	0.015	4.28E-01	0.908	5.58	5.57	5.58	0.094
hp_hsa-mir-1972-2_s_st	-0.010	4.28E-01	0.908	5.45	5.46	5.45	0.060
hsa-miR-380_st	-0.008	4.28E-01	0.908	5.31	5.32	5.31	0.050
hp_hsa-mir-4299_st	-0.010	4.29E-01	0.909	5.45	5.46	5.45	0.063
hp_hsa-mir-103-1-as_s_st	0.008	4.29E-01	0.909	5.34	5.33	5.33	0.049
hp_hsa-mir-665_st	0.021	4.30E-01	0.910	6.15	6.13	6.14	0.132
hsa-miR-501-3p_st	-0.053	4.31E-01	0.910	6.82	6.87	6.85	0.331
hp_hsa-mir-208a_st	0.008	4.32E-01	0.910	5.34	5.33	5.34	0.052
hsa-miR-326_st	-0.013	4.32E-01	0.910	5.48	5.49	5.48	0.081
hsa-miR-26a-1-star_st	-0.009	4.32E-01	0.910	5.32	5.33	5.32	0.053
hsa-miR-219-1-3p_st	-0.011	4.32E-01	0.910	5.35	5.37	5.36	0.065
hp_hsa-mir-3153_x_st	-0.020	4.32E-01	0.910	5.67	5.69	5.68	0.128
hsa-miR-661_st	-0.013	4.33E-01	0.910	5.37	5.38	5.37	0.084
hsa-miR-142-3p_st	-0.009	4.34E-01	0.910	5.26	5.27	5.27	0.056
hp_hsa-mir-448_st	-0.017	4.35E-01	0.910	5.85	5.86	5.86	0.109
hp_hsa-mir-320b-1_st	-0.015	4.35E-01	0.910	5.47	5.49	5.48	0.095
hsa-miR-642_st	0.009	4.36E-01	0.910	5.32	5.31	5.32	0.053
hp_hsa-mir-609_st	-0.009	4.36E-01	0.910	5.42	5.43	5.43	0.059
hp_hsa-mir-211_x_st	-0.011	4.37E-01	0.910	5.49	5.51	5.50	0.070
hp_hsa-mir-1-1_st	0.010	4.37E-01	0.910	5.37	5.36	5.37	0.061
hp_hsa-mir-3199-2_s_st	-0.009	4.37E-01	0.910	5.37	5.38	5.38	0.059
hsa-miR-3124_st	0.032	4.38E-01	0.910	6.29	6.26	6.27	0.201
hsa-miR-621_st	-0.009	4.38E-01	0.910	5.34	5.35	5.35	0.060
hsa-miR-187_st	0.048	4.39E-01	0.910	5.57	5.52	5.54	0.303
hsa-miR-548u_st	-0.022	4.39E-01	0.910	5.57	5.59	5.58	0.139
hp_hsa-mir-378b_st	-0.013	4.39E-01	0.910	5.61	5.62	5.61	0.084
hp_hsa-mir-3163_st	0.012	4.40E-01	0.910	5.52	5.51	5.51	0.076
hp_hsa-mir-142_st	-0.013	4.40E-01	0.910	5.42	5.44	5.43	0.081
hsa-miR-154-star_st	0.010	4.40E-01	0.910	5.42	5.41	5.42	0.065
hp_hsa-mir-220a_x_st	-0.013	4.41E-01	0.911	5.49	5.51	5.50	0.082
hp_hsa-mir-133a-2_s_st	0.009	4.42E-01	0.913	5.42	5.42	5.42	0.054
hsa-miR-153_st	0.008	4.42E-01	0.913	5.34	5.33	5.34	0.053

hp_hsa-mir-92a-1_x_st	0.016	4.43E-01	0.914	6.10	6.08	6.09	0.103
hp_hsa-mir-657_st	0.030	4.44E-01	0.915	6.08	6.05	6.07	0.196
hsa-miR-556-3p_st	-0.009	4.45E-01	0.916	5.31	5.32	5.32	0.060
hp_hsa-mir-145_st	0.028	4.45E-01	0.916	6.13	6.10	6.12	0.178
hp_hsa-mir-578_st	-0.009	4.47E-01	0.918	5.45	5.46	5.46	0.060
hp_hsa-mir-3147_st	0.013	4.47E-01	0.918	5.67	5.65	5.66	0.085
hsa-miR-515-5p_st	-0.011	4.47E-01	0.918	5.38	5.39	5.39	0.069
hsa-miR-4296_st	-0.010	4.49E-01	0.920	5.39	5.41	5.40	0.065
hp_hsa-mir-26b_st	0.012	4.50E-01	0.920	5.77	5.76	5.76	0.077
hp_hsa-mir-520e_x_st	-0.007	4.50E-01	0.920	5.33	5.34	5.33	0.044
hp_hsa-mir-502_x_st	-0.016	4.50E-01	0.920	5.72	5.73	5.72	0.102
hp_hsa-mir-20a_st	0.021	4.51E-01	0.920	5.67	5.65	5.66	0.140
hp_hsa-mir-521-1_x_st	0.011	4.51E-01	0.921	5.50	5.49	5.49	0.069
hp_hsa-mir-891b_x_st	-0.010	4.52E-01	0.921	5.40	5.41	5.40	0.067
hsa-miR-605_st	-0.013	4.52E-01	0.921	5.45	5.47	5.46	0.083
hsa-miR-3144-3p_st	-0.012	4.53E-01	0.922	5.40	5.42	5.41	0.077
hsa-miR-4321_st	-0.025	4.54E-01	0.922	5.66	5.69	5.68	0.163
hp_hsa-mir-181c_st	-0.011	4.56E-01	0.925	5.49	5.50	5.49	0.072
hp_hsa-mir-620_st	-0.010	4.56E-01	0.925	5.41	5.42	5.42	0.068
hp_hsa-mir-26a-1_st	0.019	4.57E-01	0.927	5.84	5.82	5.83	0.123
hsa-miR-675-star_st	-0.020	4.58E-01	0.927	5.53	5.55	5.54	0.134
hsa-miR-300_st	-0.010	4.59E-01	0.927	5.37	5.38	5.37	0.068
hsa-miR-106a-star_st	-0.009	4.59E-01	0.927	5.29	5.30	5.29	0.057
hp_hsa-mir-544_st	-0.007	4.60E-01	0.927	5.31	5.32	5.32	0.046
hp_hsa-mir-548p_st	0.007	4.60E-01	0.927	5.30	5.29	5.30	0.047
hsa-miR-490-3p_st	0.034	4.60E-01	0.928	5.49	5.46	5.47	0.230
hsa-miR-30e-star_st	0.022	4.61E-01	0.928	5.52	5.49	5.51	0.148
hsa-miR-371-5p_st	-0.047	4.62E-01	0.928	6.09	6.13	6.11	0.316
hsa-miR-571_st	-0.009	4.62E-01	0.928	5.36	5.36	5.36	0.059
hsa-miR-3120_st	0.008	4.62E-01	0.929	5.37	5.36	5.36	0.055
hp_hsa-mir-892a_st	0.007	4.64E-01	0.931	5.35	5.35	5.35	0.049
hsa-miR-548t_st	-0.008	4.65E-01	0.931	5.36	5.37	5.36	0.057
hp_hsa-mir-449b_st	0.010	4.65E-01	0.931	5.47	5.46	5.47	0.066
hp_hsa-mir-346_st	-0.026	4.65E-01	0.931	5.75	5.78	5.76	0.173
hsa-miR-2861_st	-0.074	4.66E-01	0.931	12.95	13.02	12.98	0.500
hsa-miR-431-star_st	0.010	4.67E-01	0.931	5.40	5.39	5.39	0.065
hsa-miR-4288_st	-0.011	4.67E-01	0.931	5.47	5.48	5.48	0.071
hp_hsa-mir-128-2_st	-0.010	4.68E-01	0.932	5.48	5.49	5.49	0.065
hp_hsa-mir-3145_st	0.008	4.69E-01	0.934	5.41	5.40	5.40	0.053
hp_hsa-mir-220b_x_st	-0.015	4.71E-01	0.936	5.70	5.72	5.71	0.104
hsa-miR-18b_st	-0.057	4.71E-01	0.936	5.92	5.98	5.95	0.387
hsa-miR-1538_st	0.018	4.71E-01	0.936	5.61	5.59	5.60	0.124
hsa-miR-1258_st	-0.007	4.73E-01	0.936	5.31	5.32	5.31	0.046
hsa-miR-302f_st	0.007	4.74E-01	0.936	5.28	5.27	5.27	0.048
hp_hsa-mir-103-2-as_s_st	-0.008	4.74E-01	0.936	5.41	5.42	5.41	0.056
hsa-miR-4306_st	-0.021	4.75E-01	0.936	5.81	5.83	5.82	0.145
hp_hsa-mir-130b_x_st	-0.012	4.75E-01	0.936	5.81	5.82	5.82	0.085

hsa-miR-1294_st	-0.014	4.75E-01	0.936	5.57	5.59	5.58	0.097
hsa-miR-33b_st	0.011	4.76E-01	0.936	5.45	5.44	5.44	0.079
hp_hsa-mir-513b_st	0.008	4.76E-01	0.936	5.33	5.32	5.33	0.057
hp_hsa-mir-545_st	0.008	4.76E-01	0.936	5.39	5.38	5.39	0.057
hp_hsa-mir-1302-5_st	-0.007	4.77E-01	0.936	5.30	5.31	5.30	0.049
hsa-miR-151-3p_st	-0.069	4.77E-01	0.936	8.67	8.74	8.70	0.481
hp_hsa-mir-585_x_st	0.008	4.77E-01	0.936	5.35	5.34	5.35	0.057
hsa-miR-20a_st	-0.120	4.79E-01	0.936	10.90	11.02	10.96	0.837
hp_hsa-mir-1539_st	-0.009	4.79E-01	0.936	5.45	5.46	5.45	0.065
hsa-miR-499-5p_st	0.009	4.79E-01	0.936	5.32	5.32	5.32	0.062
hp_hsa-mir-670_st	-0.008	4.79E-01	0.936	5.39	5.40	5.40	0.056
hp_hsa-mir-543_st	0.008	4.79E-01	0.936	5.41	5.41	5.41	0.053
hp_hsa-mir-18a_st	0.009	4.80E-01	0.936	5.42	5.41	5.42	0.060
hsa-miR-4302_st	0.009	4.80E-01	0.936	5.49	5.48	5.48	0.063
hsa-miR-373_st	0.014	4.80E-01	0.936	5.35	5.34	5.34	0.098
hsa-miR-3162_st	-0.109	4.81E-01	0.936	8.40	8.51	8.46	0.762
hp_hsa-mir-450a-2_x_st	0.007	4.81E-01	0.936	5.33	5.33	5.33	0.051
hsa-miR-885-5p_st	-0.040	4.81E-01	0.936	6.03	6.07	6.05	0.278
hp_hsa-let-7f-2_x_st	0.009	4.82E-01	0.936	5.62	5.61	5.61	0.063
hp_hsa-mir-642_x_st	0.018	4.83E-01	0.936	5.77	5.75	5.76	0.124
hp_hsa-mir-889_st	-0.009	4.83E-01	0.936	5.35	5.35	5.35	0.060
hp_hsa-mir-496_st	0.007	4.83E-01	0.936	5.32	5.32	5.32	0.048
hsa-miR-4268_st	-0.008	4.85E-01	0.936	5.27	5.28	5.27	0.059
hsa-miR-1228_st	-0.032	4.85E-01	0.936	6.24	6.27	6.25	0.225
hp_hsa-mir-3128_x_st	-0.015	4.85E-01	0.936	5.64	5.66	5.65	0.105
hp_hsa-mir-384_st	0.007	4.85E-01	0.936	5.34	5.33	5.34	0.052
hp_hsa-mir-664_s_st	0.023	4.86E-01	0.936	5.84	5.82	5.83	0.164
hsa-miR-616_st	0.013	4.86E-01	0.936	5.43	5.42	5.42	0.094
hp_hsa-mir-1257_st	-0.012	4.86E-01	0.936	5.57	5.58	5.57	0.082
hsa-miR-572_st	-0.127	4.87E-01	0.936	8.38	8.51	8.45	0.898
hsa-miR-940_st	-0.037	4.87E-01	0.936	6.50	6.54	6.52	0.261
hsa-miR-548a-5p_st	0.011	4.88E-01	0.936	5.54	5.53	5.53	0.081
hp_hsa-mir-9-3_x_st	-0.013	4.88E-01	0.936	5.62	5.63	5.63	0.093
hsa-miR-3186-3p_st	0.010	4.89E-01	0.936	5.51	5.50	5.51	0.074
hsa-miR-4313_st	0.012	4.89E-01	0.936	5.63	5.61	5.62	0.083
hsa-miR-580_st	-0.008	4.90E-01	0.936	5.28	5.29	5.28	0.054
hp_hsa-mir-3180-2_s_st	-0.042	4.90E-01	0.936	6.75	6.79	6.77	0.296
hsa-miR-3167_st	0.008	4.90E-01	0.936	5.28	5.28	5.28	0.054
hsa-miR-496_st	0.011	4.90E-01	0.936	5.44	5.43	5.44	0.079
hp_hsa-mir-548d-1_x_st	-0.007	4.91E-01	0.936	5.33	5.34	5.33	0.050
hsa-miR-1254_st	-0.018	4.91E-01	0.936	5.85	5.87	5.86	0.131
hsa-miR-545_st	0.008	4.91E-01	0.936	5.34	5.33	5.34	0.053
hp_hsa-mir-586_x_st	-0.008	4.91E-01	0.936	5.33	5.34	5.33	0.053
hsa-miR-378b_st	0.017	4.92E-01	0.937	5.90	5.89	5.89	0.119
hp_hsa-mir-488_st	0.009	4.93E-01	0.937	5.39	5.38	5.38	0.065
hp_hsa-mir-122_st	0.008	4.93E-01	0.937	5.33	5.32	5.33	0.054
hp_hsa-mir-1471_st	-0.016	4.95E-01	0.938	5.87	5.89	5.88	0.118

hsa-miR-590-5p_st	-0.009	4.95E-01	0.938	5.35	5.36	5.36	0.064
hsa-miR-30c-1-star_st	-0.019	4.95E-01	0.938	5.64	5.66	5.65	0.135
hsa-miR-1827_st	0.010	4.95E-01	0.938	5.40	5.39	5.40	0.075
hsa-miR-24-1-star_st	-0.010	4.96E-01	0.938	5.34	5.35	5.34	0.073
hp_hsa-mir-9-1_x_st	-0.010	4.97E-01	0.939	5.56	5.57	5.57	0.070
hp_hsa-mir-134_st	0.012	4.97E-01	0.939	5.65	5.64	5.64	0.086
hp_hsa-mir-3140_st	-0.008	4.99E-01	0.941	5.36	5.36	5.36	0.055
hp_hsa-mir-3163_x_st	-0.011	5.00E-01	0.941	5.61	5.62	5.62	0.079
hsa-miR-181d_st	0.069	5.00E-01	0.941	6.89	6.82	6.86	0.503
hsa-miR-200b-star_st	-0.076	5.01E-01	0.941	8.61	8.68	8.64	0.556
hp_hsa-mir-3130-1_s_st	-0.007	5.01E-01	0.941	5.30	5.31	5.31	0.052
hsa-miR-302c_st	-0.007	5.02E-01	0.941	5.29	5.30	5.30	0.051
hp_hsa-mir-383_st	0.009	5.02E-01	0.941	5.50	5.49	5.49	0.064
hsa-miR-92b-star_st	-0.084	5.02E-01	0.941	7.94	8.03	7.98	0.614
hsa-miR-3187_st	-0.058	5.02E-01	0.941	6.49	6.55	6.52	0.424
hsa-miR-4330_st	0.007	5.03E-01	0.941	5.32	5.31	5.31	0.051
hsa-miR-629-star_st	-0.019	5.03E-01	0.941	5.72	5.74	5.73	0.142
hp_hsa-mir-516b-2_x_st	0.007	5.04E-01	0.942	5.35	5.35	5.35	0.052
hsa-miR-449a_st	-0.010	5.04E-01	0.942	5.35	5.36	5.36	0.072
hp_hsa-mir-1912_st	0.006	5.05E-01	0.942	5.33	5.32	5.32	0.047
hsa-miR-4277_st	-0.009	5.07E-01	0.942	5.33	5.34	5.33	0.065
hp_hsa-mir-217_st	0.007	5.07E-01	0.942	5.37	5.37	5.37	0.052
hsa-miR-4308_st	-0.007	5.08E-01	0.942	5.25	5.26	5.26	0.048
hp_hsa-mir-15a_st	0.010	5.08E-01	0.942	5.50	5.49	5.50	0.072
hp_hsa-mir-1179_st	-0.007	5.08E-01	0.942	5.39	5.40	5.40	0.054
hp_hsa-mir-27b_st	-0.011	5.08E-01	0.942	5.47	5.48	5.47	0.081
hsa-miR-630_st	0.013	5.08E-01	0.942	5.42	5.41	5.42	0.098
hp_hsa-mir-128-1_st	-0.009	5.09E-01	0.942	5.42	5.43	5.42	0.065
hsa-miR-196b_st	-0.131	5.09E-01	0.942	7.01	7.14	7.07	0.975
hsa-miR-149_st	0.043	5.09E-01	0.942	6.11	6.07	6.09	0.319
hp_hsa-mir-548f-4_x_st	-0.010	5.09E-01	0.942	5.60	5.61	5.61	0.078
hp_hsa-mir-1255a_st	-0.007	5.11E-01	0.942	5.31	5.32	5.31	0.051
hsa-miR-1826_st	-0.045	5.11E-01	0.942	13.71	13.76	13.73	0.339
hp_hsa-mir-1266_st	0.018	5.11E-01	0.942	5.69	5.67	5.68	0.136
hsa-miR-466_st	0.013	5.12E-01	0.942	5.43	5.42	5.42	0.098
hp_hsa-mir-206_st	0.008	5.12E-01	0.942	5.38	5.37	5.37	0.057
hsa-miR-26b-star_st	-0.012	5.12E-01	0.942	5.43	5.45	5.44	0.089
hp_hsa-mir-604_st	-0.007	5.14E-01	0.943	5.41	5.42	5.42	0.053
hsa-miR-650_st	-0.040	5.14E-01	0.943	5.72	5.76	5.74	0.300
hp_hsa-mir-2114_st	-0.010	5.14E-01	0.943	5.46	5.47	5.46	0.075
hsa-miR-602_st	-0.057	5.14E-01	0.943	6.63	6.68	6.65	0.430
hsa-miR-20b_st	0.096	5.14E-01	0.943	8.20	8.10	8.15	0.729
hp_hsa-mir-520f_x_st	0.007	5.15E-01	0.943	5.39	5.38	5.39	0.049
hsa-miR-581_st	-0.007	5.16E-01	0.943	5.24	5.25	5.25	0.051
hsa-miR-1268_st	-0.076	5.16E-01	0.944	10.33	10.41	10.37	0.573
hsa-miR-1304_st	0.008	5.17E-01	0.944	5.37	5.36	5.37	0.063
hsa-miR-3131_st	-0.025	5.17E-01	0.944	6.01	6.03	6.02	0.187

hsa-miR-3183_st	0.008	5.18E-01	0.944	5.32	5.31	5.31	0.060
hp_hsa-mir-3143_st	-0.023	5.18E-01	0.944	5.83	5.85	5.84	0.178
hsa-miR-629_st	-0.042	5.21E-01	0.947	6.55	6.59	6.57	0.324
hp_hsa-mir-548i-1_s_st	-0.008	5.21E-01	0.947	5.50	5.51	5.50	0.062
hp_hsa-mir-374b_st	-0.008	5.21E-01	0.947	5.49	5.50	5.49	0.063
hp_hsa-mir-376b_st	-0.010	5.22E-01	0.947	5.36	5.37	5.37	0.073
hsa-miR-1250_st	0.009	5.22E-01	0.947	5.49	5.49	5.49	0.068
hp_hsa-mir-153-1_x_st	-0.007	5.24E-01	0.949	5.41	5.41	5.41	0.050
hp_hsa-mir-216a_st	-0.006	5.24E-01	0.949	5.40	5.41	5.41	0.049
hp_hsa-mir-3141_st	-0.020	5.25E-01	0.949	6.60	6.62	6.61	0.155
hp_hsa-mir-597_st	-0.008	5.27E-01	0.949	5.38	5.39	5.38	0.060
hsa-miR-32-star_st	0.009	5.27E-01	0.949	5.41	5.40	5.41	0.073
hp_hsa-mir-641_st	0.009	5.27E-01	0.949	5.38	5.37	5.38	0.067
hsa-miR-381_st	0.018	5.27E-01	0.949	5.63	5.61	5.62	0.143
hsa-miR-130a-star_st	0.006	5.27E-01	0.949	5.34	5.34	5.34	0.049
hp_hsa-mir-548f-2_x_st	-0.008	5.27E-01	0.949	5.50	5.51	5.51	0.065
hsa-miR-4325_st	0.008	5.27E-01	0.949	5.39	5.38	5.39	0.065
hp_hsa-mir-1244-3_s_st	-0.008	5.28E-01	0.949	5.33	5.34	5.33	0.059
hsa-miR-584_st	0.061	5.28E-01	0.949	6.25	6.19	6.22	0.474
hp_hsa-mir-875_st	-0.007	5.29E-01	0.949	5.32	5.33	5.33	0.052
hp_hsa-mir-329-2_s_st	0.006	5.30E-01	0.951	5.34	5.34	5.34	0.046
hp_hsa-mir-500b_st	0.013	5.31E-01	0.951	5.58	5.57	5.58	0.102
hp_hsa-mir-449c_st	-0.008	5.31E-01	0.951	5.37	5.37	5.37	0.059
hp_hsa-mir-1184-3_s_st	0.010	5.31E-01	0.951	5.59	5.58	5.59	0.081
hsa-miR-4258_st	-0.015	5.32E-01	0.951	5.65	5.67	5.66	0.115
hp_hsa-mir-149_st	0.008	5.33E-01	0.952	5.59	5.58	5.59	0.065
hp_hsa-mir-1302-11_s_st	0.009	5.33E-01	0.952	5.32	5.31	5.31	0.073
hp_hsa-mir-532_st	-0.015	5.34E-01	0.953	6.07	6.09	6.08	0.116
hp_hsa-mir-17_st	0.012	5.35E-01	0.953	5.52	5.51	5.51	0.095
hsa-miR-4316_st	0.008	5.36E-01	0.954	5.58	5.57	5.58	0.063
hp_hsa-mir-1263_st	-0.013	5.36E-01	0.954	5.64	5.66	5.65	0.106
hsa-miR-191-star_st	-0.037	5.36E-01	0.954	6.19	6.23	6.21	0.298
hsa-miR-20a-star_st	-0.007	5.38E-01	0.956	5.34	5.34	5.34	0.058
hp_hsa-mir-505_x_st	-0.016	5.39E-01	0.956	5.64	5.65	5.65	0.127
hp_hsa-mir-1306_st	0.010	5.39E-01	0.956	5.55	5.54	5.54	0.079
hsa-miR-2052_st	-0.006	5.40E-01	0.956	5.28	5.28	5.28	0.048
hsa-miR-4300_st	0.008	5.40E-01	0.956	5.32	5.31	5.31	0.062
hsa-miR-649_st	-0.007	5.40E-01	0.956	5.28	5.29	5.28	0.055
hsa-miR-340_st	-0.009	5.42E-01	0.957	5.35	5.36	5.36	0.069
hsa-miR-2276_st	0.022	5.44E-01	0.957	5.94	5.91	5.92	0.175
hp_hsa-mir-370_st	0.009	5.44E-01	0.957	5.63	5.62	5.63	0.074
hsa-miR-3140_st	0.007	5.44E-01	0.957	5.35	5.35	5.35	0.057
hp_hsa-mir-548o_st	0.006	5.45E-01	0.957	5.35	5.35	5.35	0.052
hsa-miR-708-star_st	0.007	5.46E-01	0.957	5.34	5.33	5.34	0.054
hsa-miR-224_st	0.074	5.46E-01	0.957	6.55	6.47	6.51	0.607
hsa-miR-454-star_st	-0.006	5.46E-01	0.957	5.30	5.31	5.30	0.051
hp_hsa-mir-4272_st	-0.005	5.46E-01	0.957	5.29	5.30	5.30	0.043

hp_hsa-mir-20b_st	0.012	5.47E-01	0.957	5.58	5.56	5.57	0.097
hsa-miR-30b_st	0.074	5.47E-01	0.957	8.03	7.96	8.00	0.603
hp_hsa-mir-4263_st	-0.009	5.47E-01	0.957	5.54	5.55	5.55	0.074
hp_hsa-mir-4264_st	0.007	5.47E-01	0.957	5.46	5.46	5.46	0.055
hp_hsa-mir-3179-1_s_st	0.009	5.48E-01	0.957	5.48	5.47	5.47	0.074
hsa-miR-340-star_st	-0.007	5.48E-01	0.957	5.29	5.30	5.30	0.058
hsa-miR-541-star_st	0.011	5.48E-01	0.957	5.62	5.61	5.61	0.087
hp_hsa-mir-619_st	-0.006	5.49E-01	0.957	5.31	5.31	5.31	0.046
hp_hsa-mir-30a_x_st	0.010	5.49E-01	0.957	5.54	5.53	5.53	0.081
hsa-miR-1225-3p_st	-0.014	5.50E-01	0.957	5.61	5.62	5.61	0.112
hp_hsa-mir-3167_st	-0.007	5.51E-01	0.957	5.36	5.36	5.36	0.053
hp_hsa-mir-7-1_st	0.007	5.51E-01	0.957	5.36	5.36	5.36	0.057
hsa-miR-497-star_st	0.007	5.51E-01	0.957	5.31	5.30	5.31	0.053
hp_hsa-mir-202_st	-0.010	5.52E-01	0.957	5.53	5.53	5.53	0.079
hp_hsa-mir-367_st	-0.007	5.52E-01	0.957	5.39	5.39	5.39	0.055
hsa-miR-548c-3p_st	0.011	5.53E-01	0.957	5.39	5.38	5.38	0.091
hp_hsa-mir-29a_st	0.012	5.53E-01	0.957	5.58	5.57	5.58	0.099
hsa-miR-518f_st	-0.005	5.53E-01	0.957	5.30	5.30	5.30	0.044
hsa-miR-620_st	0.007	5.54E-01	0.957	5.36	5.35	5.35	0.054
hp_hsa-mir-645_st	-0.008	5.54E-01	0.957	5.55	5.56	5.56	0.063
hsa-miR-658_st	-0.017	5.55E-01	0.957	5.88	5.90	5.89	0.144
hp_hsa-mir-1236_st	0.009	5.57E-01	0.957	5.48	5.47	5.47	0.078
hp_hsa-mir-15b_st	0.010	5.57E-01	0.957	5.73	5.72	5.73	0.080
hp_hsa-mir-607_x_st	0.011	5.57E-01	0.957	5.75	5.74	5.75	0.089
hp_hsa-mir-490_st	-0.007	5.57E-01	0.957	5.40	5.41	5.40	0.057
hp_hsa-mir-1266_x_st	-0.013	5.57E-01	0.957	5.66	5.67	5.67	0.112
hsa-miR-7_st	0.029	5.58E-01	0.957	5.51	5.48	5.50	0.246
hp_hsa-mir-19b-1_x_st	0.022	5.58E-01	0.957	5.90	5.88	5.89	0.187
hsa-miR-4290_st	-0.015	5.59E-01	0.957	5.65	5.67	5.66	0.125
hp_hsa-mir-219-2_st	-0.007	5.59E-01	0.957	5.39	5.40	5.40	0.062
hsa-miR-623_st	-0.019	5.60E-01	0.957	5.69	5.71	5.70	0.158
hp_hsa-mir-3132_st	-0.013	5.60E-01	0.957	5.84	5.85	5.84	0.110
hsa-miR-15a-star_st	-0.010	5.61E-01	0.957	5.44	5.45	5.45	0.084
hsa-miR-644_st	-0.008	5.61E-01	0.957	5.34	5.35	5.35	0.064
hp_hsa-mir-218-2_st	-0.007	5.61E-01	0.957	5.43	5.44	5.43	0.055
hp_hsa-mir-1255b-1_st	0.007	5.61E-01	0.957	5.36	5.35	5.36	0.061
hsa-miR-30c-2-star_st	0.015	5.61E-01	0.957	5.60	5.59	5.60	0.128
hp_hsa-mir-595_st	-0.006	5.62E-01	0.957	5.42	5.43	5.42	0.051
hsa-miR-578_st	-0.008	5.62E-01	0.957	5.36	5.36	5.36	0.065
hp_hsa-mir-1911_st	0.009	5.64E-01	0.957	5.70	5.69	5.70	0.076
hsa-miR-3126-5p_st	0.013	5.65E-01	0.957	5.63	5.62	5.62	0.108
hp_hsa-mir-2861_st	-0.023	5.66E-01	0.957	6.65	6.67	6.66	0.198
hsa-miR-3065-3p_st	0.011	5.66E-01	0.957	5.40	5.39	5.40	0.093
hp_hsa-mir-1184-2_s_st	0.007	5.67E-01	0.957	5.59	5.58	5.58	0.062
hsa-miR-107_st	-0.042	5.67E-01	0.957	11.79	11.84	11.81	0.363
hp_hsa-mir-1468_st	-0.006	5.67E-01	0.957	5.33	5.34	5.33	0.053
hsa-miR-760_st	-0.029	5.69E-01	0.957	5.84	5.87	5.86	0.253

hsa-miR-3145_st	-0.006	5.69E-01	0.957	5.28	5.28	5.28	0.047
hsa-miR-648_st	-0.009	5.69E-01	0.957	5.62	5.62	5.62	0.081
hsa-miR-4305_st	-0.007	5.70E-01	0.957	5.36	5.37	5.37	0.062
hp_hsa-mir-510_st	-0.009	5.71E-01	0.957	5.43	5.44	5.43	0.082
hp_hsa-mir-4315-2_s_st	-0.014	5.71E-01	0.957	5.74	5.75	5.75	0.121
hsa-miR-614_st	0.006	5.71E-01	0.957	5.35	5.34	5.35	0.052
hp_hsa-mir-1265_st	-0.006	5.71E-01	0.957	5.48	5.48	5.48	0.054
hp_hsa-let-7b_st	0.009	5.72E-01	0.957	6.36	6.35	6.36	0.074
hsa-miR-3198_st	-0.013	5.72E-01	0.957	5.68	5.69	5.68	0.111
hp_hsa-mir-1251_st	0.006	5.72E-01	0.957	5.35	5.35	5.35	0.048
hp_hsa-mir-199a-2_x_st	0.008	5.72E-01	0.957	5.42	5.41	5.42	0.070
hsa-miR-103-2-star_st	0.009	5.72E-01	0.957	5.41	5.40	5.41	0.076
hp_hsa-mir-920_st	0.009	5.72E-01	0.957	5.72	5.71	5.71	0.080
hsa-miR-1262_st	0.007	5.72E-01	0.957	5.35	5.34	5.35	0.061
hp_hsa-mir-3152_st	-0.009	5.72E-01	0.957	5.46	5.47	5.46	0.077
hsa-miR-223-star_st	-0.006	5.72E-01	0.957	5.33	5.33	5.33	0.052
hp_hsa-mir-27a_st	-0.023	5.73E-01	0.957	5.74	5.76	5.75	0.200
hp_hsa-mir-302d_st	-0.008	5.73E-01	0.957	5.39	5.40	5.39	0.072
hp_hsa-mir-194-1_st	-0.006	5.73E-01	0.957	5.32	5.33	5.32	0.048
hsa-miR-10a-star_st	0.021	5.74E-01	0.958	5.55	5.53	5.54	0.180
hsa-miR-18a-star_st	-0.053	5.74E-01	0.958	6.14	6.19	6.17	0.464
hp_hsa-mir-151_st	0.015	5.77E-01	0.958	6.10	6.09	6.09	0.136
hsa-miR-432-star_st	-0.008	5.77E-01	0.958	5.44	5.45	5.44	0.073
hp_hsa-mir-25_st	-0.013	5.78E-01	0.958	5.81	5.82	5.82	0.111
hp_hsa-mir-3165_st	0.006	5.78E-01	0.958	5.33	5.32	5.33	0.055
hp_hsa-mir-92a-2_x_st	-0.014	5.78E-01	0.958	6.57	6.58	6.57	0.127
hp_hsa-mir-573_st	-0.006	5.78E-01	0.958	5.38	5.39	5.39	0.054
hp_hsa-mir-1283-1_x_st	0.005	5.79E-01	0.958	5.30	5.29	5.29	0.047
hp_hsa-mir-208b_st	-0.006	5.80E-01	0.958	5.41	5.41	5.41	0.056
hp_hsa-mir-4257_st	-0.010	5.80E-01	0.958	5.72	5.74	5.73	0.093
hp_hsa-mir-320c-1_st	-0.035	5.81E-01	0.958	7.15	7.18	7.17	0.308
hp_hsa-mir-548h-3_st	-0.007	5.81E-01	0.958	5.45	5.46	5.46	0.060
hsa-miR-876-3p_st	0.005	5.81E-01	0.958	5.29	5.29	5.29	0.045
hsa-miR-548s_st	-0.005	5.81E-01	0.958	5.31	5.32	5.31	0.046
hp_hsa-mir-4295_st	0.006	5.81E-01	0.958	5.36	5.35	5.35	0.052
hsa-miR-126-star_st	-0.006	5.82E-01	0.958	5.31	5.32	5.32	0.054
hp_hsa-mir-606_x_st	-0.008	5.83E-01	0.958	5.42	5.43	5.42	0.068
hp_hsa-mir-19a_st	-0.006	5.83E-01	0.958	5.39	5.39	5.39	0.054
hp_hsa-mir-154_st	-0.006	5.83E-01	0.958	5.34	5.34	5.34	0.056
hsa-miR-548i_st	-0.006	5.84E-01	0.958	5.37	5.37	5.37	0.055
hp_hsa-mir-616_st	0.005	5.84E-01	0.958	5.35	5.35	5.35	0.047
hsa-miR-27a-star_st	0.053	5.84E-01	0.958	6.24	6.19	6.22	0.475
hsa-miR-3150_st	-0.008	5.85E-01	0.958	5.44	5.45	5.44	0.072
hsa-miR-541_st	0.008	5.86E-01	0.958	5.45	5.44	5.45	0.074
hp_hsa-mir-1913_st	-0.010	5.86E-01	0.958	5.60	5.61	5.61	0.090
hsa-miR-143_st	0.100	5.87E-01	0.958	12.01	11.91	11.97	0.906
hp_hsa-mir-3116-1_s_st	-0.006	5.87E-01	0.958	5.41	5.41	5.41	0.057

hp_hsa-mir-302c_st	-0.007	5.87E-01	0.958	5.42	5.43	5.42	0.061
hp_hsa-mir-629_st	-0.010	5.87E-01	0.958	6.03	6.04	6.04	0.092
hp_hsa-mir-133a-1_s_st	0.007	5.88E-01	0.958	5.38	5.37	5.37	0.061
hp_hsa-mir-224_st	0.029	5.88E-01	0.958	6.09	6.06	6.07	0.261
hp_hsa-mir-4274_st	-0.011	5.91E-01	0.958	5.63	5.64	5.64	0.097
hp_hsa-mir-323b_st	-0.006	5.91E-01	0.958	5.40	5.40	5.40	0.057
hp_hsa-mir-140_st	-0.010	5.91E-01	0.958	6.31	6.32	6.32	0.093
hsa-miR-517c_st	0.006	5.91E-01	0.958	5.28	5.27	5.28	0.055
hp_hsa-mir-647_st	-0.016	5.91E-01	0.958	6.40	6.41	6.40	0.148
hsa-miR-3154_st	0.027	5.91E-01	0.958	6.10	6.08	6.09	0.250
hp_hsa-mir-557_st	-0.009	5.91E-01	0.958	5.49	5.49	5.49	0.080
hsa-miR-624_st	-0.007	5.91E-01	0.958	5.30	5.31	5.31	0.061
hp_hsa-mir-3157_st	0.007	5.92E-01	0.958	5.46	5.45	5.45	0.060
hp_hsa-mir-635_st	-0.006	5.92E-01	0.958	5.36	5.37	5.36	0.054
hsa-miR-548e_st	-0.006	5.93E-01	0.958	5.28	5.29	5.29	0.053
hp_hsa-mir-3135_st	0.008	5.93E-01	0.958	5.38	5.37	5.38	0.069
hsa-miR-877_st	-0.035	5.93E-01	0.958	6.80	6.84	6.82	0.326
hsa-miR-4280_st	-0.005	5.96E-01	0.961	5.38	5.39	5.39	0.049
hsa-miR-34a-star_st	-0.015	5.97E-01	0.961	5.60	5.62	5.61	0.144
hsa-miR-873_st	0.005	5.98E-01	0.961	5.28	5.27	5.27	0.047
hsa-miR-548w_st	-0.006	5.98E-01	0.961	5.35	5.36	5.35	0.055
hsa-miR-1255a_st	-0.006	5.98E-01	0.961	5.36	5.36	5.36	0.060
hp_hsa-mir-1269_x_st	0.011	5.99E-01	0.961	5.69	5.67	5.68	0.103
hsa-miR-1185_st	0.007	5.99E-01	0.961	5.32	5.31	5.31	0.064
hp_hsa-mir-570_x_st	-0.007	5.99E-01	0.961	5.44	5.44	5.44	0.067
hp_hsa-mir-511-2_s_st	-0.006	5.99E-01	0.961	5.39	5.39	5.39	0.060
hp_hsa-mir-376a-1_x_st	0.006	6.00E-01	0.961	5.35	5.34	5.35	0.054
hp_hsa-mir-3155_st	0.009	6.00E-01	0.961	5.88	5.87	5.87	0.084
hsa-miR-2355_st	-0.007	6.01E-01	0.961	5.34	5.35	5.34	0.062
hsa-miR-450b-3p_st	-0.005	6.01E-01	0.961	5.27	5.27	5.27	0.051
hsa-miR-1229_st	-0.009	6.02E-01	0.961	5.54	5.55	5.55	0.089
hp_hsa-mir-548h-4_x_st	-0.006	6.02E-01	0.961	5.38	5.39	5.38	0.058
hp_hsa-mir-1282_st	-0.011	6.03E-01	0.961	5.77	5.79	5.78	0.105
hp_hsa-mir-3121_st	0.009	6.03E-01	0.961	5.61	5.60	5.60	0.080
hsa-miR-3141_st	-0.069	6.04E-01	0.961	9.03	9.10	9.06	0.653
hp_hsa-mir-3185_st	-0.035	6.04E-01	0.961	7.11	7.14	7.13	0.330
hsa-miR-155-star_st	-0.011	6.04E-01	0.961	5.44	5.45	5.45	0.105
hsa-miR-449c-star_st	-0.007	6.05E-01	0.961	5.34	5.34	5.34	0.064
hp_hsa-mir-329-1_s_st	-0.006	6.05E-01	0.961	5.39	5.40	5.39	0.052
hp_hsa-mir-9-1_st	0.007	6.05E-01	0.961	5.43	5.42	5.42	0.062
hsa-miR-34c-5p_st	0.009	6.06E-01	0.961	5.43	5.42	5.43	0.090
hp_hsa-mir-4279_st	-0.009	6.06E-01	0.961	5.46	5.47	5.47	0.084
hp_hsa-mir-4261_st	0.010	6.08E-01	0.963	5.82	5.81	5.82	0.091
hp_hsa-mir-200a_st	-0.013	6.08E-01	0.963	5.76	5.77	5.76	0.123
hsa-miR-374b-star_st	-0.006	6.09E-01	0.963	5.28	5.29	5.28	0.054
hsa-miR-518d-5p_st	0.008	6.09E-01	0.963	5.51	5.50	5.51	0.078
hp_hsa-mir-3186_st	0.009	6.09E-01	0.963	5.67	5.66	5.67	0.083

hsa-miR-34a_st	0.059	6.11E-01	0.963	9.03	8.97	9.00	0.570
hp_hsa-mir-548i-2_s_st	-0.007	6.11E-01	0.963	5.46	5.47	5.46	0.069
hp_hsa-mir-3115_st	0.005	6.11E-01	0.963	5.43	5.42	5.43	0.051
hp_hsa-mir-934_st	0.014	6.11E-01	0.963	6.07	6.05	6.06	0.131
hp_hsa-mir-520e_st	-0.006	6.12E-01	0.963	5.35	5.35	5.35	0.054
hp_hsa-mir-378b_x_st	-0.009	6.12E-01	0.963	5.64	5.65	5.64	0.085
hsa-miR-892b_st	-0.006	6.12E-01	0.963	5.34	5.34	5.34	0.054
hsa-miR-3168_st	-0.006	6.13E-01	0.963	5.32	5.32	5.32	0.057
hp_hsa-mir-649_st	-0.006	6.15E-01	0.965	5.37	5.38	5.38	0.055
hp_hsa-mir-3138_st	0.009	6.16E-01	0.965	5.69	5.68	5.69	0.087
hp_hsa-mir-4311_st	-0.009	6.17E-01	0.965	5.63	5.64	5.63	0.084
hsa-miR-586_st	-0.007	6.18E-01	0.965	5.43	5.43	5.43	0.066
hsa-miR-4315_st	-0.008	6.18E-01	0.965	5.31	5.32	5.32	0.078
hsa-miR-550-star_st	-0.018	6.18E-01	0.965	5.77	5.78	5.78	0.176
hp_hsa-mir-1244-2_s_st	-0.006	6.18E-01	0.965	5.36	5.36	5.36	0.055
hsa-miR-1247_st	-0.021	6.19E-01	0.965	5.59	5.61	5.60	0.207
hsa-miR-4275_st	0.007	6.19E-01	0.965	5.34	5.33	5.33	0.068
hp_hsa-mir-555_st	0.007	6.19E-01	0.965	5.42	5.42	5.42	0.066
hp_hsa-mir-181d_x_st	0.008	6.19E-01	0.965	5.51	5.50	5.51	0.076
hsa-miR-1287_st	-0.014	6.20E-01	0.965	5.65	5.66	5.65	0.139
hp_hsa-mir-642_st	-0.015	6.20E-01	0.965	6.14	6.15	6.15	0.152
hp_hsa-mir-3122_st	0.006	6.21E-01	0.966	5.37	5.36	5.37	0.061
hsa-miR-223_st	0.039	6.21E-01	0.966	6.07	6.03	6.05	0.392
hsa-miR-128_st	-0.037	6.21E-01	0.966	6.27	6.31	6.29	0.370
hsa-miR-593_st	0.006	6.23E-01	0.967	5.34	5.33	5.33	0.057
hp_hsa-mir-4267_st	0.019	6.24E-01	0.967	6.39	6.37	6.38	0.194
hp_hsa-mir-1193_st	-0.005	6.24E-01	0.967	5.43	5.44	5.44	0.054
hp_hsa-mir-516a-2_s_st	-0.006	6.24E-01	0.967	5.42	5.43	5.42	0.057
hp_hsa-mir-4302_st	-0.007	6.25E-01	0.967	5.50	5.51	5.51	0.070
hsa-miR-548f_st	-0.006	6.25E-01	0.968	5.33	5.34	5.33	0.060
hsa-miR-548o_st	0.005	6.26E-01	0.968	5.31	5.30	5.30	0.048
hp_hsa-mir-4303_st	-0.005	6.27E-01	0.968	5.31	5.32	5.31	0.045
hp_hsa-mir-338_s_st	0.005	6.27E-01	0.968	5.42	5.42	5.42	0.055
hsa-miR-2278_st	0.008	6.27E-01	0.968	5.57	5.56	5.57	0.078
hp_hsa-mir-562_st	0.005	6.28E-01	0.969	5.33	5.32	5.32	0.046
hsa-miR-519b-3p_st	-0.007	6.29E-01	0.969	5.39	5.39	5.39	0.069
hsa-miR-494_st	0.053	6.29E-01	0.969	9.73	9.68	9.71	0.537
hp_hsa-mir-582_x_st	0.005	6.30E-01	0.969	5.36	5.35	5.35	0.049
hsa-miR-197_st	0.030	6.32E-01	0.970	6.32	6.29	6.31	0.305
hsa-miR-1284_st	0.006	6.32E-01	0.970	5.35	5.35	5.35	0.057
hp_hsa-mir-199a-1_st	-0.005	6.33E-01	0.970	5.32	5.33	5.32	0.053
hp_hsa-mir-1302-3_s_st	-0.006	6.33E-01	0.970	5.37	5.38	5.38	0.059
hp_hsa-mir-3201_st	-0.005	6.33E-01	0.970	5.37	5.38	5.38	0.055
hsa-miR-3157_st	-0.006	6.33E-01	0.970	5.32	5.33	5.33	0.058
hsa-miR-105-star_st	0.006	6.34E-01	0.970	5.41	5.40	5.40	0.059
hp_hsa-mir-551b_st	-0.009	6.34E-01	0.970	5.66	5.67	5.66	0.089
hp_hsa-mir-449a_st	-0.006	6.34E-01	0.970	5.41	5.42	5.41	0.066

hp_hsa-mir-30e_st	-0.024	6.35E-01	0.970	6.01	6.03	6.02	0.254
hsa-miR-518c_st	-0.006	6.35E-01	0.970	5.27	5.27	5.27	0.060
hsa-miR-206_st	-0.009	6.35E-01	0.970	5.42	5.43	5.42	0.090
hp_hsa-mir-138-2_st	0.006	6.36E-01	0.970	5.43	5.43	5.43	0.063
hsa-miR-3179_st	0.005	6.37E-01	0.970	5.31	5.30	5.31	0.054
hp_hsa-mir-1827_st	-0.007	6.37E-01	0.970	5.49	5.50	5.50	0.069
hsa-miR-1267_st	0.006	6.38E-01	0.970	5.43	5.43	5.43	0.065
hp_hsa-mir-337_st	0.006	6.39E-01	0.970	5.46	5.46	5.46	0.067
hp_hsa-mir-1259_s_st	-0.018	6.40E-01	0.970	5.76	5.78	5.77	0.185
hsa-miR-505-star_st	-0.033	6.40E-01	0.970	6.47	6.50	6.49	0.345
hp_hsa-mir-30c-2_x_st	0.007	6.41E-01	0.970	5.60	5.60	5.60	0.077
hp_hsa-mir-548f-5_x_st	0.005	6.41E-01	0.970	5.46	5.45	5.46	0.057
hp_hsa-mir-422a_st	-0.005	6.42E-01	0.970	5.38	5.38	5.38	0.057
hsa-miR-520d-3p_st	0.007	6.42E-01	0.970	5.42	5.41	5.41	0.073
hp_hsa-mir-301b_x_st	-0.005	6.42E-01	0.970	5.34	5.35	5.34	0.050
hp_hsa-mir-1304_x_st	0.007	6.42E-01	0.970	5.39	5.39	5.39	0.068
hp_hsa-mir-1227_st	-0.018	6.44E-01	0.970	6.20	6.22	6.21	0.192
hsa-miR-1303_st	-0.009	6.44E-01	0.970	5.55	5.56	5.55	0.100
hp_hsa-mir-1274b_x_st	0.009	6.45E-01	0.970	5.58	5.57	5.57	0.096
hsa-miR-1207-3p_st	0.005	6.45E-01	0.970	5.33	5.33	5.33	0.054
hp_hsa-mir-26a-2_st	-0.006	6.46E-01	0.970	5.39	5.39	5.39	0.063
hsa-miR-27b-star_st	0.034	6.46E-01	0.970	6.17	6.13	6.15	0.369
hp_hsa-mir-1252_st	0.005	6.47E-01	0.970	5.45	5.44	5.45	0.051
hsa-miR-220a_st	0.005	6.47E-01	0.970	5.35	5.35	5.35	0.058
hp_hsa-mir-639_st	0.008	6.47E-01	0.970	5.58	5.57	5.57	0.089
hp_hsa-mir-4286_st	-0.006	6.47E-01	0.970	5.43	5.44	5.44	0.064
hp_hsa-mir-512-2_s_st	0.006	6.47E-01	0.970	5.53	5.52	5.52	0.069
hsa-miR-1226-star_st	0.026	6.47E-01	0.970	6.26	6.24	6.25	0.277
hsa-miR-765_st	0.015	6.47E-01	0.970	5.69	5.68	5.68	0.165
hp_hsa-mir-548k_st	-0.005	6.48E-01	0.970	5.33	5.34	5.33	0.053
hsa-miR-936_st	-0.019	6.48E-01	0.970	6.03	6.05	6.04	0.207
hp_hsa-mir-612_st	-0.012	6.49E-01	0.970	5.89	5.90	5.89	0.133
hp_hsa-mir-4317_st	0.006	6.49E-01	0.970	5.45	5.44	5.44	0.066
hsa-miR-577_st	0.005	6.50E-01	0.970	5.30	5.30	5.30	0.052
hp_hsa-mir-644_st	-0.004	6.50E-01	0.970	5.31	5.32	5.31	0.046
hsa-miR-3116_st	0.006	6.50E-01	0.970	5.35	5.35	5.35	0.064
hsa-miR-549_st	0.005	6.51E-01	0.971	5.37	5.36	5.36	0.052
hsa-miR-483-3p_st	-0.009	6.51E-01	0.971	5.56	5.57	5.57	0.100
hsa-miR-185-star_st	-0.006	6.53E-01	0.971	5.49	5.50	5.49	0.066
hsa-miR-1305_st	-0.005	6.53E-01	0.971	5.41	5.41	5.41	0.056
hsa-miR-618_st	0.005	6.53E-01	0.971	5.31	5.30	5.30	0.049
hsa-miR-99a-star_st	0.010	6.54E-01	0.971	5.55	5.54	5.54	0.110
hp_hsa-mir-671_st	0.006	6.55E-01	0.971	5.52	5.52	5.52	0.063
hp_hsa-mir-720_st	-0.006	6.56E-01	0.971	5.47	5.48	5.47	0.067
hsa-miR-24_st	0.033	6.56E-01	0.971	13.21	13.18	13.20	0.364
hsa-miR-671-5p_st	-0.029	6.57E-01	0.971	7.17	7.20	7.19	0.318
hp_hsa-mir-548a-3_x_st	-0.007	6.58E-01	0.971	5.45	5.46	5.45	0.081

hp_hsa-mir-3167_x_st	0.006	6.59E-01	0.971	5.48	5.48	5.48	0.061
hp_hsa-mir-4305_st	-0.005	6.59E-01	0.971	5.39	5.40	5.40	0.053
hsa-miR-222-star_st	0.007	6.59E-01	0.971	5.50	5.50	5.50	0.073
hp_hsa-mir-326_st	-0.026	6.60E-01	0.971	6.43	6.46	6.45	0.291
hsa-miR-516a-5p_st	0.005	6.60E-01	0.971	5.33	5.32	5.32	0.052
hp_hsa-mir-527_x_st	-0.005	6.60E-01	0.971	5.38	5.38	5.38	0.053
hsa-miR-4314_st	0.012	6.61E-01	0.971	5.63	5.62	5.63	0.133
hsa-miR-452_st	0.024	6.61E-01	0.971	5.72	5.70	5.71	0.266
hsa-miR-542-3p_st	-0.008	6.61E-01	0.971	5.52	5.53	5.53	0.086
hsa-miR-323b-5p_st	0.005	6.61E-01	0.971	5.36	5.36	5.36	0.060
hp_hsa-mir-361_st	0.013	6.61E-01	0.971	6.47	6.45	6.46	0.147
hp_hsa-mir-103-2_s_st	0.007	6.62E-01	0.971	5.46	5.46	5.46	0.076
hp_hsa-mir-1246_st	-0.012	6.63E-01	0.971	5.82	5.84	5.83	0.131
hp_hsa-mir-221_st	0.007	6.64E-01	0.971	5.56	5.55	5.55	0.085
hsa-miR-3153_st	-0.013	6.64E-01	0.971	5.73	5.74	5.74	0.145
hp_hsa-mir-3190_s_st	-0.008	6.64E-01	0.971	5.52	5.52	5.52	0.086
hsa-miR-513a-5p_st	-0.021	6.64E-01	0.971	6.10	6.12	6.11	0.241
hp_hsa-mir-610_x_st	-0.005	6.66E-01	0.971	5.41	5.42	5.42	0.054
hp_hsa-mir-301b_st	0.003	6.67E-01	0.971	5.31	5.31	5.31	0.039
hp_hsa-mir-30c-1_st	-0.006	6.68E-01	0.971	5.49	5.50	5.49	0.068
hsa-miR-3174_st	-0.006	6.69E-01	0.971	5.49	5.49	5.49	0.068
hsa-miR-150_st	0.083	6.69E-01	0.971	8.82	8.74	8.78	0.957
hp_hsa-mir-16-2_x_st	0.010	6.69E-01	0.971	6.09	6.08	6.08	0.111
hp_hsa-mir-1255a_x_st	-0.004	6.71E-01	0.971	5.33	5.33	5.33	0.046
hsa-miR-19b_st	0.065	6.71E-01	0.971	8.11	8.05	8.08	0.758
hp_hsa-mir-663b_st	-0.019	6.71E-01	0.971	6.15	6.17	6.16	0.218
hp_hsa-mir-374b_x_st	0.005	6.71E-01	0.971	5.34	5.34	5.34	0.062
hp_hsa-mir-606_st	-0.005	6.71E-01	0.971	5.40	5.40	5.40	0.061
hp_hsa-mir-4330_st	0.008	6.72E-01	0.971	5.53	5.52	5.53	0.088
hsa-miR-29b-1-star_st	-0.037	6.72E-01	0.971	6.16	6.19	6.18	0.428
hp_hsa-mir-324_st	-0.007	6.72E-01	0.971	5.85	5.86	5.85	0.084
hsa-miR-182-star_st	0.005	6.73E-01	0.971	5.36	5.35	5.35	0.060
hp_hsa-mir-1201_s_st	-0.020	6.73E-01	0.971	6.03	6.05	6.04	0.231
hp_hsa-mir-942_st	-0.005	6.74E-01	0.971	5.35	5.35	5.35	0.052
hsa-miR-517-star_st	0.005	6.75E-01	0.971	5.32	5.32	5.32	0.054
hp_hsa-mir-4253_x_st	-0.006	6.75E-01	0.971	5.55	5.56	5.56	0.076
hsa-miR-641_st	-0.006	6.75E-01	0.971	5.39	5.39	5.39	0.067
hsa-miR-135b-star_st	0.013	6.78E-01	0.971	5.62	5.61	5.61	0.150
hp_hsa-mir-609_x_st	0.006	6.78E-01	0.971	5.43	5.42	5.42	0.069
hp_hsa-mir-643_st	-0.006	6.79E-01	0.971	5.51	5.52	5.51	0.070
hp_hsa-mir-581_st	0.005	6.79E-01	0.971	5.41	5.41	5.41	0.063
hsa-miR-26a-2-star_st	0.005	6.80E-01	0.971	5.38	5.38	5.38	0.063
hsa-miR-216b_st	-0.008	6.80E-01	0.971	5.58	5.59	5.58	0.094
hp_hsa-mir-4328_st	0.005	6.80E-01	0.971	5.37	5.37	5.37	0.061
hsa-miR-563_st	0.009	6.81E-01	0.971	5.55	5.54	5.54	0.111
hsa-miR-4309_st	0.005	6.81E-01	0.971	5.33	5.32	5.33	0.055
hp_hsa-mir-508_st	-0.004	6.81E-01	0.971	5.34	5.35	5.34	0.051

hsa-miR-141-star_st	0.004	6.81E-01	0.971	5.29	5.28	5.29	0.046
hsa-miR-613_st	-0.005	6.82E-01	0.971	5.33	5.34	5.34	0.059
hp_hsa-mir-761_x_st	-0.005	6.83E-01	0.971	5.38	5.39	5.39	0.060
hp_hsa-mir-525_x_st	-0.016	6.83E-01	0.971	6.33	6.35	6.34	0.190
hsa-miR-511_st	0.004	6.83E-01	0.971	5.25	5.25	5.25	0.052
hp_hsa-mir-505_st	0.007	6.83E-01	0.971	5.50	5.49	5.50	0.083
hsa-miR-150-star_st	-0.033	6.84E-01	0.971	7.28	7.32	7.30	0.402
hp_hsa-mir-548q_st	-0.012	6.84E-01	0.971	5.71	5.72	5.71	0.141
hp_hsa-mir-549_st	-0.005	6.84E-01	0.971	5.37	5.38	5.38	0.054
hp_hsa-mir-1181_st	-0.018	6.85E-01	0.971	6.80	6.82	6.81	0.219
hp_hsa-mir-548l_st	-0.005	6.85E-01	0.971	5.38	5.39	5.38	0.060
hsa-miR-3129_st	-0.004	6.86E-01	0.971	5.32	5.32	5.32	0.051
hsa-miR-3189_st	0.007	6.86E-01	0.971	5.46	5.45	5.46	0.084
hsa-miR-1255b_st	0.006	6.86E-01	0.971	5.41	5.40	5.41	0.067
hsa-miR-660_st	-0.031	6.87E-01	0.971	6.59	6.62	6.61	0.372
hsa-miR-148a-star_st	-0.012	6.87E-01	0.971	5.50	5.51	5.51	0.148
hp_hsa-mir-941-2_s_st	-0.014	6.88E-01	0.971	6.22	6.24	6.23	0.170
hsa-miR-555_st	-0.005	6.88E-01	0.971	5.31	5.32	5.32	0.055
hp_hsa-mir-1973_st	-0.013	6.88E-01	0.971	6.05	6.06	6.06	0.157
hp_hsa-mir-631_x_st	-0.006	6.88E-01	0.971	5.51	5.52	5.51	0.079
hsa-miR-3164_st	0.009	6.89E-01	0.971	5.53	5.52	5.53	0.107
hp_hsa-mir-593_st	-0.009	6.90E-01	0.971	5.64	5.65	5.64	0.106
hp_hsa-mir-103-1_s_st	-0.005	6.90E-01	0.971	5.32	5.33	5.33	0.056
hsa-miR-4298_st	0.035	6.91E-01	0.971	8.29	8.26	8.27	0.427
hsa-miR-4262_st	-0.009	6.91E-01	0.971	5.29	5.30	5.29	0.110
hsa-miR-302d_st	0.004	6.91E-01	0.971	5.30	5.29	5.29	0.054
hp_hsa-mir-525_st	-0.014	6.92E-01	0.971	6.47	6.48	6.47	0.175
hp_hsa-mir-1224_st	-0.023	6.92E-01	0.971	7.16	7.18	7.17	0.284
hsa-miR-215_st	0.052	6.92E-01	0.971	6.15	6.10	6.12	0.649
hsa-miR-367_st	0.004	6.93E-01	0.971	5.30	5.29	5.29	0.053
hsa-miR-1281_st	0.042	6.93E-01	0.971	8.10	8.06	8.08	0.528
hp_hsa-mir-153-2_st	-0.004	6.94E-01	0.971	5.31	5.31	5.31	0.049
hsa-miR-3161_st	0.005	6.94E-01	0.971	5.39	5.38	5.39	0.060
hsa-miR-1207-5p_st	-0.049	6.94E-01	0.971	9.82	9.87	9.84	0.609
hp_hsa-mir-504_st	0.008	6.96E-01	0.971	5.74	5.73	5.73	0.100
hp_hsa-mir-922_st	0.007	6.96E-01	0.971	5.55	5.54	5.54	0.086
hp_hsa-mir-125b-1_st	0.006	6.97E-01	0.971	5.52	5.51	5.51	0.074
hsa-miR-146b-3p_st	-0.010	6.97E-01	0.971	5.47	5.48	5.48	0.130
hsa-miR-136_st	0.004	6.98E-01	0.971	5.31	5.31	5.31	0.053
hsa-miR-2053_st	-0.004	6.98E-01	0.971	5.34	5.34	5.34	0.046
hsa-miR-377_st	-0.005	6.98E-01	0.971	5.36	5.36	5.36	0.057
hp_hsa-mir-183_st	-0.010	6.98E-01	0.971	5.64	5.65	5.64	0.127
hsa-miR-3136_st	0.005	6.98E-01	0.971	5.50	5.49	5.49	0.068
hp_hsa-mir-450a-2_st	-0.004	6.99E-01	0.971	5.35	5.35	5.35	0.045
hsa-miR-1208_st	0.012	6.99E-01	0.971	5.55	5.54	5.54	0.156
hsa-miR-488_st	-0.008	6.99E-01	0.971	5.42	5.43	5.42	0.098
hsa-miR-135a-star_st	-0.016	6.99E-01	0.971	6.14	6.16	6.15	0.206

hsa-miR-1825_st	0.020	7.00E-01	0.971	6.44	6.43	6.44	0.255
hp_hsa-mir-3158-2_s_st	0.010	7.02E-01	0.971	5.68	5.67	5.67	0.123
hp_hsa-mir-146b_x_st	0.006	7.02E-01	0.971	5.53	5.52	5.52	0.077
hsa-miR-362-3p_st	0.004	7.03E-01	0.971	5.29	5.29	5.29	0.047
hsa-miR-3158_st	0.005	7.03E-01	0.971	5.31	5.31	5.31	0.061
hsa-miR-625-star_st	0.005	7.03E-01	0.971	5.43	5.42	5.42	0.070
hsa-miR-1225-5p_st	-0.045	7.04E-01	0.971	7.94	7.99	7.97	0.579
hp_hsa-mir-1302-1_st	0.004	7.04E-01	0.971	5.32	5.32	5.32	0.053
hp_hsa-mir-654_st	-0.006	7.04E-01	0.971	5.48	5.48	5.48	0.075
hp_hsa-mir-100_st	-0.004	7.05E-01	0.971	5.34	5.35	5.34	0.051
hsa-miR-140-5p_st	0.018	7.05E-01	0.971	5.75	5.73	5.74	0.228
hsa-miR-3169_st	-0.004	7.05E-01	0.971	5.39	5.40	5.40	0.054
hsa-miR-4273_st	-0.005	7.05E-01	0.971	5.36	5.37	5.37	0.070
hsa-miR-2115_st	-0.004	7.06E-01	0.971	5.28	5.28	5.28	0.057
hp_hsa-mir-3123_st	-0.010	7.07E-01	0.971	5.74	5.75	5.75	0.126
hsa-miR-93-star_st	-0.025	7.07E-01	0.971	6.23	6.26	6.25	0.324
hsa-miR-553_st	-0.004	7.08E-01	0.971	5.31	5.31	5.31	0.058
hsa-miR-645_st	-0.004	7.08E-01	0.971	5.27	5.28	5.28	0.049
hp_hsa-mir-548f-3_x_st	0.004	7.08E-01	0.971	5.39	5.39	5.39	0.058
hp_hsa-mir-92a-2_st	-0.006	7.08E-01	0.971	5.47	5.48	5.47	0.076
hsa-miR-636_st	0.009	7.08E-01	0.971	5.56	5.55	5.55	0.113
hsa-miR-938_st	0.005	7.09E-01	0.971	5.34	5.34	5.34	0.067
hp_hsa-mir-369_st	-0.005	7.09E-01	0.971	5.39	5.40	5.40	0.061
hp_hsa-mir-185_x_st	-0.007	7.09E-01	0.971	6.04	6.04	6.04	0.095
hsa-miR-3170_st	0.005	7.09E-01	0.971	5.33	5.33	5.33	0.061
hsa-miR-885-3p_st	-0.057	7.09E-01	0.971	6.80	6.85	6.82	0.752
hp_hsa-mir-18b_x_st	0.008	7.09E-01	0.971	5.57	5.56	5.57	0.099
hp_hsa-mir-582_st	0.004	7.10E-01	0.971	5.39	5.39	5.39	0.052
hsa-miR-1252_st	0.004	7.10E-01	0.971	5.32	5.31	5.32	0.050
hp_hsa-mir-494_st	0.007	7.11E-01	0.971	5.83	5.82	5.82	0.096
hsa-miR-218_st	0.004	7.11E-01	0.971	5.29	5.28	5.29	0.050
hsa-miR-1270_st	-0.006	7.11E-01	0.971	5.46	5.46	5.46	0.076
hsa-miR-296-5p_st	0.006	7.12E-01	0.971	5.45	5.45	5.45	0.075
hp_hsa-mir-137_st	-0.004	7.13E-01	0.971	5.41	5.42	5.41	0.054
hp_hsa-mir-1537_st	0.004	7.13E-01	0.971	5.34	5.33	5.33	0.049
hsa-miR-591_st	-0.004	7.14E-01	0.971	5.26	5.26	5.26	0.050
hp_hsa-mir-4253_st	-0.006	7.14E-01	0.971	5.60	5.60	5.60	0.076
hsa-miR-526b_st	0.004	7.14E-01	0.971	5.32	5.31	5.32	0.054
hp_hsa-mir-298_st	0.006	7.14E-01	0.971	5.44	5.44	5.44	0.074
hp_hsa-mir-548u_x_st	-0.007	7.14E-01	0.971	5.50	5.51	5.51	0.093
hsa-miR-933_st	-0.019	7.15E-01	0.971	7.25	7.26	7.25	0.262
hsa-miR-934_st	0.007	7.15E-01	0.971	5.53	5.52	5.52	0.098
hsa-miR-202_st	-0.015	7.16E-01	0.971	5.79	5.80	5.79	0.201
hp_hsa-mir-548f-1_st	-0.009	7.17E-01	0.971	5.53	5.54	5.54	0.115
hsa-let-7g-star_st	0.006	7.18E-01	0.971	5.45	5.45	5.45	0.081
hp_hsa-mir-548t_st	0.004	7.18E-01	0.971	5.32	5.32	5.32	0.048
hp_hsa-mir-1296_st	0.007	7.18E-01	0.971	5.70	5.69	5.69	0.089

hsa-miR-1231_st	-0.041	7.19E-01	0.971	6.92	6.97	6.94	0.555
hsa-miR-509-5p_st	-0.007	7.19E-01	0.971	5.46	5.47	5.47	0.096
hsa-miR-1248_st	-0.004	7.19E-01	0.971	5.37	5.38	5.37	0.058
hp_hsa-mir-199b_st	0.008	7.19E-01	0.971	5.71	5.70	5.71	0.110
hp_hsa-mir-1180_st	-0.007	7.20E-01	0.971	5.75	5.76	5.75	0.090
hp_hsa-mir-220b_st	-0.004	7.21E-01	0.971	5.37	5.37	5.37	0.057
hp_hsa-mir-515-1_s_st	-0.004	7.22E-01	0.971	5.39	5.39	5.39	0.056
hsa-miR-639_st	0.018	7.23E-01	0.971	5.73	5.72	5.73	0.244
hsa-miR-505_st	-0.014	7.23E-01	0.971	5.60	5.61	5.60	0.189
hsa-miR-509-3-5p_st	0.009	7.23E-01	0.971	5.57	5.56	5.56	0.130
hsa-miR-655_st	0.005	7.23E-01	0.971	5.31	5.30	5.30	0.065
hp_hsa-mir-3165_x_st	-0.004	7.24E-01	0.971	5.37	5.38	5.37	0.049
hsa-miR-1246_st	-0.061	7.24E-01	0.971	8.83	8.89	8.86	0.846
hsa-miR-1178_st	0.004	7.25E-01	0.971	5.29	5.29	5.29	0.052
hsa-miR-3125_st	0.005	7.26E-01	0.971	5.39	5.38	5.39	0.074
hsa-miR-450a_st	-0.004	7.26E-01	0.971	5.31	5.32	5.32	0.056
hsa-miR-516b-star_st	-0.004	7.26E-01	0.971	5.33	5.33	5.33	0.058
hp_hsa-mir-1238_st	-0.013	7.27E-01	0.971	6.00	6.01	6.01	0.178
hsa-miR-4260_st	-0.010	7.27E-01	0.971	5.81	5.82	5.81	0.139
hsa-miR-4293_st	-0.006	7.27E-01	0.971	5.45	5.46	5.45	0.086
hsa-miR-20b-star_st	0.006	7.28E-01	0.971	5.57	5.56	5.56	0.088
hp_hsa-mir-658_st	0.015	7.28E-01	0.971	6.17	6.16	6.16	0.209
hsa-miR-4324_st	0.006	7.28E-01	0.971	5.43	5.43	5.43	0.090
hsa-miR-1182_st	0.014	7.28E-01	0.971	5.86	5.85	5.85	0.200
hsa-miR-522_st	-0.007	7.31E-01	0.971	5.43	5.44	5.43	0.097
hp_hsa-mir-634_st	0.005	7.31E-01	0.971	5.54	5.53	5.54	0.068
hp_hsa-mir-450a-1_st	-0.004	7.31E-01	0.971	5.39	5.40	5.40	0.061
hsa-miR-4299_st	-0.039	7.32E-01	0.971	7.43	7.47	7.45	0.554
hsa-miR-3123_st	-0.004	7.32E-01	0.971	5.26	5.26	5.26	0.055
hp_hsa-mir-1278_x_st	-0.005	7.32E-01	0.971	5.39	5.40	5.39	0.071
hp_hsa-mir-3172_st	-0.010	7.33E-01	0.971	6.08	6.09	6.08	0.148
hsa-miR-3190-3p_st	-0.010	7.34E-01	0.971	5.60	5.61	5.60	0.145
hp_hsa-mir-3175_st	-0.007	7.34E-01	0.971	5.73	5.74	5.73	0.101
hp_hsa-mir-1302-2_s_st	-0.004	7.34E-01	0.971	5.37	5.38	5.37	0.052
hsa-miR-3193_st	-0.004	7.35E-01	0.971	5.36	5.36	5.36	0.065
hp_hsa-mir-4262_st	0.008	7.36E-01	0.971	5.58	5.58	5.58	0.114
hsa-miR-557_st	0.013	7.36E-01	0.971	5.81	5.80	5.80	0.190
hp_hsa-mir-513a-2_s_st	0.003	7.36E-01	0.971	5.31	5.30	5.30	0.048
hsa-miR-145_st	0.053	7.36E-01	0.971	13.32	13.27	13.29	0.767
hsa-miR-25_st	0.040	7.36E-01	0.971	8.64	8.60	8.62	0.591
hsa-miR-302b-star_st	0.004	7.37E-01	0.971	5.36	5.35	5.35	0.051
hsa-miR-939_st	-0.039	7.37E-01	0.971	7.65	7.69	7.67	0.567
hsa-miR-331-5p_st	0.012	7.37E-01	0.971	5.83	5.82	5.83	0.178
hp_hsa-mir-492_st	0.005	7.38E-01	0.971	5.42	5.41	5.41	0.070
hsa-miR-342-5p_st	0.031	7.38E-01	0.971	6.47	6.44	6.46	0.457
hsa-miR-611_st	-0.004	7.38E-01	0.971	5.40	5.40	5.40	0.064
hp_hsa-mir-1285-2_x_st	-0.006	7.39E-01	0.971	5.75	5.75	5.75	0.095

hp_hsa-mir-3183_st	0.005	7.39E-01	0.971	5.49	5.48	5.49	0.079
hp_hsa-mir-1825_st	-0.009	7.39E-01	0.971	5.72	5.72	5.72	0.128
hp_hsa-mir-29b-2_x_st	-0.005	7.39E-01	0.971	5.40	5.41	5.41	0.067
hsa-miR-1269_st	-0.017	7.39E-01	0.971	5.40	5.41	5.41	0.248
hp_hsa-mir-640_st	-0.006	7.40E-01	0.971	5.52	5.53	5.53	0.090
hp_hsa-mir-941-3_s_st	-0.011	7.40E-01	0.971	6.23	6.24	6.24	0.161
hp_hsa-mir-199a-2_st	-0.005	7.41E-01	0.971	5.47	5.47	5.47	0.071
hp_hsa-mir-211_st	-0.004	7.42E-01	0.972	5.51	5.51	5.51	0.062
hp_hsa-mir-3158-1_s_st	0.006	7.44E-01	0.973	5.46	5.46	5.46	0.085
hp_hsa-mir-1286_st	0.004	7.44E-01	0.973	5.35	5.34	5.35	0.054
hp_hsa-mir-611_st	-0.008	7.46E-01	0.973	5.79	5.80	5.79	0.120
hp_hsa-mir-216b_st	-0.004	7.46E-01	0.973	5.39	5.39	5.39	0.054
hp_hsa-mir-181b-1_x_st	-0.005	7.46E-01	0.973	5.43	5.44	5.44	0.070
hp_hsa-mir-548b_x_st	0.003	7.47E-01	0.973	5.31	5.30	5.31	0.045
hsa-miR-3180-5p_st	0.005	7.47E-01	0.973	5.47	5.46	5.47	0.076
hsa-miR-3159_st	0.004	7.48E-01	0.973	5.34	5.34	5.34	0.055
hp_hsa-mir-1274b_st	-0.006	7.49E-01	0.973	5.54	5.54	5.54	0.088
hp_hsa-mir-133a-2_st	0.004	7.49E-01	0.973	5.40	5.40	5.40	0.059
hsa-miR-589-star_st	-0.006	7.50E-01	0.973	5.51	5.51	5.51	0.089
hp_hsa-mir-3166_x_st	0.004	7.50E-01	0.973	5.35	5.34	5.34	0.058
hsa-miR-3190-5p_st	0.007	7.50E-01	0.973	5.50	5.50	5.50	0.110
hp_hsa-mir-4327_st	-0.005	7.51E-01	0.973	5.47	5.47	5.47	0.072
hp_hsa-mir-3149_st	0.004	7.51E-01	0.973	5.35	5.35	5.35	0.068
hsa-miR-4297_st	0.005	7.52E-01	0.973	5.41	5.41	5.41	0.072
hp_hsa-mir-1298_st	-0.005	7.52E-01	0.973	5.47	5.48	5.47	0.085
hsa-miR-4265_st	-0.004	7.53E-01	0.973	5.34	5.35	5.35	0.069
hp_hsa-mir-943_st	-0.010	7.53E-01	0.973	6.05	6.06	6.06	0.158
hsa-miR-19b-2-star_st	-0.003	7.53E-01	0.973	5.27	5.27	5.27	0.050
hsa-miR-575_st	0.026	7.53E-01	0.973	6.45	6.42	6.44	0.404
hp_hsa-mir-3150_x_st	0.006	7.54E-01	0.973	5.65	5.65	5.65	0.088
hsa-miR-331-3p_st	0.023	7.54E-01	0.973	6.43	6.41	6.42	0.355
hp_hsa-mir-204_st	-0.006	7.54E-01	0.973	5.77	5.78	5.77	0.094
hsa-miR-1234_st	0.009	7.54E-01	0.973	5.80	5.80	5.80	0.139
hp_hsa-mir-1289-2_x_st	-0.006	7.54E-01	0.973	5.55	5.55	5.55	0.087
hsa-miR-889_st	0.003	7.54E-01	0.973	5.31	5.31	5.31	0.052
hp_hsa-mir-1208_st	-0.009	7.55E-01	0.973	5.88	5.88	5.88	0.145
hsa-miR-1911_st	-0.008	7.55E-01	0.973	5.39	5.39	5.39	0.121
hp_hsa-mir-7-3_st	0.004	7.55E-01	0.973	5.53	5.53	5.53	0.063
hp_hsa-mir-4310_st	0.004	7.56E-01	0.973	5.51	5.51	5.51	0.068
hp_hsa-mir-196a-1_st	0.004	7.57E-01	0.973	5.43	5.43	5.43	0.068
hsa-miR-1914_st	0.004	7.58E-01	0.973	5.39	5.38	5.38	0.066
hsa-miR-3134_st	-0.004	7.58E-01	0.973	5.43	5.44	5.44	0.070
hsa-miR-617_st	0.005	7.59E-01	0.973	5.42	5.41	5.41	0.081
hsa-miR-205-star_st	0.004	7.60E-01	0.973	5.42	5.42	5.42	0.069
hp_hsa-mir-129-2_x_st	0.005	7.60E-01	0.973	5.44	5.44	5.44	0.072
hp_hsa-mir-3178_st	-0.007	7.60E-01	0.973	6.01	6.02	6.02	0.114
hp_hsa-mir-1245_st	-0.004	7.61E-01	0.973	5.28	5.29	5.28	0.060

hsa-miR-510_st	-0.009	7.61E-01	0.973	5.65	5.66	5.65	0.139
hsa-miR-520b_st	-0.004	7.62E-01	0.973	5.35	5.36	5.35	0.058
hp_hsa-mir-548s_x_st	-0.003	7.62E-01	0.973	5.31	5.31	5.31	0.047
hsa-miR-4256_st	-0.003	7.62E-01	0.973	5.31	5.31	5.31	0.049
hp_hsa-mir-450a-1_x_st	-0.004	7.63E-01	0.973	5.45	5.45	5.45	0.066
hp_hsa-mir-548h-1_st	-0.003	7.63E-01	0.973	5.38	5.39	5.39	0.053
hp_hsa-mir-4312_st	-0.006	7.63E-01	0.973	5.55	5.55	5.55	0.094
hp_hsa-mir-548g_st	-0.007	7.63E-01	0.973	5.47	5.47	5.47	0.110
hsa-miR-19b-1-star_st	-0.004	7.65E-01	0.973	5.34	5.34	5.34	0.073
hsa-miR-877-star_st	-0.006	7.66E-01	0.973	5.52	5.53	5.53	0.103
hsa-miR-499-3p_st	0.004	7.66E-01	0.973	5.34	5.34	5.34	0.062
hp_hsa-mir-3173_st	-0.004	7.67E-01	0.973	5.49	5.49	5.49	0.074
hp_hsa-mir-1197_st	0.005	7.68E-01	0.973	5.42	5.41	5.41	0.090
hp_hsa-mir-517b_x_st	0.003	7.69E-01	0.973	5.34	5.34	5.34	0.051
hsa-miR-301a_st	0.005	7.69E-01	0.973	5.36	5.35	5.35	0.084
hp_hsa-mir-590_st	-0.003	7.70E-01	0.973	5.34	5.34	5.34	0.050
hsa-miR-890_st	-0.008	7.70E-01	0.973	5.52	5.53	5.53	0.137
hsa-miR-4281_st	-0.038	7.70E-01	0.973	11.13	11.16	11.15	0.645
hsa-miR-495_st	-0.005	7.70E-01	0.973	5.39	5.39	5.39	0.081
hsa-miR-188-3p_st	-0.004	7.71E-01	0.973	5.36	5.36	5.36	0.058
hp_hsa-mir-33b_st	-0.009	7.71E-01	0.973	5.99	6.00	6.00	0.148
hp_hsa-mir-220b_s_st	-0.005	7.71E-01	0.973	5.59	5.60	5.59	0.084
hp_hsa-mir-637_st	-0.005	7.71E-01	0.973	5.58	5.58	5.58	0.084
hp_hsa-mir-320e_x_st	-0.011	7.72E-01	0.973	6.26	6.27	6.26	0.182
hp_hsa-mir-153-2_x_st	-0.003	7.72E-01	0.973	5.36	5.36	5.36	0.056
hsa-miR-520c-5p_st	-0.004	7.72E-01	0.973	5.41	5.42	5.42	0.073
hsa-miR-1279_st	0.004	7.73E-01	0.973	5.33	5.33	5.33	0.059
hp_hsa-mir-769_st	-0.005	7.73E-01	0.973	5.67	5.67	5.67	0.088
hp_hsa-mir-873_st	0.004	7.73E-01	0.973	5.42	5.41	5.41	0.067
hp_hsa-mir-1185-1_s_st	0.007	7.73E-01	0.973	6.15	6.15	6.15	0.116
hsa-miR-1537_st	0.003	7.74E-01	0.973	5.32	5.31	5.32	0.049
hp_hsa-mir-18b_st	0.003	7.74E-01	0.973	5.39	5.38	5.38	0.055
hsa-miR-3155_st	-0.004	7.74E-01	0.973	5.47	5.48	5.48	0.077
hsa-miR-1468_st	-0.007	7.75E-01	0.973	5.51	5.52	5.51	0.125
hsa-miR-3156_st	-0.017	7.76E-01	0.973	6.68	6.70	6.69	0.299
hsa-miR-7-1-star_st	-0.006	7.76E-01	0.973	5.48	5.48	5.48	0.098
hsa-miR-1280_st	-0.033	7.76E-01	0.973	9.57	9.61	9.59	0.581
hp_hsa-mir-4287_st	0.007	7.76E-01	0.973	6.26	6.25	6.26	0.128
hp_hsa-mir-32_st	-0.005	7.77E-01	0.973	5.47	5.47	5.47	0.083
hsa-miR-19a_st	-0.008	7.78E-01	0.973	5.54	5.55	5.55	0.136
hsa-miR-532-3p_st	-0.024	7.78E-01	0.973	6.84	6.87	6.86	0.415
hp_hsa-mir-4289_st	-0.004	7.79E-01	0.973	5.53	5.53	5.53	0.074
hsa-miR-383_st	-0.005	7.80E-01	0.973	5.45	5.46	5.45	0.092
hp_hsa-mir-561_x_st	-0.003	7.80E-01	0.973	5.42	5.42	5.42	0.056
hp_hsa-mir-523_st	0.003	7.80E-01	0.973	5.36	5.36	5.36	0.058
hsa-miR-135b_st	0.003	7.80E-01	0.973	5.30	5.29	5.29	0.055
hp_hsa-mir-548b_st	-0.003	7.82E-01	0.973	5.35	5.35	5.35	0.048

hp_hsa-mir-4270_st	-0.011	7.82E-01	0.973	6.32	6.33	6.33	0.194
hsa-miR-15b-star_st	-0.003	7.82E-01	0.973	5.29	5.29	5.29	0.053
hsa-miR-1973_st	0.024	7.82E-01	0.973	7.21	7.19	7.20	0.427
hsa-miR-764_st	0.005	7.82E-01	0.973	5.40	5.40	5.40	0.081
hp_hsa-mir-4309_st	-0.003	7.82E-01	0.973	5.41	5.41	5.41	0.059
hp_hsa-mir-544b_st	-0.004	7.84E-01	0.973	5.31	5.31	5.31	0.065
hp_hsa-mir-628_st	-0.005	7.84E-01	0.973	5.63	5.63	5.63	0.085
hsa-miR-216a_st	-0.005	7.84E-01	0.973	5.47	5.47	5.47	0.087
hp_hsa-mir-3119-1_s_st	0.003	7.84E-01	0.973	5.36	5.36	5.36	0.054
hp_hsa-mir-548v_st	-0.003	7.86E-01	0.974	5.32	5.33	5.33	0.047
hp_hsa-mir-365-1_x_st	0.005	7.86E-01	0.974	5.54	5.54	5.54	0.089
hsa-miR-524-5p_st	0.004	7.86E-01	0.974	5.41	5.40	5.41	0.078
hp_hsa-mir-148b_st	-0.003	7.87E-01	0.974	5.40	5.40	5.40	0.054
hp_hsa-mir-365-1_st	0.005	7.88E-01	0.974	5.59	5.58	5.58	0.100
hsa-miR-204_st	-0.007	7.88E-01	0.974	5.37	5.37	5.37	0.128
hsa-miR-151-5p_st	0.024	7.88E-01	0.974	10.80	10.78	10.79	0.436
hp_hsa-mir-340_st	0.003	7.90E-01	0.974	5.34	5.34	5.34	0.057
hsa-miR-384_st	0.003	7.90E-01	0.974	5.32	5.32	5.32	0.058
hsa-miR-335-star_st	-0.004	7.91E-01	0.974	5.35	5.35	5.35	0.065
hp_hsa-mir-3189_st	0.004	7.91E-01	0.974	5.51	5.51	5.51	0.066
hsa-miR-4286_st	-0.042	7.91E-01	0.974	8.74	8.79	8.76	0.780
hp_hsa-mir-365-2_st	-0.004	7.92E-01	0.974	5.49	5.49	5.49	0.067
hp_hsa-mir-548n_x_st	-0.004	7.92E-01	0.974	5.49	5.50	5.50	0.080
hp_hsa-mir-659_st	0.003	7.92E-01	0.974	5.37	5.37	5.37	0.051
hsa-miR-205_st	-0.011	7.92E-01	0.974	5.38	5.39	5.39	0.196
hp_hsa-mir-1261_st	0.004	7.94E-01	0.975	5.46	5.45	5.46	0.066
hsa-miR-1302_st	-0.003	7.96E-01	0.975	5.32	5.32	5.32	0.055
hp_hsa-mir-548i-3_x_st	0.003	7.96E-01	0.975	5.35	5.34	5.35	0.049
hp_hsa-mir-587_st	-0.003	7.97E-01	0.975	5.53	5.53	5.53	0.059
hsa-miR-4289_st	-0.003	7.97E-01	0.975	5.38	5.39	5.39	0.065
hp_hsa-mir-1250_st	-0.003	7.98E-01	0.975	5.45	5.46	5.46	0.061
hp_hsa-mir-511-1_s_st	-0.003	7.98E-01	0.975	5.37	5.38	5.37	0.053
hp_hsa-mir-95_st	-0.004	7.98E-01	0.975	5.45	5.46	5.45	0.074
hsa-miR-606_st	-0.004	7.99E-01	0.975	5.44	5.44	5.44	0.078
hsa-miR-664_st	0.005	8.00E-01	0.975	5.56	5.55	5.56	0.093
hsa-miR-484_st	0.008	8.00E-01	0.975	5.73	5.72	5.73	0.147
hsa-miR-548b-5p_st	0.003	8.02E-01	0.975	5.31	5.31	5.31	0.057
hsa-miR-106b-star_st	0.024	8.02E-01	0.975	7.45	7.42	7.44	0.464
hp_hsa-mir-596_st	-0.006	8.02E-01	0.975	5.60	5.61	5.60	0.113
hsa-miR-486-3p_st	0.011	8.03E-01	0.975	5.64	5.63	5.63	0.217
hsa-miR-363-star_st	0.004	8.03E-01	0.975	5.41	5.40	5.41	0.075
hsa-miR-129-5p_st	-0.015	8.04E-01	0.975	5.82	5.84	5.83	0.301
hsa-miR-27a_st	0.030	8.04E-01	0.975	10.48	10.45	10.46	0.597
hsa-miR-2110_st	0.012	8.04E-01	0.975	6.49	6.48	6.48	0.238
hp_hsa-mir-133a-1_x_st	-0.003	8.05E-01	0.975	5.36	5.36	5.36	0.055
hsa-miR-4317_st	0.006	8.05E-01	0.975	5.95	5.94	5.94	0.119
hp_hsa-mir-603_st	-0.003	8.05E-01	0.975	5.39	5.39	5.39	0.063

hsa-miR-615-5p_st	0.006	8.05E-01	0.975	5.54	5.54	5.54	0.125
hsa-miR-454_st	0.006	8.06E-01	0.975	5.43	5.42	5.42	0.111
hsa-miR-523_st	0.003	8.07E-01	0.975	5.34	5.34	5.34	0.052
hp_hsa-mir-3170_st	-0.002	8.08E-01	0.975	5.37	5.37	5.37	0.046
hsa-miR-1237_st	0.007	8.08E-01	0.975	5.78	5.77	5.78	0.150
hsa-miR-566_st	0.003	8.08E-01	0.975	5.41	5.40	5.40	0.062
hsa-miR-2113_st	-0.003	8.09E-01	0.975	5.39	5.40	5.40	0.064
hp_hsa-mir-1183_st	0.003	8.10E-01	0.975	5.42	5.42	5.42	0.056
hsa-miR-339-5p_st	0.024	8.10E-01	0.975	6.98	6.95	6.97	0.490
hp_hsa-mir-128-1_x_st	-0.004	8.10E-01	0.975	5.65	5.65	5.65	0.091
hsa-miR-656_st	0.003	8.10E-01	0.975	5.33	5.33	5.33	0.059
hp_hsa-mir-576_st	-0.003	8.10E-01	0.975	5.49	5.49	5.49	0.069
hsa-miR-200a_st	-0.039	8.10E-01	0.975	8.33	8.37	8.35	0.799
hsa-miR-548n_st	-0.002	8.10E-01	0.975	5.28	5.28	5.28	0.046
hp_hsa-mir-193a_st	0.006	8.11E-01	0.975	6.35	6.35	6.35	0.129
hp_hsa-mir-559_x_st	0.004	8.12E-01	0.975	5.49	5.48	5.48	0.084
hp_hsa-mir-519e_st	0.003	8.12E-01	0.975	5.41	5.40	5.41	0.067
hp_hsa-mir-1229_st	-0.013	8.12E-01	0.975	6.63	6.64	6.63	0.272
hsa-miR-1296_st	0.008	8.12E-01	0.975	5.87	5.86	5.86	0.159
hp_hsa-mir-1206_st	0.002	8.12E-01	0.975	5.32	5.32	5.32	0.046
hp_hsa-mir-1185-2_s_st	0.006	8.12E-01	0.975	6.08	6.07	6.07	0.130
hsa-miR-21-star_st	-0.018	8.13E-01	0.975	6.99	7.01	7.00	0.366
hp_hsa-mir-184_st	-0.003	8.13E-01	0.975	5.39	5.39	5.39	0.068
hp_hsa-mir-4321_st	-0.006	8.13E-01	0.975	6.03	6.03	6.03	0.133
hsa-miR-483-5p_st	-0.045	8.13E-01	0.975	6.77	6.81	6.79	0.939
hp_hsa-mir-891a_st	-0.003	8.14E-01	0.975	5.40	5.40	5.40	0.069
hp_hsa-mir-519d_x_st	0.003	8.15E-01	0.975	5.47	5.47	5.47	0.066
hp_hsa-mir-519a-2_x_st	0.002	8.15E-01	0.975	5.33	5.33	5.33	0.049
hsa-miR-17-star_st	-0.025	8.15E-01	0.975	6.67	6.69	6.68	0.523
hp_hsa-mir-150_st	-0.006	8.17E-01	0.975	5.90	5.90	5.90	0.122
hp_hsa-mir-4283-2_s_st	-0.003	8.17E-01	0.975	5.36	5.36	5.36	0.054
hsa-miR-761_st	0.003	8.17E-01	0.975	5.38	5.37	5.37	0.060
hsa-miR-632_st	0.003	8.19E-01	0.975	5.37	5.37	5.37	0.055
hp_hsa-mir-138-1_x_st	0.004	8.19E-01	0.975	5.76	5.76	5.76	0.092
hsa-miR-659_st	-0.007	8.19E-01	0.975	5.79	5.80	5.80	0.156
hsa-miR-196a-star_st	-0.003	8.19E-01	0.975	5.29	5.30	5.29	0.058
hp_hsa-mir-483_st	-0.009	8.20E-01	0.975	5.78	5.79	5.78	0.191
hp_hsa-mir-1205_st	-0.002	8.21E-01	0.975	5.33	5.33	5.33	0.052
hp_hsa-mir-9-2_x_st	0.003	8.21E-01	0.975	5.34	5.34	5.34	0.056
hsa-miR-4272_st	-0.002	8.21E-01	0.975	5.30	5.30	5.30	0.052
hp_hsa-mir-3156-2_x_st	0.004	8.21E-01	0.975	5.51	5.51	5.51	0.078
hp_hsa-mir-1256_st	0.002	8.21E-01	0.975	5.32	5.32	5.32	0.049
hp_hsa-mir-548m_st	0.003	8.22E-01	0.975	5.36	5.36	5.36	0.058
hp_hsa-mir-513a-1_s_st	0.003	8.22E-01	0.975	5.42	5.41	5.42	0.057
hp_hsa-mir-655_st	0.002	8.22E-01	0.975	5.34	5.34	5.34	0.048
hp_hsa-mir-452_st	0.004	8.22E-01	0.975	5.46	5.45	5.45	0.086
hsa-miR-4270_st	0.034	8.23E-01	0.975	11.05	11.01	11.03	0.738

hp_hsa-mir-3199-1_s_st	-0.003	8.23E-01	0.975	5.39	5.39	5.39	0.056
hp_hsa-mir-200b_st	-0.010	8.24E-01	0.975	7.04	7.05	7.05	0.217
hp_hsa-mir-1258_s_st	0.004	8.25E-01	0.975	5.48	5.47	5.47	0.080
hsa-miR-449b_st	0.003	8.26E-01	0.976	5.38	5.38	5.38	0.066
hsa-miR-1915-star_st	-0.006	8.26E-01	0.976	5.53	5.54	5.54	0.124
hsa-miR-4257_st	0.009	8.27E-01	0.976	5.93	5.92	5.93	0.207
hp_hsa-mir-519d_st	0.002	8.28E-01	0.976	5.40	5.40	5.40	0.054
hsa-miR-342-3p_st	0.028	8.28E-01	0.976	9.68	9.65	9.67	0.636
hp_hsa-mir-518c_st	0.003	8.29E-01	0.976	5.48	5.48	5.48	0.063
hp_hsa-let-7f-1_x_st	0.003	8.29E-01	0.976	5.61	5.61	5.61	0.079
hp_hsa-mir-552_st	0.006	8.30E-01	0.977	5.50	5.49	5.50	0.137
hsa-miR-34b_st	-0.004	8.30E-01	0.977	5.53	5.54	5.53	0.083
hsa-miR-323b-3p_st	0.004	8.31E-01	0.978	5.57	5.56	5.56	0.092
hp_hsa-mir-3160-2_s_st	-0.003	8.32E-01	0.978	5.39	5.40	5.39	0.057
hsa-miR-3151_st	-0.007	8.33E-01	0.979	5.83	5.84	5.83	0.152
hp_hsa-mir-3179-2_s_st	0.003	8.34E-01	0.979	5.45	5.44	5.44	0.062
hp_hsa-mir-3184_s_st	-0.004	8.35E-01	0.980	5.57	5.57	5.57	0.083
hsa-miR-892a_st	0.002	8.35E-01	0.980	5.29	5.29	5.29	0.057
hp_hsa-mir-30c-2_st	0.003	8.36E-01	0.980	5.55	5.55	5.55	0.068
hsa-miR-1259_st	-0.002	8.37E-01	0.980	5.38	5.38	5.38	0.057
hp_hsa-mir-147b_st	0.003	8.37E-01	0.980	5.34	5.34	5.34	0.061
hp_hsa-mir-1279_st	0.002	8.39E-01	0.980	5.34	5.33	5.33	0.046
hp_hsa-mir-220c_st	0.002	8.39E-01	0.980	5.45	5.45	5.45	0.058
hsa-miR-551b-star_st	0.011	8.39E-01	0.980	6.52	6.51	6.51	0.258
hp_hsa-mir-495_x_st	0.002	8.40E-01	0.980	5.30	5.30	5.30	0.049
hp_hsa-mir-924_st	0.002	8.40E-01	0.980	5.35	5.35	5.35	0.049
hp_hsa-mir-107_st	-0.002	8.41E-01	0.980	5.38	5.38	5.38	0.059
hsa-miR-1263_st	-0.009	8.42E-01	0.980	5.90	5.91	5.91	0.233
hp_hsa-mir-410_st	0.002	8.42E-01	0.980	5.32	5.32	5.32	0.049
hp_hsa-mir-3202-1_s_st	-0.002	8.43E-01	0.980	5.31	5.32	5.32	0.051
hp_hsa-mir-21_st	0.008	8.43E-01	0.980	6.15	6.15	6.15	0.197
hp_hsa-mir-34b_st	-0.002	8.44E-01	0.980	5.36	5.36	5.36	0.058
hp_hsa-mir-133a-1_st	-0.002	8.45E-01	0.980	5.30	5.30	5.30	0.053
hp_hsa-mir-466_st	-0.003	8.45E-01	0.980	5.41	5.41	5.41	0.073
hsa-miR-208b_st	0.002	8.45E-01	0.980	5.35	5.35	5.35	0.056
hp_hsa-mir-1272_st	0.002	8.46E-01	0.980	5.45	5.45	5.45	0.057
hp_hsa-mir-3135_x_st	0.003	8.46E-01	0.980	5.54	5.53	5.54	0.076
hsa-miR-1914-star_st	-0.007	8.46E-01	0.980	5.93	5.94	5.94	0.182
hsa-miR-16-2-star_st	-0.006	8.46E-01	0.980	5.61	5.62	5.61	0.154
hp_hsa-mir-548a-2_x_st	0.004	8.47E-01	0.980	5.57	5.57	5.57	0.093
hsa-miR-492_st	-0.009	8.47E-01	0.980	5.61	5.62	5.62	0.228
hp_hsa-mir-921_st	0.003	8.47E-01	0.980	5.43	5.43	5.43	0.066
hp_hsa-mir-297_st	-0.002	8.47E-01	0.980	5.35	5.35	5.35	0.053
hp_hsa-mir-181d_st	0.003	8.48E-01	0.980	5.60	5.59	5.60	0.079
hp_hsa-mir-1253_x_st	-0.002	8.49E-01	0.980	5.37	5.37	5.37	0.061
hp_hsa-mir-320c-1_x_st	-0.010	8.49E-01	0.980	6.81	6.82	6.81	0.246
hp_hsa-mir-891b_st	0.003	8.49E-01	0.980	5.41	5.41	5.41	0.065

hp_hsa-mir-526a-2_st	-0.002	8.49E-01	0.980	5.37	5.38	5.37	0.054
hp_hsa-mir-759_st	-0.003	8.49E-01	0.980	5.40	5.41	5.40	0.064
hsa-miR-610_st	0.002	8.50E-01	0.980	5.36	5.36	5.36	0.059
hsa-miR-3152_st	0.006	8.50E-01	0.980	5.66	5.66	5.66	0.166
hp_hsa-mir-554_st	0.003	8.51E-01	0.981	5.42	5.42	5.42	0.066
hp_hsa-mir-135a-1_st	-0.003	8.53E-01	0.981	5.46	5.46	5.46	0.076
hp_hsa-mir-29b-1_st	-0.003	8.53E-01	0.981	5.39	5.39	5.39	0.073
hp_hsa-mir-3156-3_x_st	0.004	8.53E-01	0.981	5.50	5.49	5.50	0.099
hsa-miR-519a-star_st	0.003	8.54E-01	0.981	5.36	5.36	5.36	0.072
hp_hsa-mir-219-1_st	0.002	8.55E-01	0.981	5.44	5.43	5.43	0.066
hsa-miR-141_st	-0.024	8.55E-01	0.981	7.17	7.19	7.18	0.644
hp_hsa-mir-550-1_s_st	0.003	8.55E-01	0.981	5.88	5.87	5.88	0.092
hp_hsa-mir-4268_st	0.002	8.56E-01	0.981	5.38	5.38	5.38	0.055
hsa-miR-221_st	-0.022	8.56E-01	0.981	11.18	11.20	11.19	0.595
hsa-miR-373-star_st	-0.007	8.56E-01	0.981	5.65	5.65	5.65	0.187
hsa-miR-766_st	-0.005	8.58E-01	0.982	5.97	5.98	5.97	0.143
hp_hsa-mir-4277_st	0.003	8.58E-01	0.982	5.46	5.45	5.46	0.071
hp_hsa-mir-4260_st	0.003	8.58E-01	0.982	5.57	5.57	5.57	0.094
hsa-miR-3195_st	0.012	8.59E-01	0.982	7.52	7.51	7.52	0.337
hsa-miR-624-star_st	0.002	8.59E-01	0.982	5.38	5.37	5.38	0.065
hsa-miR-33b-star_st	-0.003	8.59E-01	0.982	5.64	5.65	5.65	0.083
hp_hsa-mir-124-2_x_st	0.002	8.60E-01	0.982	5.55	5.55	5.55	0.069
hsa-miR-4282_st	0.002	8.61E-01	0.982	5.30	5.30	5.30	0.066
hp_hsa-mir-626_st	0.002	8.62E-01	0.982	5.34	5.34	5.34	0.054
hsa-miR-552_st	-0.022	8.62E-01	0.982	6.04	6.07	6.05	0.619
hsa-miR-3197_st	-0.014	8.62E-01	0.982	8.33	8.35	8.34	0.396
hsa-miR-33a_st	0.002	8.63E-01	0.982	5.27	5.27	5.27	0.049
hp_hsa-mir-375_st	-0.011	8.63E-01	0.982	6.33	6.34	6.34	0.322
hsa-miR-579_st	0.002	8.63E-01	0.982	5.38	5.37	5.38	0.060
hsa-miR-600_st	0.002	8.64E-01	0.982	5.38	5.38	5.38	0.061
hsa-miR-1236_st	-0.002	8.64E-01	0.982	5.37	5.37	5.37	0.069
hsa-miR-548v_st	-0.002	8.65E-01	0.982	5.30	5.30	5.30	0.052
hsa-miR-299-3p_st	0.004	8.66E-01	0.982	5.64	5.64	5.64	0.104
hsa-miR-548j_st	0.002	8.67E-01	0.982	5.34	5.33	5.34	0.047
hsa-miR-631_st	-0.002	8.67E-01	0.982	5.42	5.42	5.42	0.064
hsa-miR-374b_st	0.004	8.67E-01	0.982	5.45	5.45	5.45	0.112
hp_hsa-mir-99a_st	-0.003	8.68E-01	0.982	5.46	5.46	5.46	0.080
hsa-miR-921_st	-0.004	8.69E-01	0.982	5.66	5.66	5.66	0.133
hp_hsa-mir-2115_st	-0.002	8.69E-01	0.982	5.37	5.37	5.37	0.056
hsa-miR-452-star_st	0.002	8.69E-01	0.982	5.32	5.32	5.32	0.057
hsa-miR-3139_st	-0.002	8.69E-01	0.982	5.39	5.40	5.40	0.055
hsa-miR-711_st	-0.005	8.69E-01	0.982	5.74	5.74	5.74	0.156
hsa-miR-616-star_st	-0.002	8.69E-01	0.982	5.40	5.40	5.40	0.060
hp_hsa-mir-586_st	0.002	8.70E-01	0.982	5.42	5.42	5.42	0.058
hp_hsa-mir-548c_x_st	0.003	8.70E-01	0.982	5.42	5.42	5.42	0.101
hsa-miR-374a_st	-0.002	8.70E-01	0.982	5.38	5.38	5.38	0.058
hp_hsa-mir-941-4_s_st	0.006	8.71E-01	0.982	6.33	6.32	6.33	0.191

hsa-miR-1260b_st	-0.021	8.71E-01	0.982	8.86	8.88	8.87	0.649
hp_hsa-mir-34c_st	-0.002	8.72E-01	0.982	5.49	5.49	5.49	0.061
hp_hsa-mir-1299_st	0.002	8.72E-01	0.982	5.45	5.45	5.45	0.056
hsa-miR-1273c_st	-0.003	8.74E-01	0.982	5.60	5.60	5.60	0.095
hp_hsa-mir-323b_x_st	-0.002	8.74E-01	0.982	5.49	5.50	5.50	0.057
hp_hsa-mir-1910_st	-0.005	8.74E-01	0.982	6.07	6.07	6.07	0.169
hp_hsa-mir-30d_st	0.004	8.74E-01	0.982	6.04	6.04	6.04	0.108
hsa-miR-193a-3p_st	0.009	8.74E-01	0.982	5.98	5.97	5.98	0.289
hp_hsa-mir-610_st	-0.002	8.75E-01	0.982	5.36	5.37	5.36	0.055
hsa-miR-1285_st	-0.005	8.76E-01	0.982	6.02	6.02	6.02	0.153
hsa-miR-3121_st	0.003	8.77E-01	0.982	5.61	5.61	5.61	0.101
hp_hsa-mir-615_st	0.003	8.77E-01	0.982	5.67	5.67	5.67	0.089
hsa-miR-136-star_st	-0.002	8.78E-01	0.982	5.30	5.30	5.30	0.062
hp_hsa-mir-625_st	0.002	8.78E-01	0.982	5.44	5.44	5.44	0.060
hp_hsa-mir-519a-1_x_st	-0.002	8.78E-01	0.982	5.36	5.36	5.36	0.053
hsa-miR-589_st	0.002	8.78E-01	0.982	5.33	5.33	5.33	0.055
hp_hsa-mir-181a-2_st	0.002	8.79E-01	0.982	5.45	5.45	5.45	0.069
hp_hsa-mir-1284_st	0.002	8.80E-01	0.982	5.46	5.45	5.45	0.056
hp_hsa-let-7a-1_st	0.002	8.81E-01	0.982	5.57	5.57	5.57	0.078
hsa-miR-324-3p_st	0.009	8.81E-01	0.982	7.03	7.02	7.03	0.302
hsa-miR-361-5p_st	0.014	8.81E-01	0.982	10.04	10.03	10.03	0.474
hsa-miR-3137_st	-0.007	8.81E-01	0.982	5.80	5.81	5.81	0.219
hp_hsa-mir-10b_st	-0.002	8.81E-01	0.982	5.38	5.39	5.38	0.056
hsa-miR-548d-5p_st	-0.002	8.82E-01	0.982	5.45	5.45	5.45	0.065
hsa-miR-640_st	-0.003	8.82E-01	0.982	5.63	5.63	5.63	0.099
hsa-miR-520d-5p_st	-0.002	8.83E-01	0.982	5.41	5.41	5.41	0.071
hp_hsa-mir-1277_st	0.002	8.83E-01	0.982	5.34	5.34	5.34	0.051
hp_hsa-mir-1258_x_st	-0.002	8.84E-01	0.982	5.39	5.39	5.39	0.055
hsa-miR-3135_st	0.002	8.84E-01	0.982	5.44	5.44	5.44	0.068
hsa-miR-302a-star_st	-0.002	8.84E-01	0.982	5.33	5.34	5.34	0.055
hsa-miR-1275_st	-0.014	8.86E-01	0.983	8.16	8.17	8.16	0.482
hp_hsa-mir-519e_x_st	0.002	8.86E-01	0.983	5.37	5.37	5.37	0.056
hp_hsa-let-7a-1_x_st	0.002	8.86E-01	0.983	5.49	5.49	5.49	0.069
hp_hsa-mir-101-1_x_st	-0.002	8.86E-01	0.983	5.31	5.31	5.31	0.054
hsa-miR-137_st	0.001	8.87E-01	0.983	5.33	5.33	5.33	0.050
hsa-miR-450b-5p_st	0.004	8.88E-01	0.983	5.63	5.63	5.63	0.144
hp_hsa-mir-613_st	0.001	8.89E-01	0.983	5.34	5.34	5.34	0.043
hp_hsa-mir-325_st	0.002	8.89E-01	0.983	5.29	5.29	5.29	0.056
hp_hsa-mir-941-1_s_st	-0.004	8.89E-01	0.983	6.09	6.09	6.09	0.147
hp_hsa-mir-196a-2_st	0.002	8.90E-01	0.983	5.33	5.33	5.33	0.066
hsa-miR-657_st	0.002	8.90E-01	0.983	5.33	5.32	5.33	0.056
hsa-miR-200c-star_st	-0.004	8.91E-01	0.983	5.51	5.51	5.51	0.153
hp_hsa-mir-2110_st	0.003	8.91E-01	0.983	5.67	5.67	5.67	0.090
hsa-miR-1323_st	-0.006	8.92E-01	0.983	5.85	5.85	5.85	0.205
hsa-miR-2277_st	0.008	8.92E-01	0.983	6.20	6.20	6.20	0.278
hp_hsa-mir-548j_st	-0.002	8.93E-01	0.983	5.53	5.53	5.53	0.086
hp_hsa-mir-521-2_x_st	-0.002	8.93E-01	0.983	5.39	5.40	5.39	0.060

hp_hsa-mir-1287_st	0.002	8.94E-01	0.983	5.37	5.37	5.37	0.057
hsa-miR-4278_st	-0.002	8.94E-01	0.983	5.35	5.35	5.35	0.069
hsa-let-7b-star_st	0.004	8.94E-01	0.983	5.77	5.76	5.77	0.160
hsa-miR-4271_st	0.011	8.94E-01	0.983	6.33	6.32	6.33	0.390
hsa-miR-587_st	0.002	8.95E-01	0.983	5.50	5.49	5.50	0.068
hsa-miR-183_st	-0.013	8.95E-01	0.983	6.01	6.02	6.02	0.502
hp_hsa-mir-503_st	0.004	8.98E-01	0.985	5.75	5.75	5.75	0.145
hp_hsa-mir-541_st	0.002	8.98E-01	0.985	5.52	5.52	5.52	0.066
hsa-miR-1911-star_st	0.002	8.99E-01	0.986	5.47	5.47	5.47	0.093
hsa-miR-1245_st	0.002	8.99E-01	0.986	5.43	5.43	5.43	0.072
hp_hsa-mir-3137_st	-0.002	8.99E-01	0.986	5.48	5.48	5.48	0.078
hp_hsa-mir-1293_st	-0.002	9.01E-01	0.987	5.54	5.54	5.54	0.059
hsa-miR-526b-star_st	-0.001	9.01E-01	0.987	5.29	5.29	5.29	0.053
hp_hsa-mir-411_st	-0.001	9.03E-01	0.987	5.40	5.41	5.40	0.056
hp_hsa-mir-1322_st	0.001	9.03E-01	0.987	5.33	5.33	5.33	0.050
hsa-miR-302c-star_st	-0.002	9.03E-01	0.987	5.53	5.53	5.53	0.070
hsa-miR-1238_st	0.005	9.06E-01	0.988	5.96	5.96	5.96	0.222
hp_hsa-mir-1-2_st	0.001	9.06E-01	0.988	5.46	5.46	5.46	0.051
hsa-miR-1224-5p_st	0.012	9.06E-01	0.988	7.14	7.12	7.13	0.496
hp_hsa-mir-135a-2_st	0.001	9.07E-01	0.988	5.35	5.35	5.35	0.052
hsa-miR-3163_st	0.004	9.07E-01	0.988	5.78	5.78	5.78	0.170
hp_hsa-mir-1255b-2_st	0.001	9.07E-01	0.988	5.33	5.33	5.33	0.048
hsa-miR-665_st	-0.006	9.07E-01	0.988	6.58	6.59	6.58	0.271
hsa-miR-548a-3p_st	0.006	9.09E-01	0.988	6.05	6.05	6.05	0.277
hsa-miR-1201_st	0.007	9.09E-01	0.988	5.74	5.73	5.74	0.288
hsa-miR-218-2-star_st	-0.001	9.09E-01	0.988	5.37	5.37	5.37	0.052
hsa-miR-92a_st	0.015	9.10E-01	0.988	12.11	12.10	12.10	0.657
hsa-miR-582-5p_st	-0.001	9.10E-01	0.988	5.30	5.30	5.30	0.056
hsa-miR-1227_st	-0.002	9.10E-01	0.988	5.45	5.46	5.45	0.066
hp_hsa-mir-151_x_st	0.004	9.12E-01	0.989	6.63	6.62	6.62	0.168
hp_hsa-mir-129-1_x_st	-0.002	9.12E-01	0.989	5.40	5.40	5.40	0.069
hp_hsa-mir-135b_st	0.001	9.13E-01	0.989	5.36	5.36	5.36	0.059
hp_hsa-mir-4252_st	0.001	9.13E-01	0.989	5.42	5.42	5.42	0.056
hp_hsa-mir-1297_st	0.001	9.13E-01	0.989	5.43	5.43	5.43	0.061
hp_hsa-mir-646_st	-0.001	9.14E-01	0.989	5.40	5.40	5.40	0.056
hp_hsa-mir-328_st	-0.003	9.14E-01	0.989	5.95	5.95	5.95	0.129
hsa-miR-4303_st	-0.001	9.15E-01	0.989	5.41	5.41	5.41	0.066
hsa-miR-1277_st	0.001	9.15E-01	0.989	5.35	5.35	5.35	0.050
hp_hsa-mir-517c_x_st	0.001	9.15E-01	0.989	5.35	5.35	5.35	0.046
hp_hsa-mir-548i-3_s_st	0.002	9.16E-01	0.989	5.48	5.48	5.48	0.073
hp_hsa-mir-515-2_s_st	0.001	9.16E-01	0.989	5.37	5.37	5.37	0.053
hp_hsa-mir-2113_st	-0.001	9.16E-01	0.989	5.39	5.39	5.39	0.064
hp_hsa-mir-300_st	-0.001	9.18E-01	0.989	5.39	5.39	5.39	0.060
hsa-miR-1181_st	-0.002	9.18E-01	0.989	5.52	5.52	5.52	0.096
hp_hsa-mir-1260b_st	0.002	9.18E-01	0.989	6.12	6.12	6.12	0.103
hsa-miR-554_st	-0.003	9.18E-01	0.989	5.44	5.44	5.44	0.135
hsa-miR-523-star_st	-0.001	9.19E-01	0.989	5.27	5.27	5.27	0.051

hp_hsa-mir-660_st	-0.002	9.19E-01	0.989	5.81	5.81	5.81	0.093
hp_hsa-let-7f-1_st	0.001	9.21E-01	0.991	5.47	5.46	5.46	0.057
hp_hsa-mir-548h-2_x_st	-0.002	9.22E-01	0.992	5.56	5.56	5.56	0.103
hp_hsa-mir-101-1_st	0.001	9.24E-01	0.992	5.34	5.34	5.34	0.055
hp_hsa-mir-4314_st	0.002	9.24E-01	0.992	5.59	5.58	5.59	0.085
hp_hsa-mir-4326_st	-0.002	9.25E-01	0.993	5.93	5.93	5.93	0.125
hp_hsa-mir-668_st	0.002	9.26E-01	0.993	5.66	5.66	5.66	0.103
hsa-miR-1471_st	-0.002	9.26E-01	0.993	5.70	5.70	5.70	0.119
hp_hsa-let-7g_st	0.002	9.27E-01	0.993	5.74	5.74	5.74	0.084
hp_hsa-mir-580_x_st	0.001	9.27E-01	0.993	5.38	5.38	5.38	0.054
hsa-miR-491-3p_st	-0.001	9.27E-01	0.993	5.33	5.33	5.33	0.054
hsa-miR-548b-3p_st	-0.001	9.27E-01	0.993	5.31	5.31	5.31	0.053
hp_hsa-mir-518b_x_st	-0.001	9.29E-01	0.993	5.43	5.43	5.43	0.063
hp_hsa-mir-101-2_x_st	-0.001	9.29E-01	0.993	5.34	5.34	5.34	0.054
hsa-miR-3186-5p_st	-0.001	9.29E-01	0.993	5.43	5.44	5.43	0.073
hsa-miR-222_st	-0.011	9.30E-01	0.993	11.05	11.06	11.05	0.597
hp_hsa-mir-4307_st	0.001	9.30E-01	0.993	5.32	5.32	5.32	0.057
hp_hsa-mir-548i-2_x_st	-0.001	9.31E-01	0.994	5.34	5.34	5.34	0.057
hp_hsa-mir-376a-2_st	0.001	9.32E-01	0.994	5.34	5.34	5.34	0.054
hsa-miR-559_st	-0.001	9.33E-01	0.994	5.36	5.36	5.36	0.064
hp_hsa-mir-885_st	-0.005	9.33E-01	0.994	6.43	6.43	6.43	0.286
hsa-miR-517a_st	0.001	9.33E-01	0.994	5.30	5.30	5.30	0.057
hsa-miR-3142_st	-0.001	9.34E-01	0.994	5.29	5.30	5.29	0.060
hp_hsa-mir-302c_x_st	0.001	9.34E-01	0.994	5.36	5.36	5.36	0.056
hp_hsa-mir-548i-2_st	-0.001	9.34E-01	0.994	5.40	5.41	5.40	0.071
hsa-miR-3144-5p_st	-0.001	9.35E-01	0.994	5.33	5.33	5.33	0.061
hsa-miR-670_st	0.001	9.36E-01	0.994	5.33	5.33	5.33	0.070
hsa-miR-4291_st	0.001	9.36E-01	0.994	5.42	5.42	5.42	0.069
hsa-miR-367-star_st	-0.001	9.37E-01	0.994	5.29	5.29	5.29	0.050
hp_hsa-mir-4319_st	0.001	9.38E-01	0.994	5.37	5.37	5.37	0.049
hsa-miR-595_st	0.001	9.38E-01	0.994	5.62	5.62	5.62	0.081
hsa-miR-513c_st	0.001	9.38E-01	0.994	5.44	5.43	5.44	0.072
hp_hsa-mir-487a_x_st	-0.001	9.40E-01	0.995	5.47	5.47	5.47	0.067
hp_hsa-mir-3169_st	0.001	9.41E-01	0.995	5.36	5.36	5.36	0.063
hsa-miR-545-star_st	0.001	9.41E-01	0.995	5.30	5.30	5.30	0.050
hp_hsa-mir-4308_st	0.001	9.42E-01	0.995	5.33	5.33	5.33	0.049
hp_hsa-mir-659_x_st	0.001	9.42E-01	0.995	5.40	5.40	5.40	0.063
hsa-miR-875-5p_st	-0.001	9.43E-01	0.995	5.26	5.26	5.26	0.056
hp_hsa-mir-190_x_st	0.001	9.43E-01	0.995	5.39	5.39	5.39	0.075
hp_hsa-mir-205_st	-0.001	9.44E-01	0.995	5.45	5.45	5.45	0.057
hsa-miR-19a-star_st	-0.001	9.45E-01	0.995	5.35	5.35	5.35	0.054
hp_hsa-mir-24-2_st	0.001	9.45E-01	0.995	5.45	5.45	5.45	0.085
hsa-miR-147_st	0.001	9.45E-01	0.995	5.32	5.32	5.32	0.054
hp_hsa-mir-1262_st	-0.001	9.46E-01	0.995	5.50	5.50	5.50	0.063
hp_hsa-mir-493_st	-0.001	9.46E-01	0.995	5.46	5.46	5.46	0.061
hp_hsa-mir-892b_x_st	0.001	9.46E-01	0.995	5.38	5.38	5.38	0.053
hsa-miR-103-as_st	-0.001	9.47E-01	0.995	5.46	5.46	5.46	0.084

hsa-miR-633_st	-0.001	9.47E-01	0.995	5.31	5.31	5.31	0.055
hp_hsa-mir-498_st	-0.001	9.48E-01	0.995	5.41	5.41	5.41	0.059
hsa-miR-501-5p_st	-0.003	9.48E-01	0.995	6.00	6.01	6.00	0.251
hsa-miR-3172_st	-0.009	9.48E-01	0.995	8.07	8.08	8.07	0.649
hp_hsa-mir-24-1_s_st	-0.001	9.49E-01	0.995	5.33	5.33	5.33	0.057
hsa-miR-219-2-3p_st	0.001	9.49E-01	0.995	5.42	5.42	5.42	0.051
hsa-miR-767-3p_st	0.001	9.49E-01	0.995	5.36	5.36	5.36	0.061
hp_hsa-mir-301a_x_st	-0.001	9.50E-01	0.995	5.33	5.33	5.33	0.051
hsa-miR-30b-star_st	0.003	9.50E-01	0.995	5.90	5.90	5.90	0.219
hsa-miR-524-3p_st	-0.001	9.50E-01	0.995	5.32	5.32	5.32	0.053
hp_hsa-mir-3130-4_s_st	-0.001	9.51E-01	0.995	5.47	5.47	5.47	0.070
hp_hsa-mir-575_st	-0.001	9.51E-01	0.995	5.41	5.41	5.41	0.059
hp_hsa-mir-569_st	0.001	9.52E-01	0.995	5.38	5.38	5.38	0.058
hp_hsa-mir-876_st	0.001	9.53E-01	0.995	5.47	5.47	5.47	0.060
hsa-miR-548l_st	0.001	9.54E-01	0.995	5.58	5.58	5.58	0.089
hsa-miR-375_st	-0.012	9.55E-01	0.995	8.70	8.71	8.71	1.088
hp_hsa-mir-30d_x_st	-0.001	9.55E-01	0.995	5.65	5.65	5.65	0.073
hsa-miR-144_st	-0.001	9.57E-01	0.995	5.33	5.33	5.33	0.063
hp_hsa-mir-133b_st	0.001	9.57E-01	0.995	5.55	5.54	5.54	0.083
hsa-miR-211_st	-0.001	9.58E-01	0.995	5.43	5.43	5.43	0.075
hsa-miR-514b-5p_st	0.002	9.58E-01	0.995	5.83	5.83	5.83	0.191
hsa-miR-4266_st	-0.001	9.59E-01	0.995	5.46	5.46	5.46	0.063
hsa-miR-519c-3p_st	0.001	9.59E-01	0.995	5.28	5.28	5.28	0.058
hp_hsa-mir-4290_st	0.001	9.59E-01	0.995	5.52	5.52	5.52	0.064
hsa-miR-429_st	-0.003	9.60E-01	0.995	5.74	5.75	5.75	0.282
hsa-miR-573_st	0.000	9.60E-01	0.995	5.27	5.27	5.27	0.048
hp_hsa-mir-1264_st	-0.001	9.60E-01	0.995	5.40	5.40	5.40	0.054
hp_hsa-mir-342_st	0.001	9.61E-01	0.995	5.93	5.93	5.93	0.120
hsa-miR-1253_st	0.001	9.61E-01	0.995	5.36	5.36	5.36	0.061
hp_hsa-mir-592_st	0.001	9.61E-01	0.995	5.35	5.35	5.35	0.054
hsa-miR-941_st	-0.003	9.61E-01	0.995	5.89	5.89	5.89	0.263
hsa-miR-1233_st	-0.001	9.62E-01	0.995	5.48	5.48	5.48	0.092
hsa-miR-4322_st	0.004	9.62E-01	0.995	6.47	6.46	6.47	0.380
hsa-miR-4252_st	-0.001	9.63E-01	0.995	5.45	5.45	5.45	0.069
hp_hsa-mir-499_st	-0.001	9.63E-01	0.995	5.61	5.61	5.61	0.075
hsa-miR-519d_st	0.001	9.63E-01	0.995	5.34	5.34	5.34	0.059
hp_hsa-mir-548x_x_st	-0.001	9.64E-01	0.995	5.53	5.53	5.53	0.095
hsa-miR-298_st	-0.001	9.64E-01	0.995	5.74	5.74	5.74	0.123
hp_hsa-mir-331_st	0.001	9.65E-01	0.995	5.53	5.53	5.53	0.081
hsa-miR-937_st	-0.001	9.65E-01	0.995	5.45	5.45	5.45	0.067
hsa-miR-1295_st	0.000	9.66E-01	0.995	5.36	5.36	5.36	0.055
hsa-miR-518e_st	0.000	9.66E-01	0.995	5.27	5.27	5.27	0.054
hsa-miR-888-star_st	0.000	9.66E-01	0.995	5.26	5.26	5.26	0.054
hp_hsa-mir-320e_st	0.002	9.67E-01	0.995	6.17	6.17	6.17	0.184
hp_hsa-mir-766_st	-0.001	9.67E-01	0.995	5.46	5.46	5.46	0.076
hsa-miR-122_st	0.001	9.67E-01	0.995	5.35	5.35	5.35	0.065
hsa-miR-4304_st	0.001	9.67E-01	0.995	5.42	5.42	5.42	0.078

hsa-miR-770-5p_st	0.000	9.67E-01	0.995	5.37	5.37	5.37	0.058
hp_hsa-mir-2909_st	0.000	9.68E-01	0.995	5.32	5.32	5.32	0.058
hsa-miR-520f_st	-0.001	9.68E-01	0.995	5.43	5.43	5.43	0.066
hsa-miR-508-3p_st	0.000	9.69E-01	0.996	5.28	5.28	5.28	0.054
hsa-miR-3133_st	0.000	9.69E-01	0.996	5.36	5.36	5.36	0.063
hsa-miR-556-5p_st	0.000	9.70E-01	0.996	5.38	5.38	5.38	0.062
hsa-miR-569_st	0.000	9.70E-01	0.996	5.32	5.32	5.32	0.045
hp_hsa-mir-518d_x_st	0.000	9.72E-01	0.996	5.37	5.37	5.37	0.053
hp_hsa-mir-4254_st	0.000	9.72E-01	0.996	5.53	5.53	5.53	0.063
hp_hsa-mir-4329_st	0.000	9.72E-01	0.996	5.40	5.40	5.40	0.050
hsa-miR-338-3p_st	0.000	9.73E-01	0.996	5.29	5.29	5.29	0.053
hp_hsa-mir-602_st	-0.001	9.74E-01	0.997	5.77	5.77	5.77	0.129
hp_hsa-mir-3176_st	-0.001	9.74E-01	0.997	6.08	6.08	6.08	0.124
hsa-miR-548x_st	-0.001	9.75E-01	0.997	5.73	5.73	5.73	0.207
hsa-miR-518d-3p_st	0.000	9.75E-01	0.997	5.29	5.29	5.29	0.056
hp_hsa-mir-1972-1_s_st	0.000	9.76E-01	0.997	5.41	5.41	5.41	0.074
hsa-miR-506_st	0.000	9.77E-01	0.998	5.33	5.33	5.33	0.056
hsa-miR-325_st	0.000	9.77E-01	0.998	5.31	5.31	5.31	0.046
hsa-miR-4327_st	-0.002	9.79E-01	0.998	6.10	6.10	6.10	0.319
hsa-miR-4287_st	0.000	9.80E-01	0.998	5.43	5.43	5.43	0.076
hp_hsa-mir-128-2_x_st	0.000	9.80E-01	0.998	5.57	5.57	5.57	0.066
hp_hsa-mir-553_st	0.000	9.80E-01	0.998	5.31	5.31	5.31	0.051
hp_hsa-mir-4301_st	-0.001	9.80E-01	0.998	5.86	5.86	5.86	0.136
hsa-miR-1224-3p_st	0.000	9.81E-01	0.998	5.48	5.48	5.48	0.082
hp_hsa-mir-1302-7_st	0.000	9.81E-01	0.998	5.44	5.44	5.44	0.059
hp_hsa-mir-621_st	0.000	9.81E-01	0.998	5.40	5.40	5.40	0.058
hsa-miR-297_st	0.000	9.81E-01	0.998	5.34	5.34	5.34	0.079
hsa-miR-3122_st	0.001	9.81E-01	0.998	5.59	5.59	5.59	0.111
hsa-miR-187-star_st	-0.001	9.83E-01	0.998	5.70	5.70	5.70	0.121
hp_hsa-mir-320b-2_st	-0.001	9.83E-01	0.998	6.42	6.42	6.42	0.176
hsa-miR-550_st	0.001	9.83E-01	0.998	6.23	6.23	6.23	0.174
hsa-miR-653_st	0.000	9.84E-01	0.998	5.27	5.27	5.27	0.054
hsa-miR-4319_st	0.000	9.84E-01	0.998	5.40	5.40	5.40	0.069
hp_hsa-mir-9-3_st	0.000	9.84E-01	0.998	5.64	5.64	5.64	0.095
hsa-miR-140-3p_st	-0.002	9.85E-01	0.998	9.59	9.59	9.59	0.451
hsa-let-7a-2-star_st	0.000	9.86E-01	0.998	5.35	5.35	5.35	0.058
hsa-miR-1289_st	0.000	9.86E-01	0.998	5.32	5.32	5.32	0.056
hp_hsa-mir-3124_st	0.000	9.86E-01	0.998	5.46	5.46	5.46	0.072
hsa-miR-512-3p_st	0.000	9.87E-01	0.998	5.40	5.40	5.40	0.059
hsa-miR-1200_st	0.000	9.87E-01	0.998	5.33	5.33	5.33	0.053
hp_hsa-mir-3118-1_x_st	0.000	9.88E-01	0.998	5.30	5.30	5.30	0.045
hp_hsa-mir-376a-2_x_st	0.000	9.89E-01	0.998	5.40	5.40	5.40	0.059
hp_hsa-mir-4259_st	-0.001	9.89E-01	0.998	6.31	6.31	6.31	0.220
hsa-miR-147b_st	0.000	9.89E-01	0.998	5.37	5.37	5.37	0.077
hp_hsa-mir-1270-1_s_st	0.000	9.91E-01	0.998	5.33	5.33	5.33	0.051
hp_hsa-mir-181c_x_st	0.000	9.91E-01	0.998	5.58	5.58	5.58	0.076
hp_hsa-mir-198_st	0.000	9.91E-01	0.998	5.64	5.64	5.64	0.108

hsa-miR-548m_st	0.000	9.91E-01	0.998	5.28	5.28	5.28	0.056
hp_hsa-mir-3127_st	0.000	9.92E-01	0.998	5.61	5.61	5.61	0.096
hp_hsa-mir-429_st	0.000	9.92E-01	0.998	5.52	5.52	5.52	0.091
hp_hsa-mir-708_st	0.000	9.92E-01	0.998	5.67	5.67	5.67	0.083
hp_hsa-mir-10a_st	0.000	9.93E-01	0.998	5.38	5.38	5.38	0.064
hsa-miR-551b_st	0.000	9.93E-01	0.998	5.32	5.32	5.32	0.063
hsa-miR-1260_st	-0.001	9.95E-01	0.999	6.84	6.84	6.84	0.415
hsa-miR-337-3p_st	0.000	9.96E-01	1.000	5.39	5.39	5.39	0.063
hsa-miR-518f-star_st	0.000	9.97E-01	1.000	5.56	5.56	5.56	0.108
hp_hsa-mir-520b_x_st	0.000	9.98E-01	1.000	5.32	5.32	5.32	0.050
hsa-miR-647_st	0.000	9.98E-01	1.000	5.45	5.45	5.45	0.074
hp_hsa-mir-1289-2_st	0.000	9.99E-01	1.000	5.57	5.57	5.57	0.093
hsa-miR-200b_st	0.000	9.99E-01	1.000	9.26	9.26	9.26	0.702
hp_hsa-mir-185_st	0.000	9.99E-01	1.000	5.74	5.74	5.74	0.100
hsa-miR-519c-5p_st	0.000	1.00E+00	1.000	5.34	5.34	5.34	0.069
hp_hsa-mir-302b_x_st	0.000	1.00E+00	1.000	5.40	5.40	5.40	0.052
hsa-miR-374a-star_st	0.000	1.00E+00	1.000	5.40	5.40	5.40	0.065
